# Synthesis and Biological Evaluation of Termini-Modified and Cyclic Variants of the Connexin43 Inhibitor Peptide5

**DOI:** 10.3389/fchem.2022.877618

**Published:** 2022-09-13

**Authors:** Sin Hang Crystal Chan, Jarred M. Griffin, Connor A. Clemett, Margaret A. Brimble, Simon J. O’Carroll, Paul W. R. Harris

**Affiliations:** ^1^ School of Chemical Sciences, The University of Auckland, Auckland, New Zealand; ^2^ Centre for Brain Research, Department of Anatomy and Medical Imaging, Faculty of Medical and Health Sciences, University of Auckland, Auckland, New Zealand; ^3^ School of Biological Sciences, The University of Auckland, Auckland, New Zealand; ^4^ Maurice Wilkins Centre for Molecular Biodiscovery, The University of Auckland, Auckland, New Zealand

**Keywords:** connexin43, gap junctions, hemichannels, peptide5, peptide mimetics

## Abstract

Peptide5 is a 12–amino acid mimetic peptide that corresponds to a region of the extracellular loop 2 (EL2) of connexin43. Peptide5 regulates both cellular communication with the cytoplasm (hemichannels) and cell-to-cell communication (gap junctions), and both processes are implicated in neurological pathologies. To address the poor *in vivo* stability of native peptide5 and to improve its activity, twenty-five novel peptide5 mimetics were designed and synthesized. All the analogues underwent biological evaluation as a hemichannel blocker and as a gap junction disruptor, and several were assessed for stability in human serum. From this study, it was established that several acylations on the *N*-terminus were tolerated in the hemichannel assay. However, the replacement of the L-Lys with an *N*-methylated L-Lys to give H-VDCFLSRPTE-*N*-MeKT-OH showed good hemichannel and gap junction activity and was more stable in human serum. The cyclic peptide variants generally were not tolerated in either the hemichannel and gap junction assay although several possessed outstanding stability in human serum.

## 1 Introduction

Connexin43 (Cx43) is a member of the gap junction protein family and is found widely throughout the body (Oyamada et al., 2005). Cx43 plays a crucial role in tissue function, allowing cell-to-cell communication via the formation of gap junctions (Márquez-Rosado et al., 2012). Connexin proteins are membrane-bound proteins that consist of cytoplasmic N- and C-termini, four transmembrane domains, a cytoplasmic loop, and two highly conserved extracellular loops (EL1 and EL2) (Ribeiro-Rodrigues et al., 2017). Within the cell membrane, six connexin proteins oligomerize to form a connexon, and connexons on adjacent cell membranes align and dock via EL1 and EL2 to form an intercellular gap junction channel (Li et al., 1996). The gap junctions allow for the movement of ions and other small molecules between cells, which is crucial for cell function and survival (Krysko et al., 2005). Within the central nervous system, Cx43 gap junctions form a syncytium and provide a high degree of cell-to-cell communication, especially in endothelial and glial cells (Sarrouilhe et al., 2017). The injury or inflammation of the central nervous system leads to the upregulation and opening of unopposed connexons (hemichannels) (Xing et al., 2019). This creates a conduit between the intra- and extra-cellular environment allowing the movement of water and small molecules. The opening of connexin hemichannels negatively impacts a cell’s ability to osmoregulate (Quist et al., 2000; Rodríguez-Sinovas et al., 2007; Danesh-Meyer et al., 2012), leading to cell edema (Quist et al., 2000), excitotoxic neuronal cell death (Froger et al., 2010), glial cell activation (Li et al., 1996), vascular hemorrhage (De Bock et al., 2011), and ultimately the spread of secondary damage (Davidson et al., 2013.; Decrock et al., 2015). The opening of Cx43 hemichannels has been shown to be a major contributor to a number of CNS pathologies including retinal ischemia–reperfusion injury (Danesh-Meyer et al., 2012), spinal cord injury (O’Carroll et al., 2008; O’Carroll et al., 2013; Tonkin et al., 2015), preterm ischemia and asphyxiation (Davidson et al., 2012; Davidson et al., 2014), and infection (Eugenin et al., 2011). As such, targeting Cx43 hemichannels has potential as a treatment for neurological conditions.

One approach to target Cx43 hemichannels has been the use of peptidomimetics that target the EL1 and EL2 extracellular loops of Cx43 (gap26, gap27, and peptide5) (Evans, 2015) or intracellular regions of the protein (gap19, ACT-1) (D’hondt et al., 2013). The extracellular targeting of peptide5, H_2_N-VDCFLSRPTEKT-CO_2_H (O’Carroll et al., 2008), has been shown to selectively target either hemichannels or gap junctions in a concentration-dependent manner, with concentrations as low as 5 µM selectively inhibiting hemichannels and higher concentrations (e.g., 500 µM), inhibiting both gap junctions and hemichannels *in vitro*. This ability to selectively target pathological hemichannels whilst not interfering with the normal function of gap junctions presents the opportunity for therapeutic applications and Peptide5 has shown benefit in reducing inflammation, vascular breakdown, neuronal survival, and improved functional outcomes in a number of models of CNS injury such as brain ischemia–reperfusion injuries (Davidson et al., 2012, 2014), retinal injury and disease (Danesh-Meyer et al., 2012; Guo et al., 2016; Mugisho et al., 2019; Kuo et al., 2020), and spinal cord injury (O’Carroll et al., 2013; Mao et al., 2017). Despite these advances, further work is required to maximize both the efficacy and serum stability of peptide5-based therapeutic candidates to demonstrate their potential as agents to treat human diseases. Generally speaking, short native, unmodified peptides (< 20 amino acids) are rapidly cleared or degraded *in vivo* due to the action of proteases and/or filtration by the kidneys. The peptides are also limited in that they are unable to cross cell membranes due to polar functional groups and low lipophilicity leading to low bioavailability. A variety of chemical modifications have been employed to render peptides more suitable for human therapeutics (Muttenthaler et al., 2021) including lipidation (Zhang and Bulaj, 2012), glycosylation (van Witteloostuijn et al., 2016), cyclization (Bechtler and Lamers, 2021), and modification of individual amino acids (Gentilucci et al., 2010); these improvements are exemplified by the ever-increasing clinical application of peptides for use in cancer, infectious diseases, and diabetes (D’Aloisio et al., 2021; de la Torre and Albericio, 2020).

**FIGURE 1:**
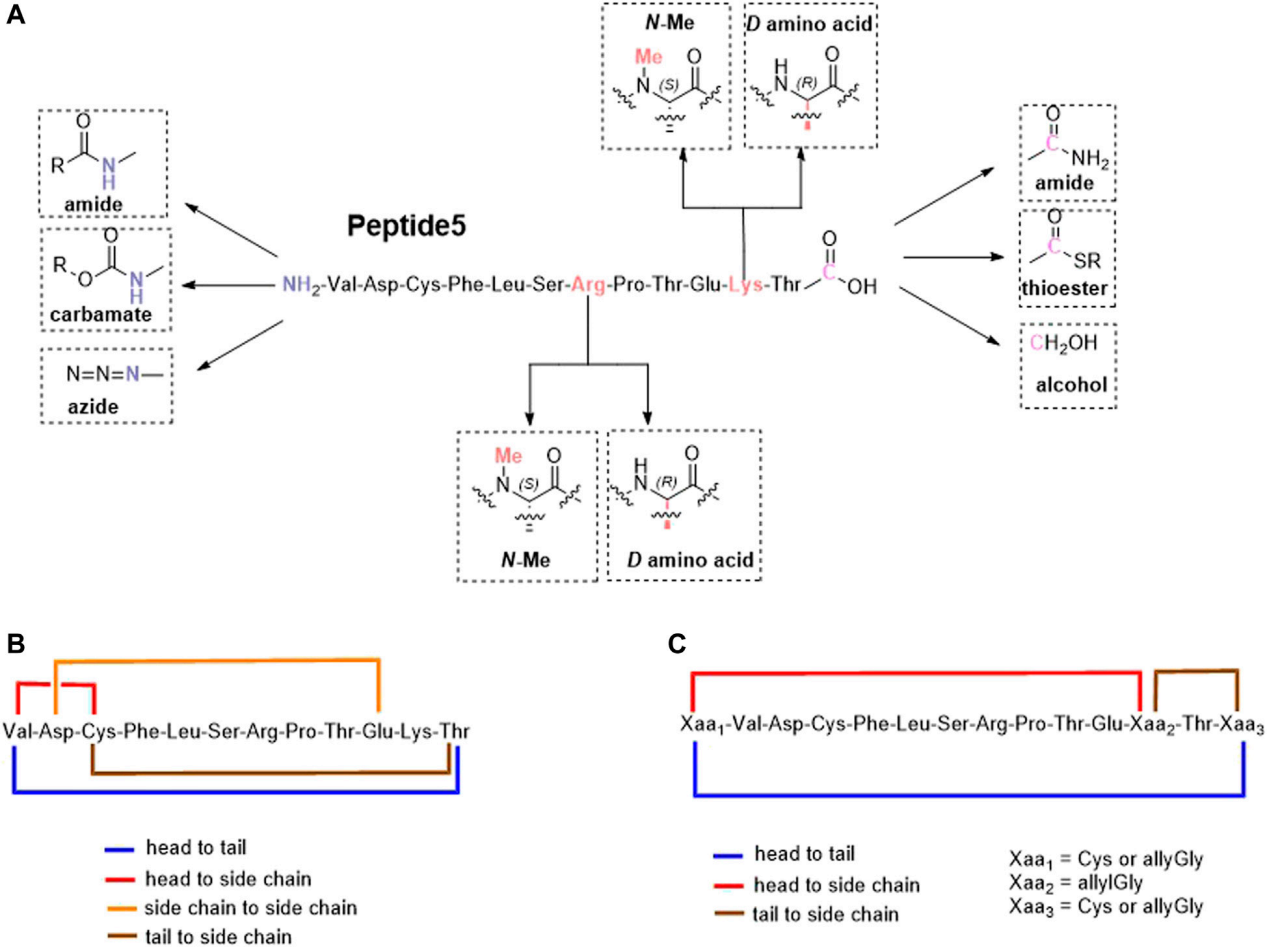
Proposed linear **(A)** and cyclic variants using either existing amino acids **(B)** or introduced handles **(C)** of the connexin inhibitory peptide, peptide5.

We report herein our efforts in designing, synthesizing, and evaluating 25 novel linear and cyclic peptide5 analogues (Figure 1) as inhibitors of connexin hemichannels and gap junctions as well as assessing their stability in human serum. All peptides were readily generated via solid-phase peptide synthesis. It was found that most of the modifications to peptide5 resulted in the loss of activity in both a hemichannel inhibition assay and a gap junction dye spread assay. Several linear peptide5 analogs exhibiting *N*-terminal acylation did show activity comparable to peptide5 in their ability to block hemichannels, while all the cyclic peptide5 congeners failed to demonstrate significant hemichannel activity compared to the peptide5 control. Several D- or *N*-methylated variants of linear peptide5 were both shown to have gap junction activity similar to that of peptide5 and were significantly more stable in human serum.

## 2 Results

In our previous studies on peptide5 (**1**), we constructed a 42-peptide library of *S*-lipidated linear analogues modified at six different amino acid positions (Yang et al., 2020) that were informed by the results of our preliminary alanine scan study (Kim et al., 2017a). While it was shown that lipidation at certain residues was tolerated and, in some cases improved connexon hemichannel blocking, (rat) serum stability of the lipidated peptides was not improved relative to peptide5 which had a short half-life of just 5 min. This suggested that the individual amino acids are responsible for the degradation of peptide5 and that modification of specific amino acids was required to improve the stability, whilst maintaining connexin blocking activity.

Lipidation is only one of several methods employed to improve the stability, permeability, and half-life of peptides that include the incorporation of non-canonical amino acids, D- and *N*-methylated amino acids, and modification of the C- and *N*-termini including cyclization (Erak et al., 2018). Cyclization, in particular, is also known to rigidify the peptide backbone, reducing the number of possible conformers and promoting binding to its intended target (Philippe et al., 2021). An exquisite example of this is the FDA-approved macrocyclic peptide cilengitide which displayed a 10-fold increase in binding compared to its linear form. (Kumagai et al., 1991). Based on the aforementioned considerations, we proposed to synthesize peptide5 analogues as depicted in Figure 1. These analogues included modification of both the C- and *N*-termini and replacement of enzymatically susceptible Arg and Lys by their D- and *N*-methylated variants. The cyclic peptide5 analogues were prepared using the side chains and the C- and *N*-termini already present in the primary sequence or employing introduced chemical handles to affect cyclization.

**TABLE 1:** A summary of all linear and cyclic variants of the connexon43 inhibitor peptide. Peptide5 prepared. The chemical changes are highlighted in bold.

Number	Structure
1 (Peptide5)	H_2_N-VDCFLSRPTEKT-CO_2_H
2	**CH**_**3**_ **CH**_**2**_ **OCO**-NH-VDCFLSRPTEKT-CO_2_H
3	**(CH**_**3**_ **)**_**2**_ **CH**_**2**_ **CH**_**2**_ **OCO**-NH-VDCFLSRPTEKT-CO_2_H
4	**PhCO**-NH-VDCFLSRPTEKT-CO_2_H
5	**CH**_**3**_ **CH**_**2**_ **CH**_**2**_ **CO**-NH-VDCFLSRPTEKT-CO_2_H
6	**N**_**3**_-VDCFLSRPTEKT-CO_2_H
7	H_2_N-VDCFLSRPTE-***N*-Me-K**T-CO_2_H
8	H_2_N-VDCFLS-***N*-Me-R**PTEKT-CO_2_H
9	H_2_N-VDCFLSRPTE**k**T-CO_2_H
10	H_2_N-VDCFLS**r**PTEKT-CO_2_H
11	**CH**_**3**_ **CH**_**2**_ **OCO**-NH-VDCFLSRPTEKT-**CONH**_**2**_
12	**(CH**_**3**_ **)**_**2**_ **CH**_**2**_ **CH**_**2**_ **OCO**-NH-VDCFLSRPTEKT-**CONH**_**2**_
13	**PhCO**-NH-VDCFLSRPTEKT-**CONH**_**2**_
14	**CH**_**3**_ **CH**_**2**_ **CH**_**2**_ **CO**-NH-VDCFLSRPTEKT-**CONH**_**2**_
15	H_2_N-VDCFLSRPTEKT-**CH**_**2**_ **OH**
16	H_2_N-VDCFLSRPTEKT-**COSCH**_**2**_ **CH**_**2**_ **Gly-CO**_**2**_ **H**
21	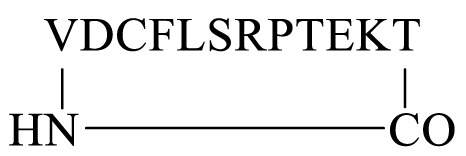
22	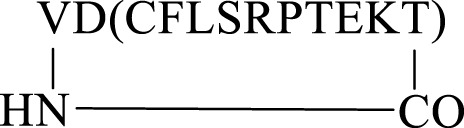
25	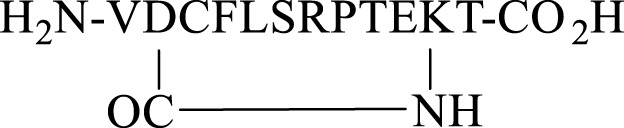
28	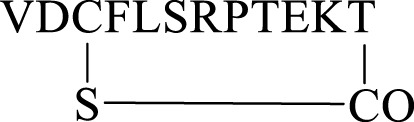
32	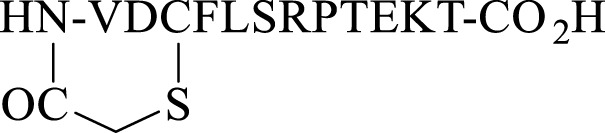
34	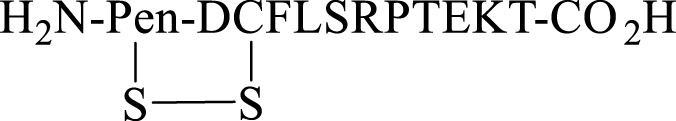
37	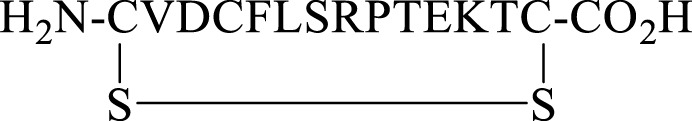
39	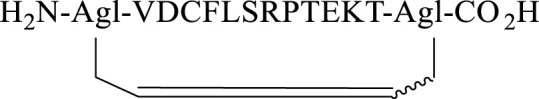
40	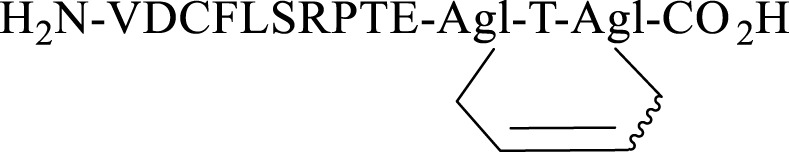
42	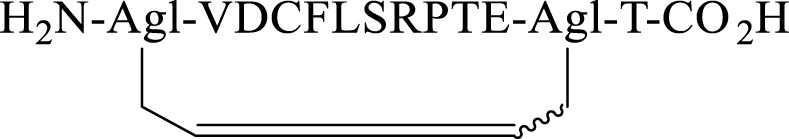

### 2.1 Synthesis of linear peptides modified at the C- and *N*-termini, and incorporation of non-proteogenic amino acids

**FIGURE 2:**
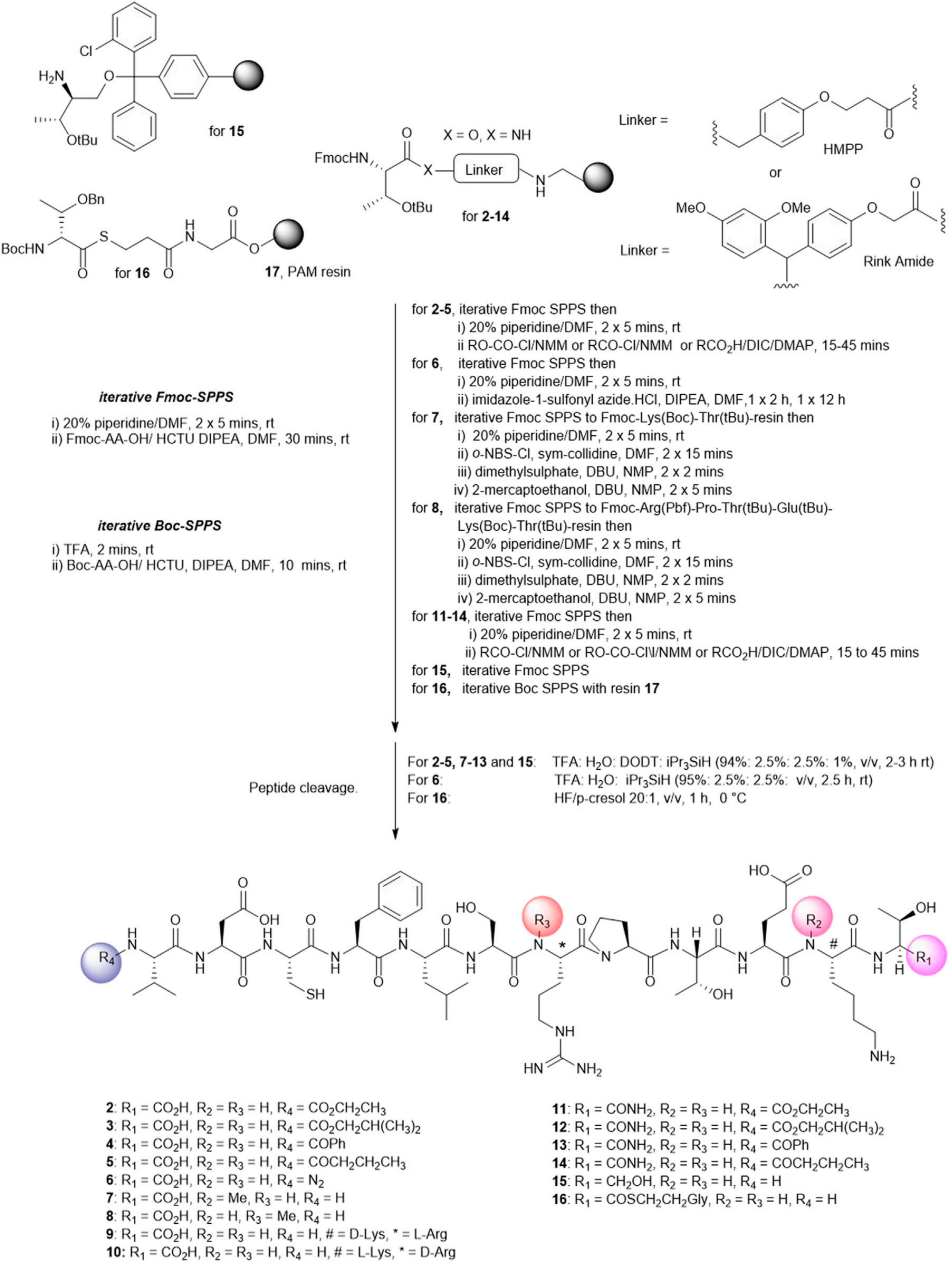
Solid phase synthesis strategy to prepare linear Peptide5 analogues modified at the C- and Ntermin and D and N-Me variants.

We commenced by designing and synthesizing a set of linear peptides that 1) were modified at the *N*-terminus by acylation or by converting the amine group to an azide; 2) replaced the C terminal carboxy group with a carboxamide and capping of the *N*-terminus by acylation; 3) replaced the trypsin-susceptible amino acids, Arg-7 and Lys-11 with their D- or *N*-methylated congeners; and 4) replaced the C terminal carboxy group with a carboxamide, a thioester, or an alcohol. The synthesis of these sixteen analogues is depicted in Figure 2. These modifications were in line with our alanine scan data which demonstrated that replacement of each amino acid by alanine had little effect on gap junction uncoupling (Kim et al., 2017b).

The analogues of Peptide5 capped at the N-terminus (**2–5**) were readily prepared on Fmoc-Thr (tBu) esterified hydroxymethylphenoxypropionic acid (HMPP) linker functionalized on aminomethyl polystyrene resin. Following the peptide chain assembly, the final Fmoc group was removed and the N-terminal amino group reacted with either ethyl chloroformate/*N*-methylmorpholine (NMM), isobutyl chloroformate/NMM, benzoyl chloride/NMM, or butyric acid in the presence of the acylating reagents diisoproylcarbodiimide (DIC)/catalytic dimethylaminopyridine (DMAP) to effect amidation. In all cases, a single treatment was required, and all compounds were recovered in >95% purity following HPLC purification (see Supplementary Material).

For azide analogue **6**, the azide transfer reagent, imidazole-1-sulfonyl azide.HCl (Goddard-Borger and Stick, 2007), reacted directly with the *N*-terminal amino group of the resin-bound peptide. In this case, two treatments were required for complete conversion, and importantly, no thiol scavenger (DODT) was used for the peptide cleavage cocktail to mitigate the potential reduction of the azide to the amine.

*N*-methylated Arg-7 and Lys-11 peptides (**7** and **8**) could be accessed by adopting the on-resin *N*-methylation protocol developed by Kessler (Chatterjee et al., 2012). Following Fmoc removal at the amino acid site of putative N-methylation, the amino group was nosylated affording a sulfonamide. Methylation of the sulfonamide (dimethylsulfate/DBU) and removal of the nosyl group (2-mecaptoethanol/DBU) yielded the *N*-methylated peptides. The elongation of the peptide sequence, resin cleavage, and HPLC purification gave **7** and **8** in high purity. No difficulties were encountered when coupling the subsequent Fmoc amino acid to the sterically hindered, resin-bound *N*-methylated amino acid. For the synthesis of peptide5 containing d-amino acids at Lys-11 (**9**) and Arg-7 (**10**), Fmoc-D-Lys (Boc)-OH or Fmoc-D-Arg (Pbf)-OH was used at the appropriate positions during the Fmoc SPPS assembly.

Finally, the peptide analogues modified at the C-terminus (**11**–**16**) were prepared on the appropriate linker resin. Carboxamides (**11–14**) were assembled on a Rink amide linker using Fmoc SPPS and were *N*-terminally modified according to the protocols outlined for peptides **2–5**. C-terminal alcohol **15** was directly accessible from the 2-chlorotrityl resin functionalized with *tert*-butyl–protected Fmoc-threoninol using Fmoc SPPS. Thioester **16** was best prepared using the thioester generating resin **17** (Camarero et al., 2000) and employing Boc SPPS to avoid aminolysis of the thioester linkage, a known concern when employing piperidine as an Fmoc deblocking reagent during Fmoc synthesis (Li et al., 1998).

#### 2.1.1 Synthesis of Cyclic Peptides Using Pre-Existing Amino Acids

**FIGURE 3:**
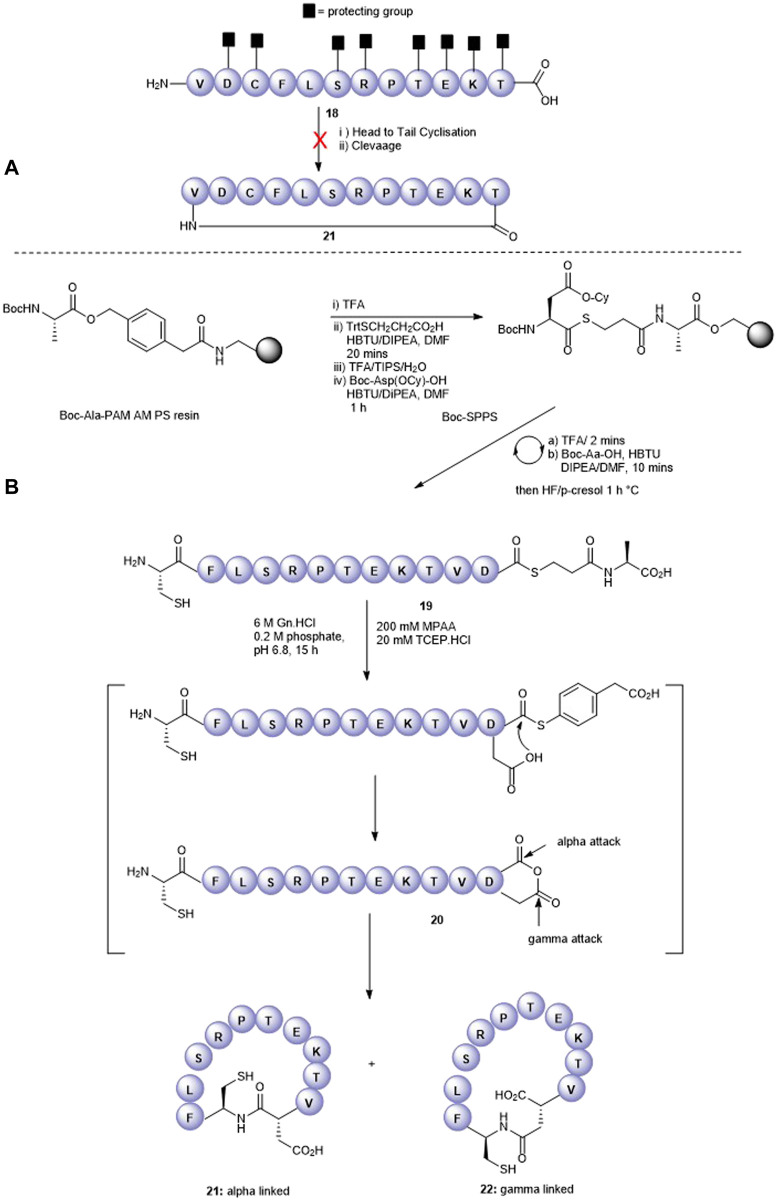
Unsuccessful cyclization attempt to prepare cyclic peptide5 **21 (A)** and the successful strategy employing native chemical ligation **(B)**.

We next attempted to cyclize the C- and *N*-termini of the maximally side-chain–protected peptide **18** to obtain cyclic analogue **21**. In turn, protected peptide **18** was prepared on the hyper acid-labile 2-chloro trityl resin (Figure 3A). However, using HBTU or BOP-Cl/DMAP as condensation reagents was unsuccessful and none of the expected head-to-tail cyclic products was detected after side-chain deprotection and LC-MS analysis. This is most likely due to the difficult cyclization site that contains the bulky, secondary amino acids, valine and threonine. We therefore repositioned the cyclization site to the Asp-Cys junction as we anticipated that using native chemical ligation (NCL) (Dawson et al., 1994), the chemoselective amide bond-forming reaction between a cysteinyl peptide and peptide thioester would furnish the desired cyclic peptide **21** (Figure 3B).

The linear thioester precursor, CFLSRPTEKTVD-COS-CH_2_CH_2_Ala-OH **19**, was prepared satisfactorily using Boc SPPS and then directly subjected to NCL conditions (0.2 M phosphate, 6 M Gn.HCl, 20 mM TCEP.HCl, and 200 mM 4-mercaptophenyl acetic acid [MPAA]) for 15 h which afforded a separable mixture (2:1) of the desired cyclic product **21 (**calc (M + H)^+^ 1378.6, found 1378.4) and undesired product **22 (**calc (M + H)^+^ 1378.6, found 1378.5) (Figure 3B). This was not unexpected as ligation between Asp and Cys has been reported (Villain et al., 2003) to result in the formation of the unnatural β amide bond as a by-product (20%–30%) (Dang et al., 2013) by the formation of an intermediate succinic anhydride **20** which can undergo aminolysis at either carbonyl group. Fortunately, in the present work, the undesired isomeric by-product **22** was easily separated from the desired product **21** using RP-HPLC.

**FIGURE 4:**
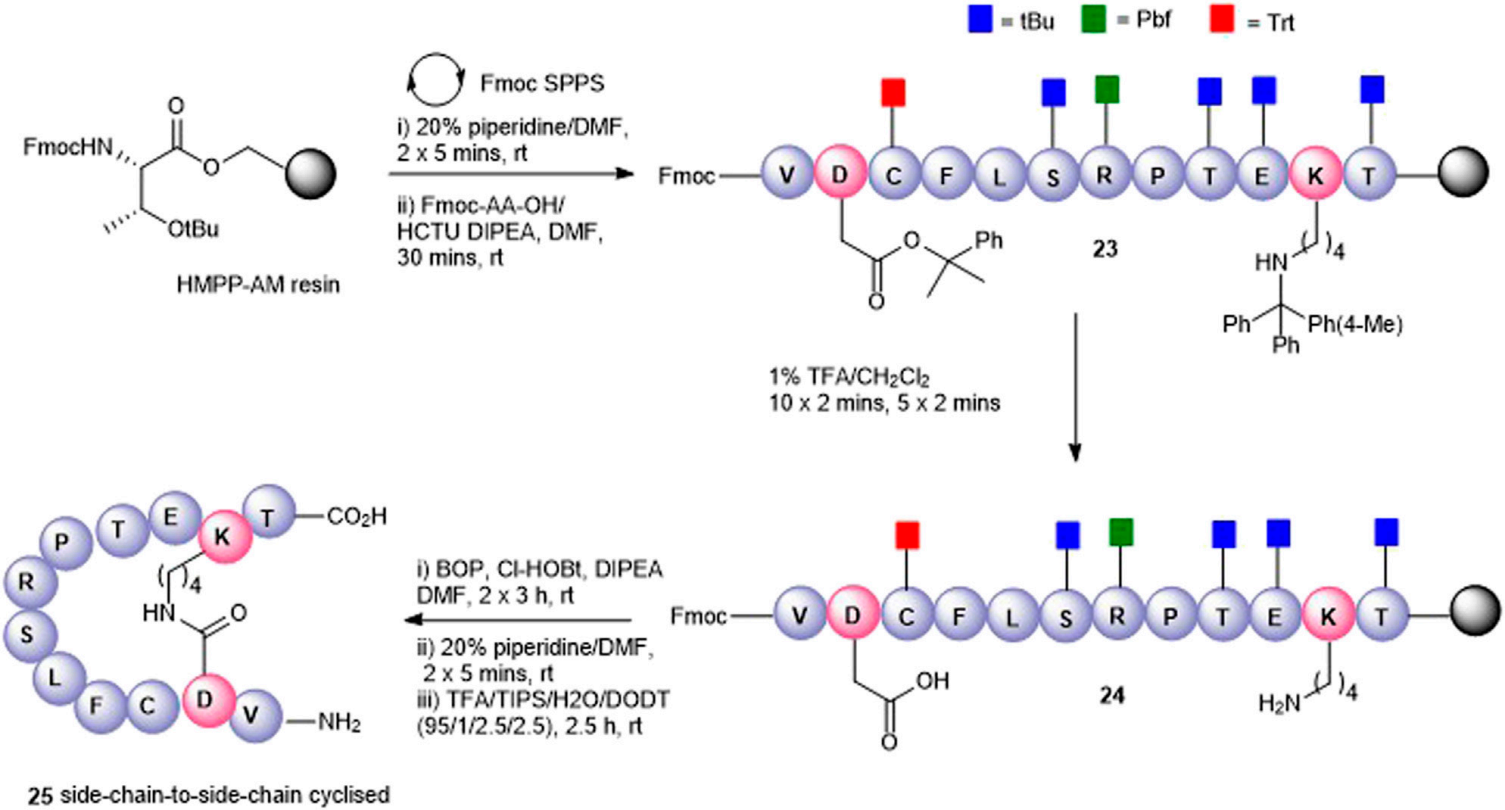
Synthesis of the Asp2-Lys11 side-chain–to–side-chain cyclized peptide5 (**25**).

Having successfully prepared the head-to-tail cyclic variant of peptide5 (**21**), we next focused on constraining the peptide via its amino acid side chains. An alanine scan (Kim et al., 2017a) revealed that all amino acids could be modified with no significant loss in gap junction uncoupling, although some replacements abolished hemichannel blocking activity. We first pursued the preparation of an amide linkage between Asp and Lys (Figure 4). This is most easily accomplished on the solid support whereby the pseudo-dilution effect minimizes unwanted dimerization or oligomerization that prevails under solution-phase conditions. Furthermore, the coupling reaction can be conveniently monitored by the Kaiser test that determines any free primary amines.

To maximize synthetic efficiency, the 4-methyltriphenylmethyl (Mtt)– and 2-phenylisopropyl (2-Ph^*i*^Pr)–protecting groups were used on the Lys and Glu side chains, respectively, as these can be removed simultaneously with diluted TFA, selectively unmasking the amine and carboxylic acid functionalities for subsequent lactamisation leaving other side-chain–protecting groups unmodified. Thus, resin-bound peptide **23** was assembled by Fmoc SPPS on Fmoc-Thr (O^t^Bu)-HMPP resin using 20% piperidine in DMF for Fmoc removal and HCTU/DIPEA for coupling. Orthogonally protected Fmoc-Lys (Mtt)-OH and Fmoc-Asp(O-2-Ph^*i*^Pr) were incorporated uneventfully and the Mtt and 2-Ph-^*i*^Pr side-chain–protecting groups both cleaved upon repetitive treatments with 1% TFA in CH_2_Cl_2_ to give **24**. The key macrolactamization step was then explored on resin using PyBOP/Cl-HOBt, DIC/HOAt, DIC/Oxyma, or BOP/Cl-HOBt as condensation reagents. Employing PyBOP- or DIC-based couplings led to aspartimide formation (PyBOP), no reaction or incomplete reaction (DIC/HOAt), or a complex mixture (DIC/Oxyma). PyBOP/Cl-HOBt (2 × 3 h) proved to be the optimal coupling reagents and affording the desired cyclic peptide **25** in >90% conversion as judged by LC-MS (calc (M + H)^+^ 1378.6, found 1378.6) that was easily purified by RP-HPLC.

**FIGURE 5:**
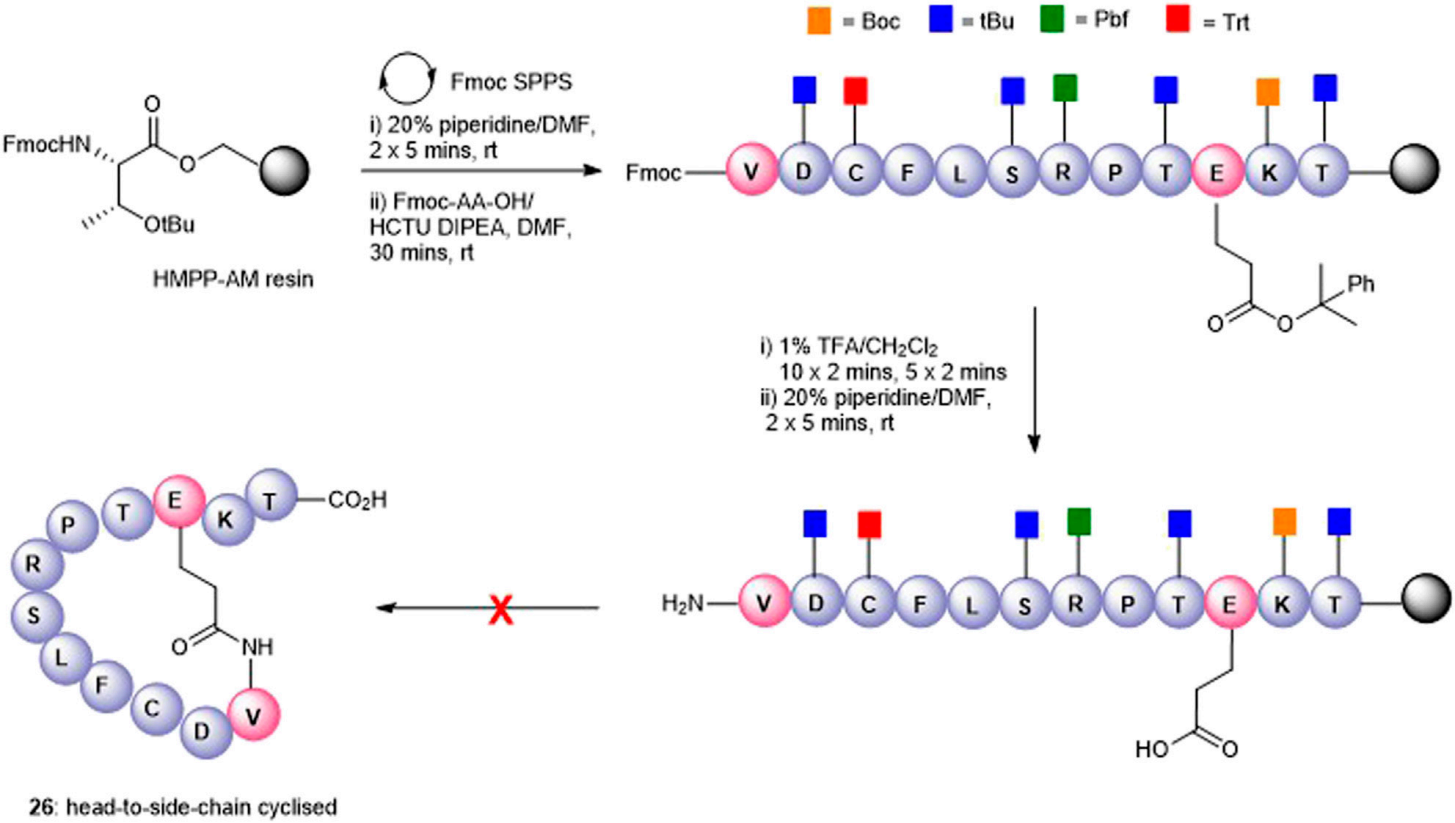
Unsuccessful synthesis of Glu10-Val1 side-chain-to-head cyclized peptide5 **26**.

The attempts to form the head-to-side-chain macrolactamisation **26** by on-resin coupling of the *N*-terminal amino group and the side-chain carboxylic acid of Glu using the orthogonal protecting strategy as described above met with failure (Figure 5). Extensive oligomerization was observed using several different coupling conditions and the use of a lower loading resin, or PEG-based resins had little effect on the success of the reaction.

**FIGURE 6:**
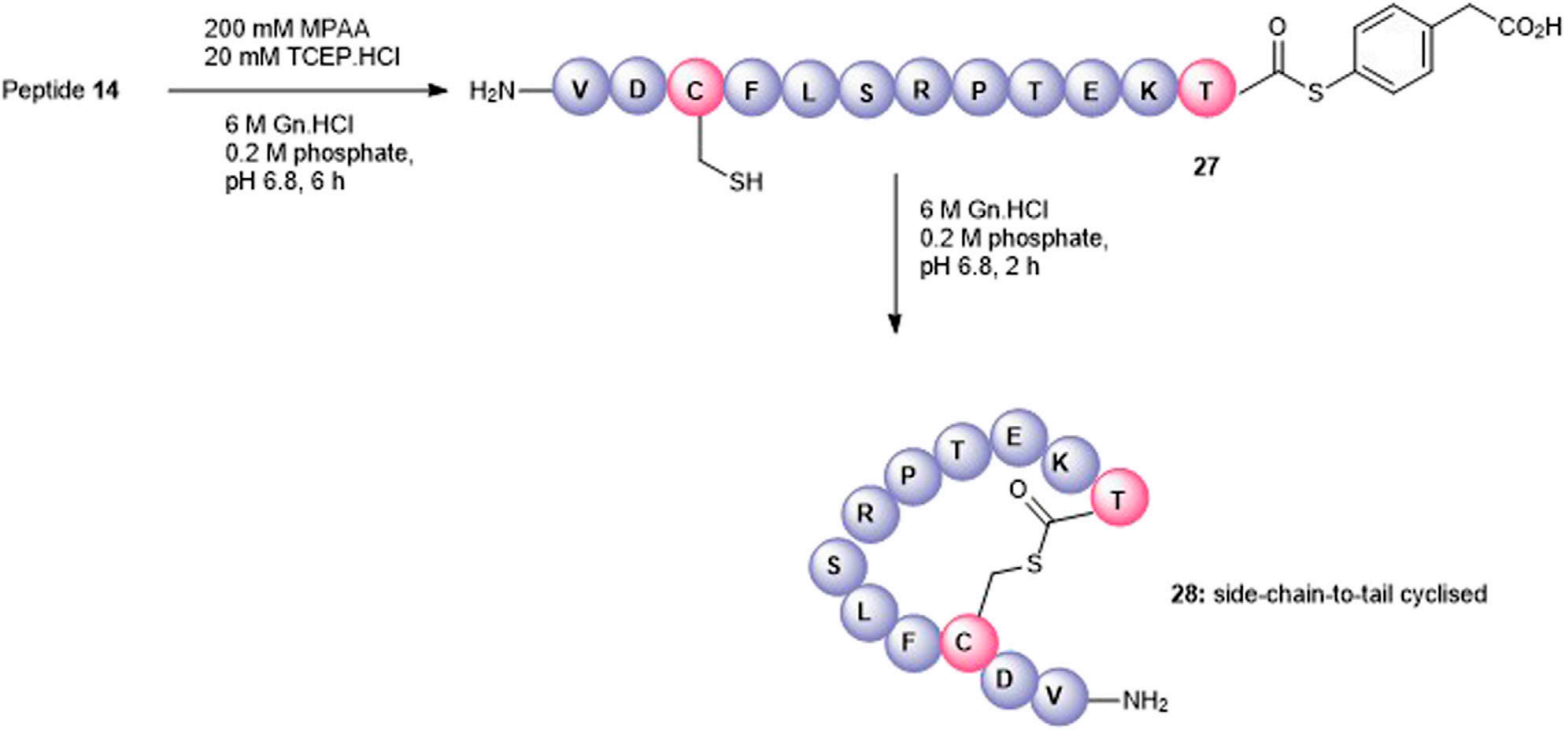
Synthesis of the Cys3-Thr12 thioester-linked side-chain-to-tail cyclized peptide5 (**28**).

Satisfactorily, a side-chain-to-tail mode of macrocyclization was effected by the selective condensation of Cys with the C-terminal threonyl aryl thioester at neutral pH (Figure 6). The precursor and less reactive alkyl thioester, **14**, was quantitatively transformed into the more reactive aryl thioester **27** (calc (M+2H)^2 +^ 773.8, found 773.4) using the water-soluble aryl thiol, MPAA (Johnson and Kent, 2006) at pH 6.5 after 6 h and isolated by SPE. We anticipated that at neutral pH in the absence of added thiols, the cysteine thiol would undergo transthioesterfication with the activated C-terminus in an irreversible manner. Thus, **27** (2 mM) was dissolved in 0.2 M phosphate/6 M Gn.HCl, at pH = 6.7, and the cyclization was monitored by RP-HPLC. After 2 h, the linear polypeptide was quantitatively converted into the side-chain-to-tail macrocyclic thiolactone **28** (calc (M+2H)^2 +^ 689.8, found 689.4) as the sole product that was easily recovered by RP-HPLC.

#### 2.1.2 Synthesis of Cyclic Peptides Using Introduced Handles

Disulfide bonds, the covalent linkage formed between two cysteines, are naturally occurring structure-stabilizing motifs that are important for maintaining the structure and function of proteins and peptides. Not surprisingly, they have been also been employed for peptide cyclization to confer structural rigidity on synthetic peptides, leading to higher affinity to target receptors (Góngora-Benítez et al., 2014). Peptide5 contains a single cysteine residue (Cys3) that could be used as a cyclization handle, though our alanine scanning data suggested that this residue could not be altered without some loss of hemichannel activity. However, the *N*-terminal valine (Val1) residue could be modified as the substitution of Val1 to Ala caused little effect in activity (Kim et al., 2017b) in both hemichannel and gap junction assays. Furthermore, a truncated peptide5, SRPTEKT, was equally effective as a gap junction inhibitor, suggesting that the *N*-terminal residues, VDCFL, could be modified. Therefore, we used Cys3 to 1) create a thioether bridge by intramolecular chemo-selective alkylation of Cys-SH with the *N*-terminus capped with a 2-bromoacetyl group or 2) to form a disulfide bond between Cys-SH and β,β-dimethylcysteine (penicillamine, Pen) introduced as a substitute for Val1. A disulfide-linked head-to-tail cyclic peptide was also proposed by introducing a cysteine adjacent to both the C- and *N*-termini as cyclization handles enabling subsequent oxidation to the disulfide.

**FIGURE 7:**
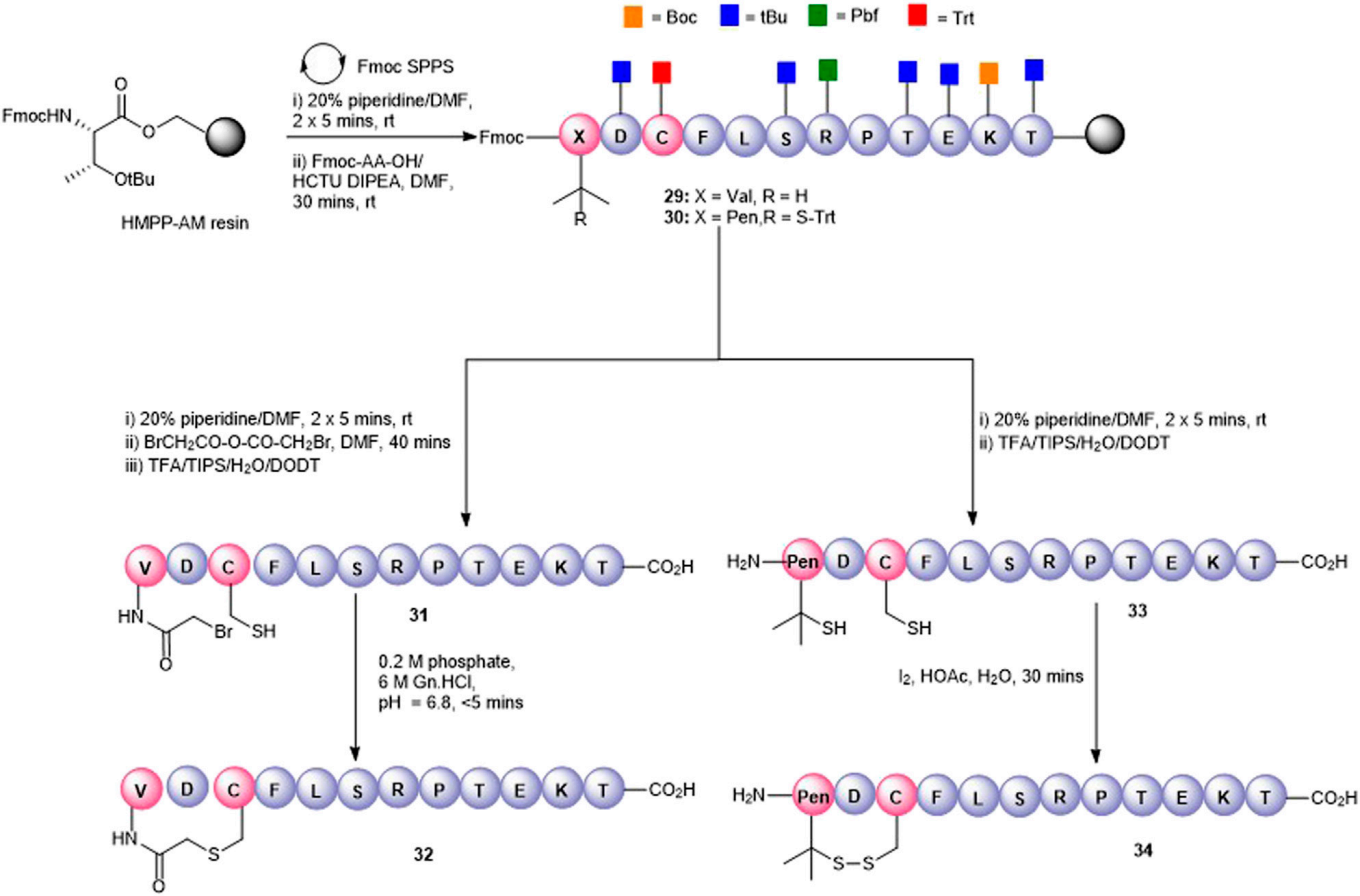
Synthesis of Val1-Cys3 thioester-linked cyclized peptide5 (**32**) and a Pen1-Cys3 disulfide-bonded cyclic peptide5 (**34**).

The thioether macrocycle (**32**, Figure 7) was prepared by acylation of the resin-bound *N*-terminus (**29**) with bromoacetic acid anhydride (Robey and Fields, 1989), followed by cleavage from the resin to afford the *N*-bromoacetylated peptide **31** (calc (M+2H)^2 +^ 759.2, found 758.3). Spontaneous cyclization then took place by exposing the crude peptide to an aqueous buffer (pH = 6.9) to afford the thioether **32** (calc (M+2H)^2 +^ 717.8, found 718.4) in 20% yield.

To effect the disulfide head-to-side-chain–linked macrocycle (**34**, Figure 7), commercially available Fmoc-Pen (Trt)-OH was coupled with the resin-bound peptide in lieu of Fmoc-Val to afford **30**. Following final Fmoc removal and recovery of the peptide by cleavage from the resin, the crude peptide (**33**) was subjected to several oxidative folding conditions. Stirring in aqueous buffer (0.1 M tris, pH = 7.9) at room temperature or heating at 37°C gave no reaction or undesired by-products. Using 4,4′-dithiodipyridine (DTDP) in DMSO or the more reactive 5,5′-dithiobis-(2-nitrobenzoic acid) (DTNB) in 4:1 HOAc:water gave mostly unoxidized peptide after 24 h and unknown by-products. Employing I_2_ in 4:1 HOAc:water resulted in quantitative conversion to the expected disulfide **34** (calc. M+2H)^2 +^ 713.8, found 713.4) which was consistent with the expected loss of 2 Da.

**FIGURE 8:**
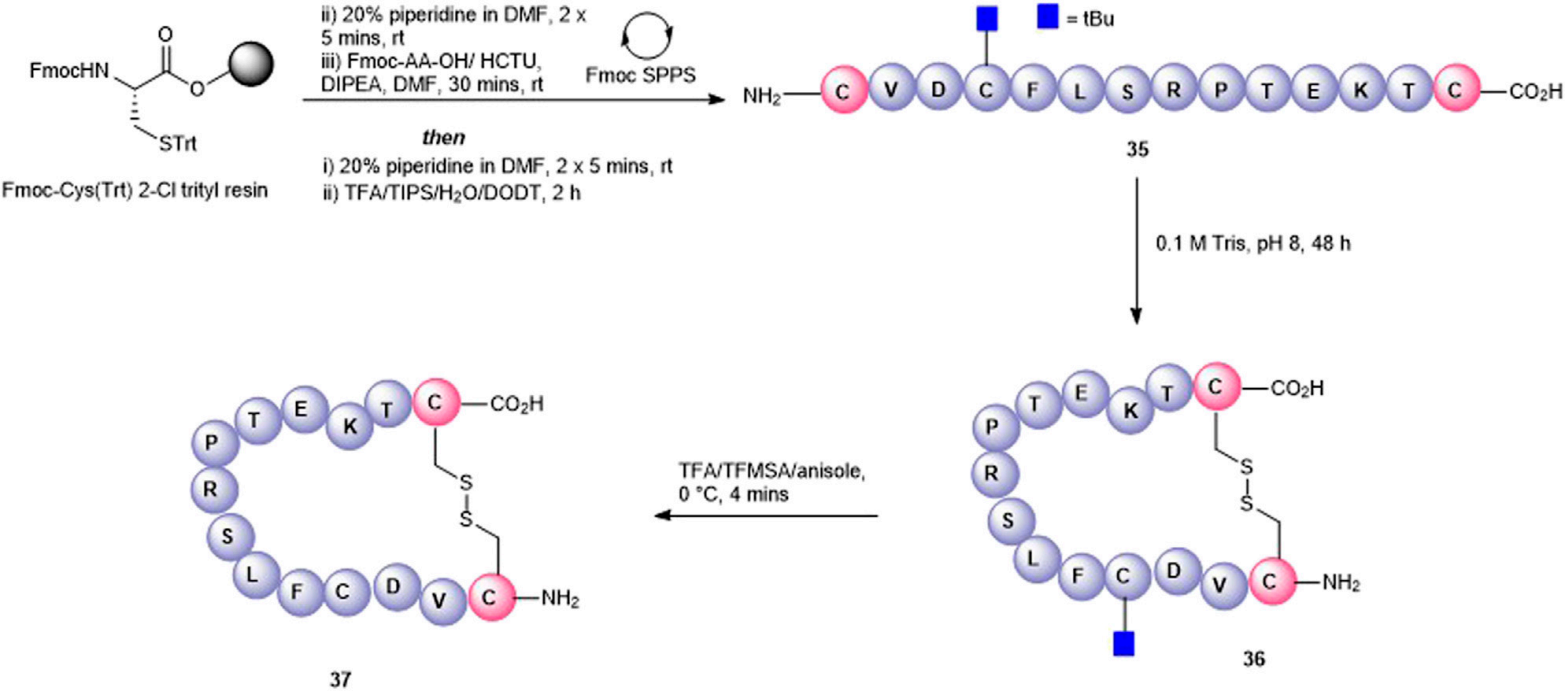
Synthesis of a disulfide-linked head-to-tail macrocycle **37** via introduction of Cys at the C- and *N*-termini.

Finally, a disulfide head-to-tail peptide5 analogue (**37,** Figure 8) was accessed from peptide **35** prepared on a 2-chlorotrityl resin. In this case, Cys3 required orthogonal protection on the sulfhydryl so that the disulfide bond between the two Cys at either end of the peptide could be formed selectively. In contrast to the oxidation of **33**, oxidation of **35** proceeded readily in the air (0.1 M Tris buffer, pH = 8.1) with no exogenous reagents required to afford **36** (calc (M+2H)^2 +^ 825.9, found 825.5). A noticeable shift in HPLC retention time (see SI) was observed for the disulfide-containing peptide **36** compared to the free thiol-containing peptide **35**. The subsequent unmasking of Cys3 with TFA/TFMSA afforded **37** (calc. M+2H)^2 +^ 800.9, found 800.4) in 22% yield.

Olefin metathesis has been demonstrated as an exquisite way to prepare cyclic peptides containing all carbon bridges (coined “stapling”) (Miller et al., 1996) and is most often used to stabilize alpha-helical peptides (Schafmeister et al., 2000). In our case, we employed olefin metathesis to prepare three further cyclic peptide5 variants; a head-to-tail analogue **39**, a head-to-side chain analogue **42**, and a tail-to-side-chain analogue **40**. Fmoc-(*S*)-allylGly (AgI), a commercially available chiral alkenyl amino acid, was employed to introduce the required olefin at positions adjacent to the *N*- and C-termini and at Lys-11, a position known to tolerate substitution in gap junction unblocking.

**FIGURE 9:**
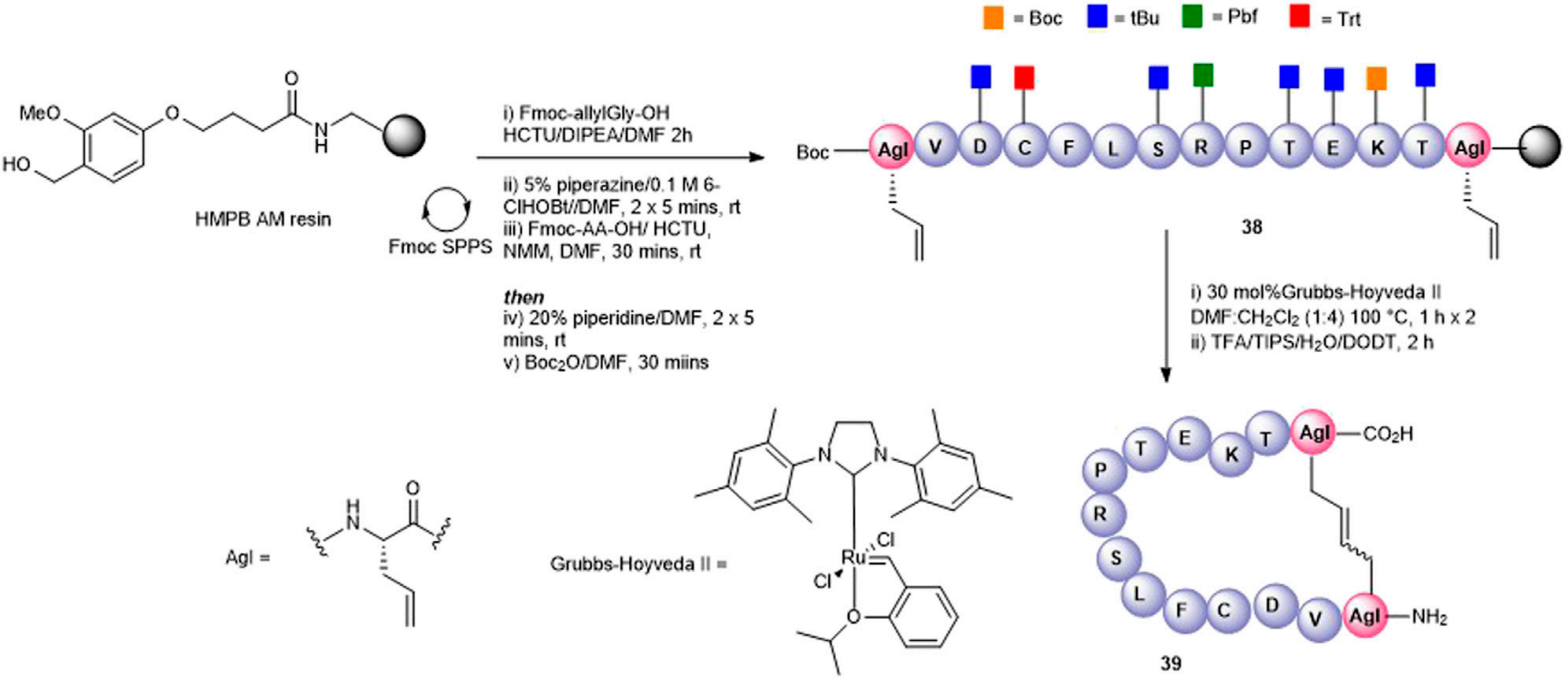
Synthesis of an all-carba head-to-tail macrocycle **37** via the introduction allylGly (AgI) at the C- and *N*-termini and ring-closing metathesis.

The head-to-tail cyclic variant **39** was obtained through ring-closing metathesis (RCM) of peptidyl-resin **38** containing two allylglycine (Agl) residues adjacent to the *N*- and C-terminal amino acids using 30 mol% Hoveyda-Grubbs’ II catalyst in DMF/CH_2_Cl_2_ 100°C for 2 h (Figure 9). These conditions had previously been employed by us in the synthesis of carba analogues of dianthin G (Amso et al., 2016). The side-chain–protecting groups were removed using the standard TFA cleavage cocktail to afford crude product. Although the expected product **39** was observed by HPLC and MS (calc [M + H]^+^ 1561.8 found 1560.0), the complicated HPLC profile precluded subsequent purification. The reaction conditions were modified to two 1-h treatments at 100°C using fresh 30 mol% Hoveyda-Grubbs’ II catalyst in each instance. This resulted in a better HPLC profile and the cyclic product (**39**) was easily purified.

**FIGURE 10:**
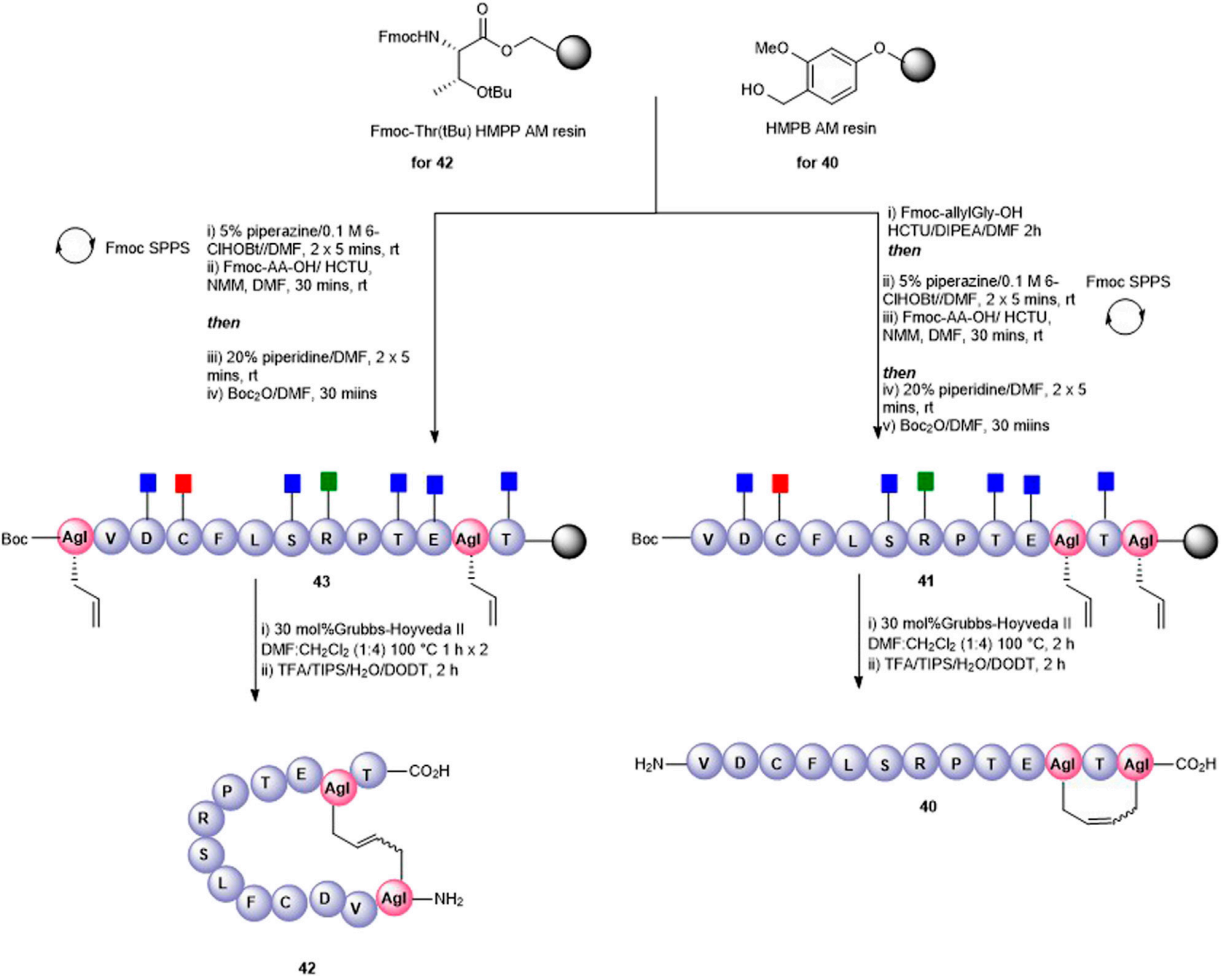
Synthesis of an all-carba head-to-side-chain macrocycle **42** and a tail-to-side-chain variant **40** by ring-closing metathesis.

A tail-to-side-chain carba analogue (**40**) was prepared by RCM of peptidyl resin (**41**) containing an allylGly at the C-terminus and at Lys11 (Figure 10). A single treatment using 30 mol% Hoveyda–Grubbs’ II catalyst at 100°C for 2 h afforded the cyclized side-chain–protected product which upon cleavage from the resin and side-chain deprotection using the TFA cocktail yielded the cyclic RCM product **40** (calc [M + H]^+^ 1433.7, found 1433.5) which was recovered by semi-preparative HPLC. The clean HPLC profile in this instance may be reflective of the smaller macrocyclic ring formed in this analogue.

Finally, a head-to-side-chain variant (**42**) was accessed through RCM of peptidyl resin **43** containing an allylGly at Lys11 and adjacent to the *N*-terminus (Figure 10). In this case, two 1-h treatments at 100°C each in the presence of 30 mol% Hoveyda–Grubbs’ II catalyst were required for complete conversion to the desired macrocycle **42**. Following resin cleavage, an extremely clean HPLC chromatogram was observed (see SI) with the MS of the major peak being consistent for **42** (calc [M + H]^+^ 1434.7, found 1434.6).

For the three carba analogues **39**, **42**, and **40** obtained by RCM, it was postulated that a mixture of *E* and *Z* isomers were present. No attempt was made to determine the *E/Z* ratio or to separate out each individual isomer, and the analogues underwent biological testing as the presumed *E/Z* mixture.

### 2.2 Biological assay of linear and cyclic peptides to inhibit gap junctions and hemichannel opening

**FIGURE 11:**
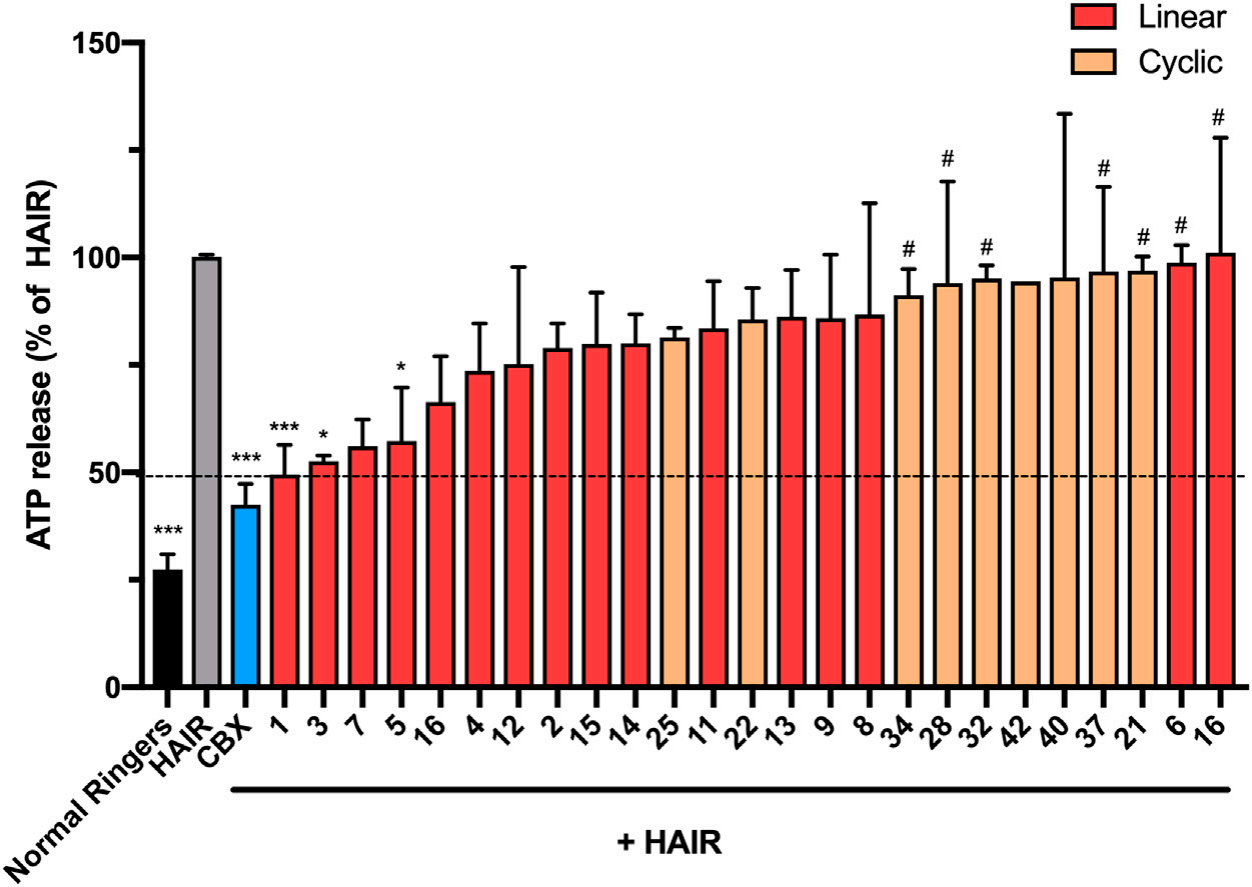
*N*-terminal, C-terminal, and cyclic modifications affect peptide5 (1) function against ischemic injury–induced ATP release from hCMVEC cells *in vitro*. *N*-terminal modified peptides **3** and **5** displayed efficacy equaling peptide5 (*p < 0.05, ***p < 0.001, respectively). N-terminal modified peptide **6** and the d-arginine–containing peptide **10** equaled the ATP release of the HAIR control indicating a complete lack of activity (^**#**^ *p* < 0.05). None of the cyclic peptides led to a reduction in ATP release that was significantly different from HAIR control, and a number of peptides (**34**, **28**, **32**, and **37**) demonstrated a level of ATP release significantly greater than peptide5, indicating loss of function of these peptides, ^**#**^ *p* < 0.05. All values represent the mean, SEM. One-way ANOVA, Dunnett’s comparison test against HAIR (*) or peptide5 (#).

To test the effect of the peptide modifications on peptide5 (**1**) activity against hemichannels, a model of ischemic insult on a cell culture model of the brain microvasculature was used to induce Cx43 hemichannel opening and ATP release (Supplementary Material, Method 4). The cells were incubated in hypoxic-acidic ion-shifted ringers (HAIR) solution to generate disrupted pH and ion balance leading to ATP leak from the cells, which can be sampled and quantified luminometrically using a commercially available firefly luciferase assay (Molecular Probes). Consistent with our previous study, treatment with HAIR solution induced an approximately three-fold increase of ATP in the culture medium. The peptide5 (**1**) treatment caused a reduction of ATP release over untreated HAIR controls (49.4 ± 6.9%; *p* = <0.001. One-way ANOVA, Dunnet’s multiple comparisons post-hoc test) as did carbenoxolone (Cbx; 42.5 ± 3.4%; *p* = <0.001), an inhibitor of both Cx43 hemichannels and gap junctions (Figure 11).

Of the 15 linear analogues (**2**–**16**) of peptide5 (**1**) tested, the majority of these modifications did not reduce the release of ATP to comparable levels as peptide5 (**1**) (Figure 11) although generally, any modification to the native peptide5 (**1**) resulted in reduced or abolished activity. The addition of either an isobutyloxycarbonyl (**3**) or butanoyl (**5**) group lead to a statistically significant reduction in ATP release (52.6 ± 1.3%; *p* < 0.05 and 57.3 ± 12.4%; *p* < 0.05, respectively), equaling the efficacy of peptide5 (**1**) (Figure 11). Peptide **7** also appeared to reduce ATP release to a similar level as Peptide5 (**1**), but due to limited peptide, this could only be used for two experimental repeats and was not found to be statistically significant. Other *N*-terminal modifications involving the addition of an ethyloxycarbonyl (**2**), benzoyl (**4**), or masking of the amine as an azide (**6**) did not reduce ATP release compared to HAIR control, and ATP release from cells treated with modified peptide **6** was significantly increased compared to peptide5 (**1**). C-terminal modifications involving the removal of the carbonyl group (**15**) or using a thioester (**16**) also did not reduce ATP release. The addition of a C-terminal amide group (peptides **11–14**) in conjunction with the *N*-terminal modifications used for peptides **2–5** did not reduce ATP release compared to HAIR, demonstrating the addition of a C-terminal amide group prevents the reduction in ATP release observed with peptides **3** and **5**. Substitution of the lysine and arginine amino acids with an *N*-methyl-lysine (**7**), *N*-methyl-arginine (**8**), d-lysine (**9**), or d-arginine (**10**) did not reduce ATP release compared to HAIR-treated cells, and ATP release from cells treated with modified peptide **10** was significantly increased compared to peptide5 (**1**). None of the cyclic variants reduced the ATP levels (Figure 11). Rather, several of these showed a complete lack of activity compared to peptide5 (**28, 32, 34,** and **37**; *p* < 0.05). However, a number of the peptides (**28**, **32**, **34**, and **37**) showed significantly increased ATP release compared to peptide5 (**1**). This indicated that cyclization of peptide5 critically hinders the hemichannel blocking activity.

**FIGURE 12:**
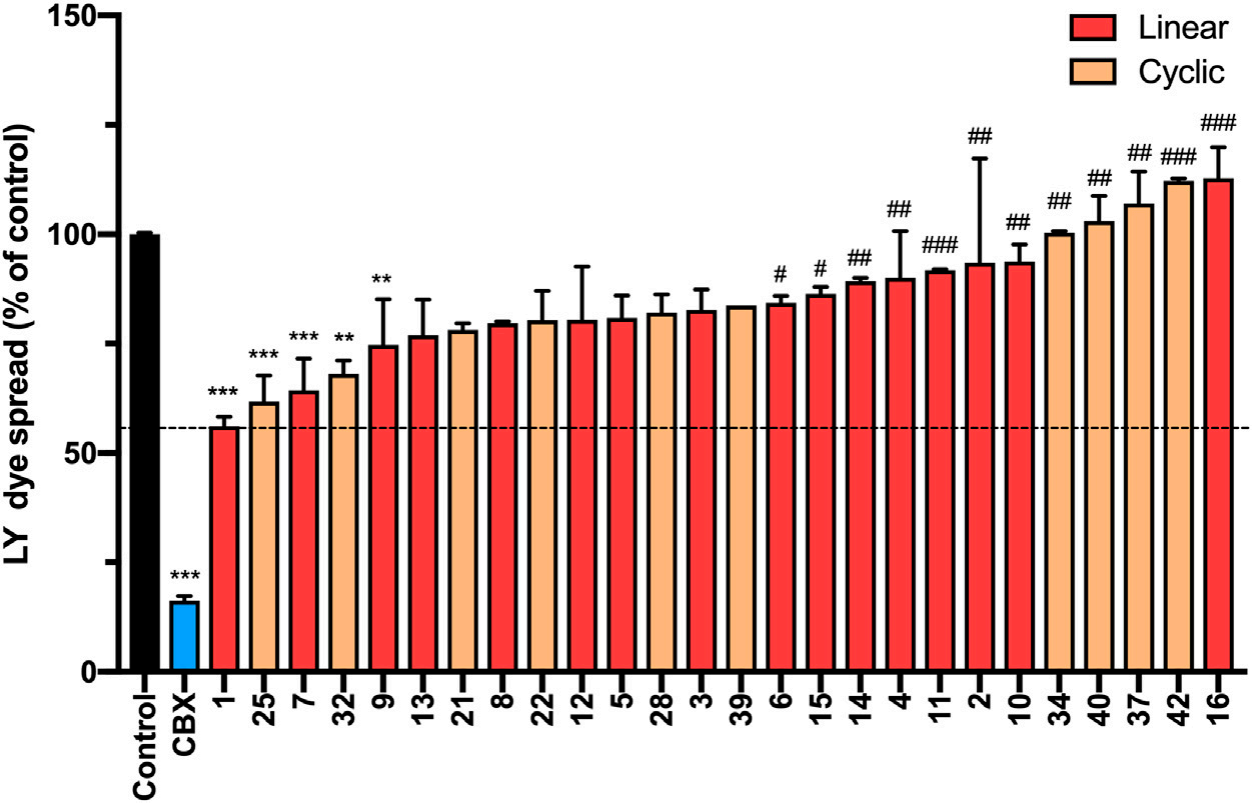
*N*-terminal, C-terminal, and cyclic modifications affect peptide5 (1) function against gap junctional coupling in hCMVEC cells *in vitro*. Positive LY dye transfer between coupled gap junction channels is represented as a percentage of the untreated control. LY dye transfer was significantly reduced compared to untreated control following treatment with Cbx or peptide5 (**1**) (****p < 0.0001). The peptides substituted with an *N*-methyl lysine (**7**) or d-lysine (**9**) prevented the spread of LY to a level comparable to peptide5 (1) (**p < 0.01, ***p < 0.001, respectively). A number of modified peptides demonstrated a level of LY dye transfer significantly greater than peptide5 and not different from control, indicating complete loss of function of these peptides, ^**#**^ *p* < 0.05. The cyclic peptides **25** and **32** significantly reduced LY transfer compared to control but not peptide5 **p < 0.01, ***p < 0.001. The cyclic peptides **40**, **34**, and **42** demonstrated a level of LY dye transfer significantly greater than peptide5 and not different from control, indicating loss of function of these peptides, ^**###**^ *p* < 0.001, ^**####**^ *p* < 0.0001. All values represent the mean, SEM. One-way ANOVA, Dunnett’s comparison test against control (*) or peptide5 (#).

We next investigated the ability of peptide5 variants to inhibit gap junctional communication using a scrape loading assay. In this assay, the cells in culture are loaded with a dye (Lucifer Yellow dilithium salt (LY), M.W. 457.25 Da) of molecular size (<1 kDa) that is able to transit through the gap junctions and the degree of dye spread is then measured by counting the number of cells that contain LY (Supplementary Material, Method 4) ^1^. Consistent with our previous study, peptide5 (**1**) and Cbx reduced the degree of dye spread following incubation with LY (56.16 ± 2.1%; *p* < 0.0001 and 16.2 ± 1.1%; *p* < 0.0001, respectively) compared to the untreated controls (Figure 12). Of the 15 linear analogues (**2**–**16**) of peptide5 (**1**) tested, two reduced LY dye spread. These were peptide **7**, containing an *N*-methyl lysine substitution, and **9**, containing a d-lysine substitution which caused a statistically significant reduction in the number of LY-positive cells (64.3 ± 7.2%; *p* < 0.001 and 74.7 ± 10.4%; *p* < 0.01, respectively), and these were not statistically different from peptide5. The corresponding peptides with *N*-methyl arginine (**8**) and d-arginine (**10**) did not reduce the degree of dye spread compared to control, and dye spread in cells treated with modified peptide **10** was significantly increased compared to peptide5 (**1**). A number of additional peptides including those with *N*-modifications (**2**, **4**, and **6**), C-modification (**15**), and a C-terminal amide group in conjunction with the *N*-terminal modifications (**11** and **14**) also showed significantly increased dye spread relative to peptide5 (**1**).

Of the 8 cyclic peptides tested, peptides **25** and **32** caused a statistically significant reduction in the number of LY-positive cells (61.7 ± 5.9%; *p* < 0.001 and 68.0 ± 3.0%; *p* < 0.01, respectively) compared to control that was not statistically different from peptide5 (**1**) (Figure 12), while peptides **34** and **42** had levels of dye spread that were significantly greater than that observed with peptide5 (**1**).

### 2.3 Serum Stability of Selected Analogs

**FIGURE 13:**
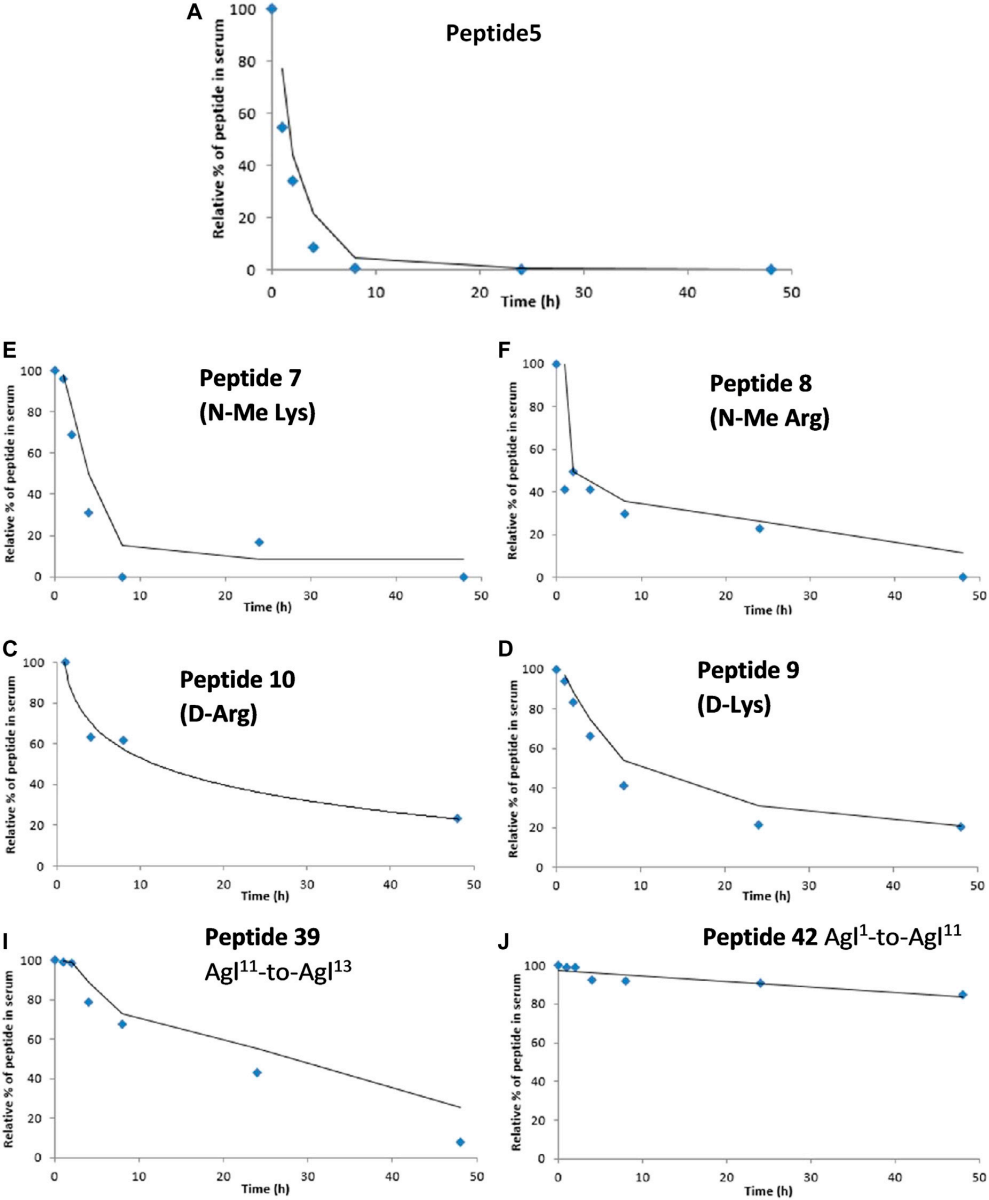
Degradation studies of peptide5 and selected analogues in 25% human serum at 37°C via monitoring by HPLC at 210 nm.

One of the limitations of using peptide5 (**1**) as a therapeutic is its poor half-life. The half-lives of peptide therapeutics can be significantly improved by *N*- and C-terminal functional group masking, cyclization, and conversion of susceptible amide bonds using D- or *N*-methylated amino acids, and these methods have been successfully used to progress peptides to the clinic (Yao et al., 2018; Khatri et al., 2019). Our previous studies of lipidated peptide5 demonstrated that the native sequence was completely degraded in rat serum in less than 5 min (Yang et al., 2020). In the present work, we instead employed the more relevant human serum and established that peptide5 (**1**) was relatively stable and degraded completely within 10 h. We then evaluated the *N*-methylated variants **7** and **8**, D amino acid variants **9** and **10**, and the two carbo cyclic peptides **39** and **42**. Each peptide was incubated with 25% aq. human serum at 37°C, and 6 aliquots were extracted within 48 h. The stability of each peptide upon exposure to human serum was then analyzed via analytical RP HPLC, and the amount of peptide still present at each time point was recorded relative to t = 0 h. The results are presented in Figure 13.

*N*-methylated peptides, **7** and **8**, showed a similar degradation profile to peptide5 (**1**), although the rate of degradation was slower. Peptide5 (**1**) containing either a D-Arg or D-Lys showed good stability with *ca.* 30% remaining after 48 h suggesting that Ser-L-Arg and Glu-L-Lys sites were susceptible to enzymatic cleavage and that replacement with the D amino acids conferred improved stability. The side-chain-to-tail carba peptide5 analogue **39** exhibited good stability over 48 h, but the head-to-tail analogue **42**, was extremely resistant to enzymatic degradation, and *ca.* 90% of the peptide remained intact at the completion of the experiment.

### 2.4 Discussion

Our goal was to apply a peptidomimetic strategy to improve the half-life of connexin channel inhibitor peptide5 (**1**), whist maintaining its ability to bind to connexin hemichannels and to moderate gap junctions. In the hemichannel assay, *N*-lipidated peptide5 analogues with short alkyl lipids (**3** and **5**) showed the comparable ability of peptide5 to block ATP release whilst an *N*-terminal azido (**6**) or D-Arg (**10**) variants were both poor at blocking hemichannels. This suggests that only certain *N*-terminal modifications are tolerated. Thus, whereas a short *N*-terminal lipid may interact with the lipid membrane to anchor peptide5 (**1**), the conversion of the amino group to an azide was detrimental, suggesting that the *N*-terminal amino group is important. At physiological pH, the amine group is protonated; when acylated or present as an azide, the positive charge is lost which may be compensated by the lipid at the *N*-terminus that can bind to the lipid membrane. The D-Arg substitution (**10**) also resulted in poor activity in the hemichannel assay. Given that alanine scan data showed that L-Arg could be substituted by L-Ala, this would suggest a stereochemical requirement as well as an interaction of the guanidyl group present in Arg for effective peptide5 (**1**) binding. Of the cyclic peptides prepared, none were able to reduce ATP lost to a level seen with the Cbx control or with peptide5 (**1**). While this may be logical given that we have shown that alanine substitutions at Asp2, Cys3, Leu5, Arg7, Pro8, and Lys11 were not tolerated in this assay, the head-to-tail cyclic amide analogues **21** and **22** and the disulfide cyclic variant **37** retain all side-chain functionality. This suggests that a less rigid linearized sequence is optimal for binding to Cx43.

In the gap junction assay, both an *N*-MeLys (**7**) and a D-Lys (**9**) substitution resulted in gap junction uncoupling comparable to that of peptide5 (**1**), and both exhibited improved human serum stability relative to peptide5. It should be noted that the *N*-Me-Lys congener (**7**) also appeared effective in the hemichannel assay and was the next best analogue after the *N*-lipidated analogues **3** and **5**. The incorporation of a D-Arg resulted in poor activity in line with the hemichannel data, thus confirming that an L-Arg is critical for both hemichannel blocking and gap junction uncoupling.

Examination of the cyclic peptides to inhibit the gap junctions revealed that side-chain Asp4-Lys11, side-chain–cyclized peptide **25**, and Cys3-Val1 cyclic peptide **32** were not as effective as peptide5 (**1**) but were better than most other cyclic peptides. As detailed in our alanine scan data, all positions in peptide5 (**1**) were able to be modified without adversely affecting gap junction blocking. However, given that the other cyclic compounds were poor, this suggests that positioning and composition of the cycle are important. The disulfide-containing cyclic compound **37** which retains all the side-chain amino acid functionality as well as the amino and carboxylate groups at the *N*- and C-termini, respectively, was a poor gap junction inhibitor as was the all-carba analogue **42**. Both **42** and **37** contain significantly larger macrocyclic rings compared to **25** and **32**, indicative of a preference for a smaller ring size to be optimal for cyclic variants of peptide5 (**1**). Nonetheless, the results demonstrated herein establish that cyclic variants of peptide5 (**1**) are able to retain some gap junction activity which was not the case for hemichannel blocking.

The goal of increasing the half-life of peptide5 whilst retaining activity has been met. The simple substitution of L- and D-Arg with D- or *N*-Me amino acids overall improved their stability. The half-life of peptide5 is estimated from Figure 13 to be *ca.* 2–3 h; its D-Arg congener was 5-fold higher with 50% remaining after 14–15 h, and the D-Lys peptide, 4-fold higher than peptide5 with 50% remaining after 12–13 h. Whilst not as noticeable, the *N*-Me-Lys variant also showed a modest increase in stability relative to peptide5. The cyclic all-carba analogues **39** and **42** were the most stable in human serum. These peptides are the most chemically modified with a 4-atom carbon bridge linking the peptide backbone with an additional rigidifying double bond. While this improved their stability, this was at the expense of both hemichannel and gap junction activity. There may therefore be scope to examine other stapling methodologies that include less rigid structures.

## 3 Conclusion

The abovementioned data have highlighted that a linearized peptide5 is the key design principle to ensure dual hemichannel and connexin activity. In the linear peptide5 analogues prepared, only those that had a small modification at the *N*-terminus, namely, an isobutyloxycarbonyl or butanoyl functional group, exhibited similar activity to peptide5 in the hemichannel assay. The only other exception was an *N*-MeLys replacement for the canonical L-Lys; remarkably, a D-Arg substitution for L-Arg abolished hemichannel activity completely, corroborating the sensitive relationship between individual amino acid substitutions and loss of activity. Converting the linear sequence to cyclic analogues containing several different cyclization modes was not tolerated in the hemichannel assay although an Asp-Lys side-chain–cyclized peptide performed well in the gap junction assay. Further work focusing on the development of peptide-based mimetics of peptide5 (**1**) should thus concentrate on the modification of the linear peptide5 sequences containing an *N*-acylated terminus or an *N*-alkylated Lys rather than cyclic variants.

## Data Availability

The original contributions presented in the study are included in the article/Supplementary Material; further inquiries can be directed to the corresponding authors.
