# Supplementary material for: Synthesis and Biological Evaluation of Termini-Modified and Cyclic Variants of the Connexin43 Inhibitor Peptide5
Source: Front Chem. 2022 Sep 13;10:877618. doi: 10.3389/fchem.2022.877618 (PMC9513234; doi:10.3389/fchem.2022.877618)
Supplement: Supplementary file 1 [file DataSheet1.docx]

Supplementary Material

# General information for peptide synthesis

All reagents were purchased as reagent grade and used without further purification. Solid phase peptide synthesis was carried out manually in a glass-fritted vessel at room temperature or in an automated peptide synthesizer as indicated. Solvents for peptide synthesis and reverse phase high-pressure liquid chromatography (RP-HPLC) were purchased as synthesis grade and HPLC grade, respectively.

**Amino acids:** All Fmoc amino acids were purchased from GL Biochem (Shanghai, China) with the following side chain protection: Fmoc-Arg(Pbf)-OH (Pbf = 2,2,4.6,7-pentamethyldihydrobenzofuran-5-sulfonyl), Fmoc-D-Arg(Pbf)-OH, Fmoc-Asp(tBu)-OH (tBu = *tert*-butyl), Fmoc-Cys(Trt)-OH (Trt = triphenylmethyl), Fmoc-Glu(tBu)-OH, Fmoc-Lys(Boc)-OH (Boc = *tert*-butyloxycarbonyl) Fmoc-D-Lys(Boc)-OH, Fmoc-Ser(tBu)-OH, Fmoc-Thr(tBu)-OH. Boc-amino acids were purchased from Polypeptides (Strasbourg, France) with the following side chain protection: Boc-Arg(Tos)-OH (Tos = *p*-toluenesulfonyl), Boc-Asp(cHex)-OH (cHex = cyclohexyl), Boc-Cys(4-MeBn)-OH (Bn = benzyl), Boc-Glu(cHex)-OH, Boc-Lys(2-Cl-Z)-OH (Z = benzyloxycarbonyl), Boc-Ser(Bn)-OH, Boc-Thr(Bn)-OH. Fmoc-Lys(Mtt)-OH (Mtt = 4-methyltrityl) and Fmoc-Asp(*O*-2-Ph^i^Pr)-OH were obtained from Merck (Darmstadt, Germany), Fmoc-3,3-dimethyl-L-Cys(Trt) (Penicillamine) was purchased from ChemImpex (Wood Dale IL) , Fmoc-allylglycine (Agl) was from AK Scientific (Union City, CA).

**Resins and Linkers:** 4-[(R,S)-α-[1-(9H-fluoren-9-yl)-methoxyformamido]-2,4-dimethoxybenzylphenoxyacetic (Rink linker) and TrtSCH_2_CH_2_CO_2_H was purchased from GL Biochem, Fmoc-Thr(*t*Bu)-HMPP-OH (HMPP = hydroxymethylphenylpropionic acid), Boc-Ala-PAM (PAM = 4-hydroxymethylphenylacetic acid) and Boc-Gly PAM was obtained from Polypeptides, 4-(4-hydroxymethyl-3-methoxyphenoxy)butyric acid (HMPB) linker was obtained from Iris Biotech, aminomethyl polystyrene resin (AM-PS) was purchased from Rapp Polymere (Tubingen, Germany)

**Coupling reagents:** *O*-(6-Chlorobenzotriazol-1-yl)-*N,N,N’,N’*-tetramethyluronium hexafluoroborate (HCTU), *O*- (benzotriazol-1-yl)-*N,N,N′,N′*-tetramethyluronium hexafluorophosphate (HBTU) were purchased from GL Biochem, diisopropylcarbodimide (DIC) and DMAP were obtained from AK Scientific, benzotriazol-1-yloxytripyrrolidinophosphonium hexafluorophosphate (PyBOP), benzotriazol-1-yloxy)tris(dimethylamino)-phosphonium hexafluorophosphate (BOP), and Cl-HOBt were obtained from Aapptec ( Louisville, KY)

**Miscellaneous reagents:** Dichloromethane (CH_2_Cl_2_), and diethyl ether (Et_2_O) were purchased from ECP (Auckland, New Zealand). Trifluoroacetic acid (TFA) was purchased form Oakwood Chemicals (Estill, NC, USA). *N,N*-Diisopropylethylamine (DIPEA), collidine, piperidine, triisopropylsilane (TIPS), 3,6-dioxa-1,8-octanedithiol (DODT), dimethyl sulphate, 4-nitrobenzenesulfonyl chloride (*o*-NBS-Cl), 2-mercaptoethanol, 4,4'-dithiodipyridine (DTDP), 5,5'-dithiobis-(2-nitrobenzoic acid) (DTNB), Hoveyda-Grubbs’ second generation were purchased from Sigma-Aldrich (St. Louis, MO, USA). Acetonitrile (CH_3_CN) (HPLC grade), *N,N*-dimethylformamide (DMF) (synthesis grade) were purchased from Thermo Fisher Scientific (Hampton, NH, USA). H_2_O was purified using a Sartorius (Göttingen, Germany) arium® pro ultrapure water system. Grubbs, N-Me reagents,

**HPLC and MS:** Analytical RP-HPLC was performed on or Dionex (Sunnyvale, CA, USA) UltiMate 3000 system using a Phenomenex (Torrance, CA, USA) Gemini C18 column (5 μm, 110 Å, 4.6 × 150 mm). Buffer A: 0.1% (*v/v*) TFA in H_2_O; buffer B: 0.1% (*v/v*) TFA in CH_3_CN. Analytical liquid chromatography-mass spectrometry (LCMS) was performed on an Agilent (Santa Clara, CA, USA) 1260 Infinity equipped with an Agilent 6120 Quadrupole LC-MS using an Agilent Zorbax 300SB-C3 column (3.5 μm, 3.0 x 150 mm) with a linear gradient of 5-95% B (ca. 3% B/min) and a flow rate of 0.3 mL min^-1^. Buffer A: 0.1% (*v/v*) formic acid in H_2_O; buffer B: 0.1% (*v/v*) formic acid in CH_3_CN. Crude peptides were purified on a Dionex UltiMate 3000 preparative system using an appropriate HPLC column and gradient depending on the analytical HPLC profile. A buffer consisting of A: 0.1% (*v/v*) TFA in water and B 0.1% (*v/v*) TFA in CH_3_CN was used and fractions were analysed using either HPLC or ESI-MS, pooled and recovered by lyophilization.

# Synthesis of Linear Peptides

***Peptides 2-5:***

To aminomethyl polystyrene resin (100 mg, 0.1 mmol, loading: 0.98 mmol/g) was added Fmoc-Thr(*t*Bu)-HMPP-OH (115 mg, 2.0 equiv., 0.2 mmol) in DCM (2 mL) followed by addition of DIC (31 µL, 2.0 equiv., 0.2 mmol). The reaction mixture was gently agitated at room temperature for 90 mins, the solution was removed by filtration and washed with DMF and then methanol. A negative Kaiser test confirmed complete acylation which afforded 174 mg of resin. Iterative Fmoc SPPS was then undertake with a Tribute automated synthesizer consisting of an Fmoc deprotection with 20% piperdine in DMF (3 mL) for 2 x 5 mins, a DMF wash (3 ml) 6 x 30 seconds, a coupling using Fmoc-Xaa (4 equiv.), HCTU in DMF (3.8 equiv.) and DIPEA in NMP (10 equiv.) and finally a DMF wash (3 ml) 6 x 30 seconds. At the completion of the sequence a final Fmoc deprotection was undertaken, the resin was washed with DMF and then methanol and dried. This afforded 370 mg of resin which was split into 4 equal portions (*ca.* 90 mg, 0.05 mmol).

***Peptide 2:*** The resin (90 mg, 0.05 mmol) was swelled in DCM for 10 mins, drained, DCM (1 ml) was added and ethyl chloroformate (48 µL, 20 equiv.) and NMM (55 µL, 20 equiv.) were added. After 15 mins a negative Kaiser test was observed thus the solution was removed under vacuum, washed with DMF and DCM and dried. Cleavage using 94% TFA, 1% TIS, 2.5% DODT and 2.5% water (5 mL, v/v/v/v) for 2.5 h was followed by precipitation with cold diethyl ether and recovery by centrifugation to afford crude **2** (63 mg) as a white powder. Purification by RP-HPLC (Xterra, C18, 10 µ, 19 x 300 mm) using a gradient of 5%B to 65% B over 65 mins at 10 mL/min afforded **2** (9.74 mg, 26% yield).

***Peptide 3:*** The resin (90 mg, 0.05 mmol) was swelled in DCM for 10 mins, drained, DCM (1 ml) was added and isobutyl chloroformate (65 µL, 20 equiv.) and NMM (55 µL, 20 equiv.) were added. After 15 mins a negative Kaiser test was observed thus the solution was removed under vacuum, washed with DMF and DCM and dried. Cleavage using 94% TFA, 1% TIS, 2.5% DODT and 2.5% water (5 mL, v/v/v/v) for 2.5 h was followed by precipitation with cold diethyl ether and recovery by centrifugation to afford crude **3** (69 mg) as a white powder. Purification by RP-HPLC (Xterra, C18, 10 µ, 19 x 300 mm) using a gradient of 5%B to 65% B over 65 mins at 10 mL/min afforded **3** (14.0 mg, 49% yield).

***Peptide 4:*** The resin (90 mg, 0.05 mmol) was swelled in DCM for 10 mins, drained, DCM (1 ml) was added benzoyl chloride (58 µL, 20 equiv.) and NMM (55 µL, 20 equiv.) were added. After 15 mins a negative Kaiser test was observed thus the solution was removed under vacuum, washed with DMF and DCM and dried. Cleavage using 94% TFA, 1% TIS, 2.5% DODT and 2.5% water (5 mL, v/v/v/v) for 2.5 h was followed by precipitation with cold diethyl ether and recovery by centrifugation to afford crude **3** (72 mg) as a white powder. Purification by RP-HPLC (Xterra, C18, 10 µ, 19 x 300 mm) using a gradient of 5%B to 65% B over 65 mins at 10 mL/min afforded **4** (15.2 mg, 41% yield).

***Peptide 5:*** The resin (90 mg, 0.05 mmol) was swelled in DCM for 10 mins, drained, DCM (1 ml) was added butyric acid (39 µL, 20 equiv.) DIC (78 µL, 20 equiv.) and 1-2 crystals of DMAP were added. After 45 mins a negative Kaiser test was observed thus the solution was removed under vacuum, washed with DMF and DCM and dried. Cleavage using 94% TFA, 1% TIS, 2.5% DODT and 2.5% water (5 mL, v/v/v/v) for 2.5 h was followed by precipitation with cold diethyl ether and recovery by centrifugation to afford crude **3** (56 mg) as a white powder. Purification by RP-HPLC (Xterra, C18, 10 µ, 19 x 300 mm) using a gradient of 5%B to 65% B over 65 mins at 10 mL/min afforded **4** (11 mg, 44% yield).

***Peptide 6:***

The linear sequence was assembled as described for peptides **2-5** on a 0.1 mmol scale to afford 320 mg of resin. 160 mg (0.05 mmol) was taken, swollen in DMF followed by the addition of imidazole-1-sulfonyl azide.HCl (74 mg, 7 equiv.) dissolved in DMF (2 mL). The resin was shaken for 2 h after which time a positive Kaiser test indicated incomplete reaction therefor the solution was removed and replaced with fresh reagents and shaking was continued overnight. A negative Kaiser test was observed thus the solution was removed under vacuum, washed with DMF and DCM and dried. Cleavage using 94% TFA, 1.5 % TIS and 2.5% water (5 mL, v/v/v/v) for 2.5 h was followed by precipitation with cold diethyl ether and recovery by centrifugation to afford crude **6** (46 mg) as a white powder. Purification by RP-HPLC (Xterra C18, 10 µ, 19 x 300 mm) using a gradient of 1%B to 61% B over 60 mins at 10 mL/min afforded **6** (12.7 mg, 18% yield).

***Peptide 7:***

The linear sequence was assembled using the procedures for peptides 2-5 up to Lys-11 to afford NH_2_-Lys(Boc)-Thr(tBu)-HMPP-AM resin. A mixture of *o-*NBS-Cl (88.6 mg, 0.4 mmol) in NMP (2 mL) and *sym*-collidine (132 µL, 1.0 mmol) was added to the resin, which was shaken for 15 min at r.t. and a fresh mixture of *o-* NBS-Cl (88.6 mg, 0.4 mmol) in NMP (2 mL) and *sym*-collidine (132 µL, 1.0 mmol) was added to the resin and shaken for 10 min at r.t. The resin was drained and washed with NMP (5 x 2 mL). DBU (45 µL, 0.3 mmol) in NMP (1 mL) was added and then shaken vigorously for 3 min at r.t. The resin was drained and washed with NMP. DMS (73 µL, 1.0 mmol) in NMP (1 mL) was added and then shaken for 2 min at r.t. and a fresh mixture of DMS (73 µL, 1.0 mmol) in NMP (1 mL) was added to resin and shaken for 2 min at r.t. The resin was drained and washed with NMP (5 x 2 mL). A mixture of 2-mercaptoethanol (70 µL, 1.0 mmol), DBU (74.7 µL, 0.5 mmol) and NMP (2 mL) was added to the resin, which was shaken for 5 min at r.t. and a fresh mixture of 2-mercaptoethanol (70 µL, 1.0 mmol), DBU (74.7 µL, 0.5 mmol) and NMP (2 mL) was added to the resin for 5 min. The resin was drained and washed with NMP. The resin was returned to the Tribute automated peptide synthesiser and peptide chain elongation was completed as described for peptides 2-5. The resin was washed thoroughly with DMF and CH_2_Cl_2_, and air dried. A cleavage cocktail of 94% TFA, 2.5% H_2_O, 2,5% DODT and 1% TIS (v/v/v/v, 5 mL) was added to the dry resin and the mixture was shaken for 2 h which was followed by precipitation with cold diethyl ether and recovery by centrifugation to afford crude **7.** The crude product was purified by semi-preparative RP-HPLC on a Phenomenex Gemini C18 column (5 µ, 110 Å, 10.0 x 250 mm) running a gradient of 5-24% (0.5% MeCN per minute) to afford **7** as a white amorphous solid (66.2 mg, 47%)

***Peptide 8:***

The linear sequence was assembled using the procedures for peptides **2-5** up to Lys-11 to afford NH_2_-Arg(Pbf)-Pro-Thr(tBu)-Glu(tBu)-Lys(Boc)-Thr(tBu)-HMPP-AM resin. The identical N-methylation and subsequent elongation and cleavage procedures used for peptide **7** were then followed to afford **8** as a white amorphous solid (43.2 mg, 31%)

***Peptide 9:***

The linear sequence was assembled on Fmoc-Thr(tBu)-HMPP-AM resin (0.1 mmol) and elongated as described for peptides **2-5** except that Fmoc-D-Arg(Pbf) was used for Arg-7. Purification by RP-HPLC (Gemini C18 column 5 µ, 110 Å, 10.0 x 250 mm) using a gradient of 5-20% (0.25% MeCN per minute) gave **14** as a white amorphous solid (36.3 mg, 26%)

***Peptide 10:***

The linear sequence was assembled on Fmoc-Thr(tBu)-HMPP-AM resin (0.1 mmol) and elongated as described for peptides **2-5** except that Fmoc-D-Lys(Boc) was used for Lys-11. Purification by RP-HPLC (Gemini C18 column 5 µ, 110 Å, 10.0 x 250 mm) using a gradient of 5-20% (0.25% MeCN per minute) gave **14** as a white amorphous solid (22.3 mg, 16%)

***Peptides 11-14:***

To aminomethyl polystyrene resin (100 mg, 0.1 mmol, loading: 0.98 mmol/g) was added a solution of Rink amide linker (216 mg, 4 equiv., 0.4 mmol), Cl-HOBT (68 mg, 4 equiv., 0.4 mmol) and DIC (62 µL, 4 equiv., 0.4 mmol) in DMF (2 mL). The reaction mixture was gently agitated at room temperature for 90 mins, the solution was removed by filtration and washed with DMF and then methanol. A negative Kaiser test confirmed complete acylation which afforded 174 mg of resin. Iterative Fmoc SPPS was then undertake with a Tribute automated synthesizer consisting of an Fmoc deprotection with 20% piperdine in DMF (3 mL) for 2 x 5 mins, a DMF wash (3 ml) 6 x 30 seconds, a coupling using Fmoc-Xaa (4 equiv.), HCTU in DMF (3.8 equiv.) and DIPEA in NMP (10 equiv.) and finally a DMF wash (3 ml) 6 x 30 seconds. At the completion of the sequence a final Fmoc deprotection was undertaken, the resin was washed with DMF and then methanol and dried. This afforded 306 mg of resin which was split into 4 equal portions (*ca.* 75 mg, 0.05 mmol).

***Peptide 11:*** The resin (75 mg, 0.05 mmol) was swelled in DCM for 10 mins, drained, DCM (1 ml) was added and isobutyl chloroformate (65 µL, 20 equiv.) and NMM (55 µL, 20 equiv.) were added. After 15 mins a negative Kaiser test was observed thus the solution was removed under vacuum, washed with DMF and DCM and dried. Cleavage using 94% TFA, 1% TIS, 2.5% DODT and 2.5% water (5 mL, v/v/v/v) for 2.5 h was followed by precipitation with cold diethyl ether and recovery by centrifugation to afford crude **11** (53 mg) as a white powder. Purification by RP-HPLC (Xterra, C18, 10 µ, 19 x 300 mm) using a gradient of 5%B to 65% B over 65 mins at 10 mL/min afforded **11** (15.6 mg, 43% yield).

***Peptide 12:*** The resin (75 mg, 0.05 mmol) was swelled in DCM for 10 mins, drained, DCM (1 ml) was added and isobutyl chloroformate (65 µL, 20 equiv.) and NMM (55 µL, 20 equiv.) were added. After 15 mins a negative Kaiser test was observed thus the solution was removed under vacuum, washed with DMF and DCM and dried. Cleavage using 94% TFA, 1% TIS, 2.5% DODT and 2.5% water (5 mL, v/v/v/v) for 2.5 h was followed by precipitation with cold diethyl ether and recovery by centrifugation to afford crude **12** (58 mg) as a white powder. Purification by RP-HPLC (Xterra, C18, 10 µ, 19 x 300 mm) using a gradient of 5%B to 65% B over 65 mins at 10 mL/min afforded **12** (18.2 mg, 49% yield).

***Peptide 13:*** The resin (75 mg, 0.05 mmol) was swelled in DCM for 10 mins, drained, DCM (1 ml) was added benzoyl chloride (58 µL, 20 equiv.) and NMM (55 µL, 20 equiv.) were added. After 15 mins a negative Kaiser test was observed thus the solution was removed under vacuum, washed with DMF and DCM and dried. Cleavage using 94% TFA, 1% TIS, 2.5% DODT and 2.5% water (5 mL, v/v/v/v) for 2.5 h was followed by precipitation with cold diethyl ether and recovery by centrifugation to afford crude **13** (50 mg) as a white powder. Purification by RP-HPLC (Xterra, C18, 10 µ, 19 x 300 mm) using a gradient of 5%B to 65% B over 65 mins at 10 mL/min afforded **13** (13.9 mg, 37% yield).

***Peptide 14:*** The resin (75 mg, 0.05 mmol) was swelled in DCM for 10 mins, drained, DCM (1 ml) was added butyric acid (39 µL, 20 equiv.) DIC (78 µL, 20 equiv.) and 1-2 crystals of DMAP were added. After 45 mins a negative Kaiser test was observed thus the solution was removed under vacuum, washed with DMF and DCM and dried. Cleavage using 94% TFA, 1% TIS, 2.5% DODT and 2.5% water (5 mL, v/v/v/v) for 2.5 h was followed by precipitation with cold diethyl ether and recovery by centrifugation to afford crude **14** (56 mg) as a white powder. Purification by RP-HPLC (Xterra, C18, 10 µ, 19 x 300 mm) using a gradient of 5%B to 65% B over 65 mins at 10 mL/min afforded **14** (16 mg, 44% yield).

***Peptide 15:***

The linear sequence was assembled on O-*t*-butylthreoninol 2-chlorotrityl resin (loading = 0.5 mmol/g) as described for peptides **2-5** on a 0.1 mmol scale and cleaved using 94% TFA, 1.5 % TIS and 2.5% water (5 mL, v/v/v/v) for 2.5 h was followed by precipitation with cold diethyl ether and recovery by centrifugation. Purification by RP-HPLC (Xterra C18, 10 µ, 19 x 300 mm) using a gradient of 1%B to 61% B over 60 mins at 10 mL/min afforded **12** (3.01 mg, 2% yield).

***Peptide 16:***

To aminomethyl polystyrene resin (100 mg, 0.1 mmol, loading: 0.98 mmol/g) was added a solution of Boc-Gly-PAM (64.6 mg, 2 equiv., 0.2 mmol, DIC (31 µL, 2 equiv., 0,2 mmol) in DMF/DCM (1:9, v/v) and left to stand overnight. A negative Kaiser test resulted. The Boc group was removed with neat TFA (2-3 mL) for 2 mins, the TFA drained and the resin washed with DMF. 3-(S-tritylmercapto)propionic acid (191 mg, 0.55 mmol), and HCTU (1.31 mL of a 0.4 M solution, 0.52 mmol) were mixed followed by the addition of DIPEA (0.198 mL, 12 equiv.) and the solution was briefly mixed until dissolved (10-20 secs) and added to the resin. After agitation for 20 mins the resin was treated with 95% TFA, 2.5 % TIS and 2.5% water (v/v/v, 3 mL) in 2 min time periods until the disappearance of the yellow colour. The solution was removed and the resin washed with DMF and Boc-Thr(Bn)-OH (170 mg, 0.55 mmol), and HCTU (1.31 mL of a 0.4 M solution, 0.52 mmol) were mixed followed by the addition of DIPEA (0.198 mL, 12 equiv) and the solution was briefly mixed until dissolved (10-20 secs), added to the resin and left to stand for 1 h. The solution was removed and iterative Boc SPPS was performed consisting of a Boc deprotection using neat TFA (2 mins), a 30 second DMF wash, coupling of Boc-Xaa (5.5 equiv.) HCTU (5.3 equiv.) and DIPEA (12 equiv.) for 10 mins and a 30 second DMF wash. At the completion of the sequence a final Boc deprotection was undertaken, the resin was washed with DMF and then methanol and dried. This afforded 420 mg of resin which was cleaved using anhydrous HF/*p*-cresol (20/1, v/v) at 0 ºC for 1 h. The peptide was precipitated from cold diethyl ether, isolated by filtration dissolved in 50% aq. MeCN containing 0.1% TFA and recovered by lyophilization to afford 172 mg of crude **16**. Purification of a 30 mg batch by RP-HPLC (Xterra C18, 10 µ, 19 x 300 mm) using a gradient of 1%B to 60% B over 59 mins at 10 mL/min afforded **16** (10.9 mg).

# Synthesis of Cyclic Peptides

***Peptide 21 and 22***

To aminomethyl polystyrene resin (202 mg, 0.2 mmol, loading: 0.98 mmol/g) was added a solution of Boc-Ala-PAM (135 mg, 0.4 mmol, DIC (62 µL, 0.4 mmol) in DCM (5 mL)and left to stand overnight. A negative Kaiser test resulted. The Boc group was removed with neat TFA (2-3 mL) for 2 mins, the TFA drained and the resin washed with DMF. 3-(S-tritylmercapto)propionic acid (383 mg, 0.55 mmol), and HCTU (2.61 mL of a 0.4 M solution, 1.045 mmol) were mixed followed by the addition of DIPEA (0.4 mL, 12 equiv.) and the solution was briefly mixed until dissolved (10-20 secs) and added to the resin. After agitation for 20 mins the resin was treated with 95% TFA, 2.5 % TIS and 2.5% water (v/v/v, 5 mL) in 2 min time periods until the disappearance of the yellow colour. The solution was removed and the resin washed with DMF and Boc-Asp(OCy)-OH (346 mg, 0.55 mmol), and HCTU (2.62 mL of a 0.4 M solution, 0.52 mmol) were mixed followed by the addition of DIPEA (0.4 mL, 12 equiv.) and the solution was briefly mixed until dissolved (10-20 secs), added to the resin and left to stand for 1 h. The solution was removed and iterative Boc SPPS was performed consisting of a Boc deprotection using neat TFA (2 mins), a 30 second DMF wash, coupling of Boc-Xaa (5.5 equiv.) HCTU (5.3 equiv.) and DIPEA (12 equiv.) for 10 mins and a 30 second DMF wash. At the completion of the sequence a final Boc deprotection was undertaken, the resin was washed with DMF and then methanol and dried. This afforded 655 mg of resin which was cleaved using anhydrous HF/*p*-cresol (20/1, v/v) at 0 ºC for 1 h. The peptide was precipitated from cold diethyl ether, isolated by filtration dissolved in 50% aq. MeCN containing 0.1% TFA and recovered by lyophilization to afford 271 mg of crude alkylthioester **19** [MS (ESI+) *m/z* 778.4 (calcd. for [M+2H]^2+^, 777.9), 519.1 (calcd. for [M+3H]^3+^, 519.3].

Crude alkylthioester **19** (40 mg, 25.7 x 10^-4^ mmol) was dissolved in a buffer of 200 mM MPAA, 20 mM TCEP.HCl, 0.2 M phosphate and 6 M Gn.HCl (pH = 6.77) at a peptide concentration of 1 mM. The solution was left to stand at rt for 3 h, 0.1% aq. TFA was added (30 mL) to pH = 2 and excess MPAA was extracted twice with diethyl ether. The peptide was recovered by loading onto a semi preparative RP HPLC column (Xterra C18, 10 µ, 19 x 300 mm); after non-retaining material had finished eluting the peptides were eluted with 80% aq. MeCN and lyophilized to give 45.5 mg of crude cyclized peptides **21** and **22**. Purification by RP-HPLC (Xterra C18, 10 µ, 19 x 300 mm) using a gradient of 1% B to 20% B over 6 mins followed by 20-50%B over 60 mins at 10 mL/min gave **22** (5.36 mg) and **21** (11.27 mg).


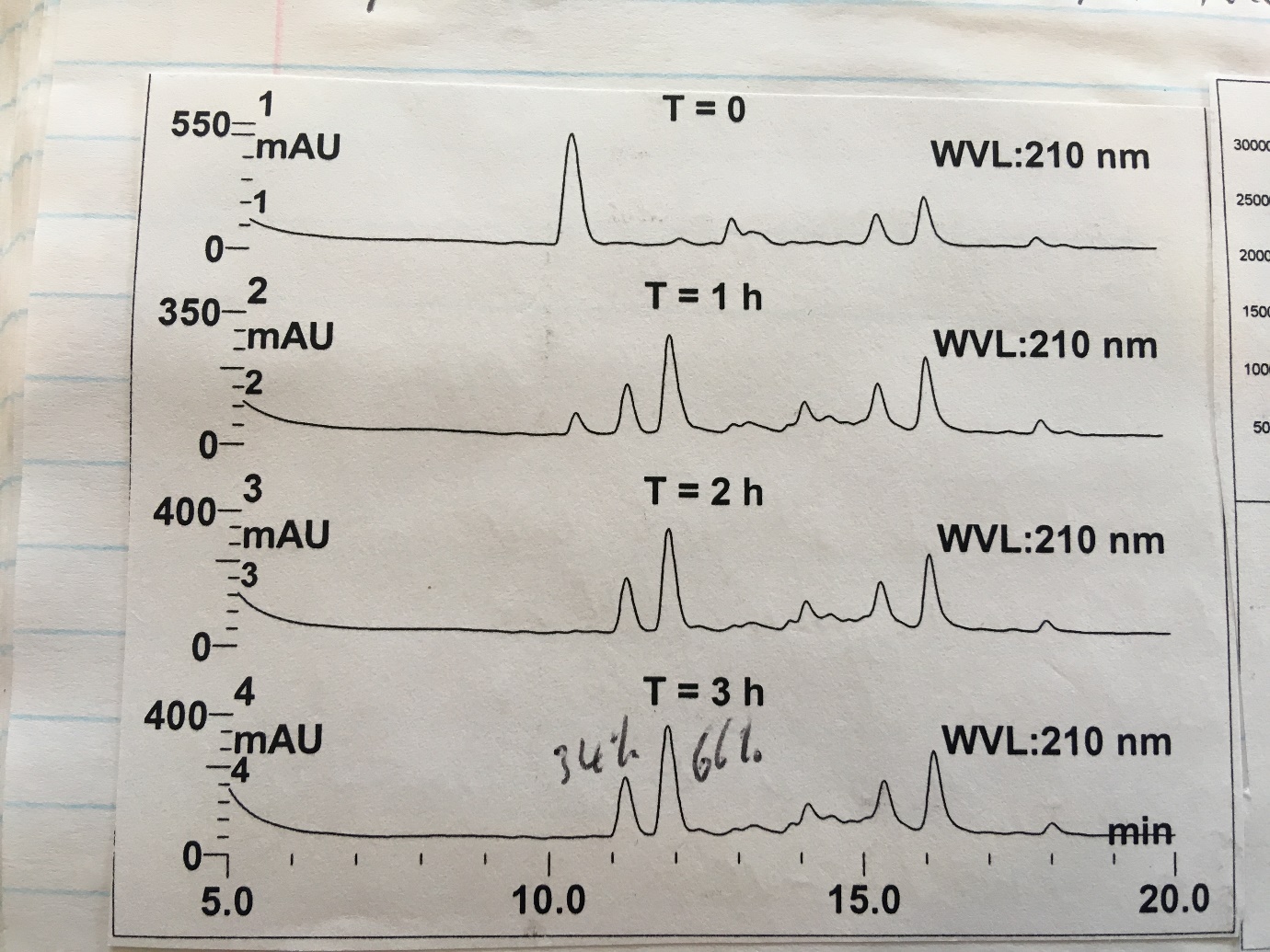


**22**

**19**

**21**

**Figure S1.** HPLC reaction monitoring (210 nm) of NCL-mediated cyclisation of Peptide5 alkylthioester **19** to from cyclic peptides **21** and **22**.

***Peptide 25***

The linear sequence was assembled on Fmoc-Thr(tBu)-HMPP-AM resin (0.1 mmol) and elongated as descried for peptides **2-5** except that Fmoc-Lys(Mtt) was used for Lys-11 and Fmoc-Asp(*O*-2-Ph^i^Pr)-OH was employed for Asp-2. The final Fmoc group was removed by treatment with 20% piperidine in DMF and washed with DMF. Boc_2_O (218 mg, 10 mmol) dissolved in DMF (2 mL) and the reaction shaken for 30 mins, washed with DMF and then MeOH. A Kaiser test confirmed complete acylation. The Mtt and 2-Ph^i^Pr group were simultaneously removed by treatment with 1% TFA in DCM (2 mL, 15 x 2 mins), followed by washing of the resin with DCM and methanol. 300 mg of resin was obtained. 200 mg of resin (0.068 mmol) was swelled in DMF and treated with BOP (300 mg, 0.68 mmol), Cl-HOBt (115 mg, 0.68 mmol) and DIPEA (236 µL, 1.36 mmol) in DMF (4 mL) for 3 h after which time a positive Kaiser test was observed. The reaction was repeated with fresh reagents for a further 3 h with little change in the Kaiser test. Cleavage using 94% TFA, 1% TIS, 2.5% DODT and 2.5% water (5 mL, v/v/v/v) for 2.5 h was followed by precipitation with cold diethyl ether and recovery by centrifugation to afford crude **25** (20 mg) as a white powder. Purification by RP-HPLC (Xterra, C18, 10 µ, 19 x 300 mm) using a gradient of 5%B to 65% B over 60 mins at 10 mL/min afforded **25** (3.83 mg, 4% yield).

***Peptide 28:***


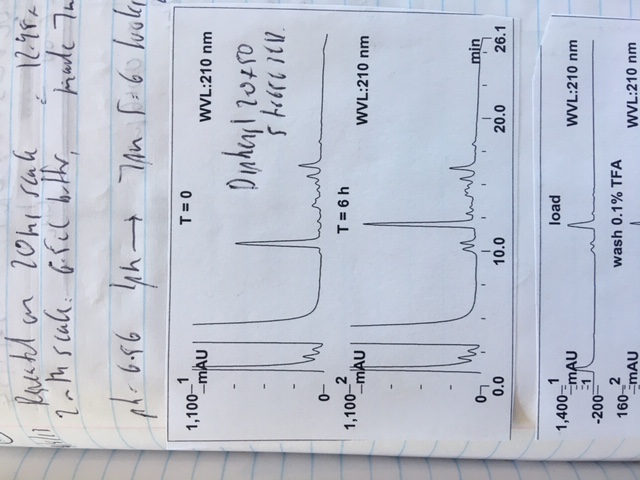

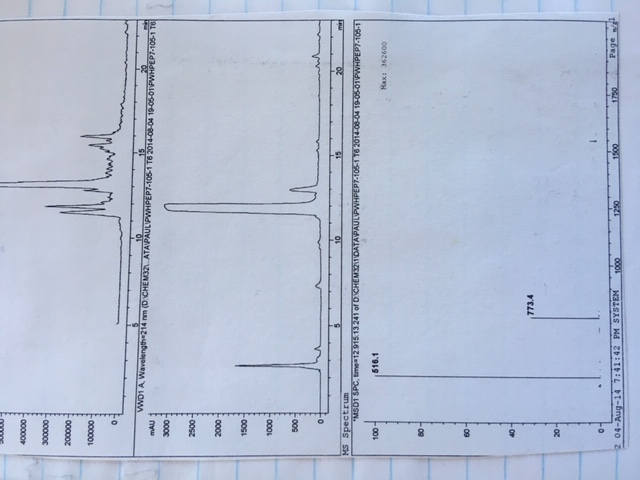
Crude thioester **14** (20 mg, 12.98 x 10^-3^ mmol) was dissolved in a buffer of 200 mM MPAA, 20 mM TCEP.HCl, 0.2 M phosphate and 6 M Gn.HCl (pH = 6.56) at a peptide concentration of 2 mM. After 6 h the reaction was adjudged complete by HPLC analysis and 0.1% aq. TFA was added (7 mL) excess MPAA was extracted twice with diethyl ether. Phenylthioester **27** was isolated by loading onto a semi preparative RP HPLC column (Xterra C18, 10 µ, 19 x 300 mm) and eluted with 1%B. After non-retaining material had finished eluting the peptide were eluted with 80% aq. MeCN and lyophilized to give 16.5 mg of crude phenylthioester. **27** (15 mg, 9.7 x 10^-3^ mmol) was dissolved in a buffer of 0.2 M phosphate and 6 M Gn.HCl (pH = 6.8) at a peptide concentration of 1 mM. After 2 h the reaction mixture was acidified by the addition of 0.1% aq. TFA and purified by RP-HPLC (Xterra, C18, 10 µ, 19 x 300 mm) using a gradient of 5%B to 65% B over 60 mins at 10 mL/min to afford **28** (2.92 mg, 18% yield).

**13**

**27**


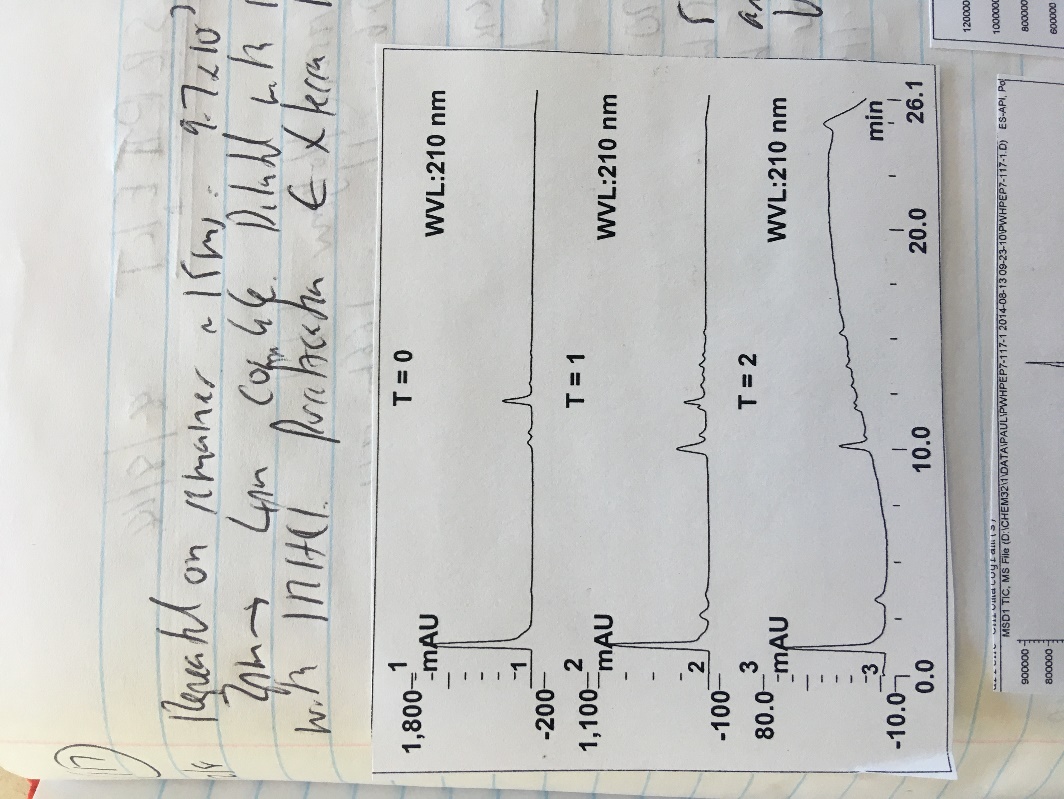
**Figure S2.** ***Left***: HPLC reaction monitoring of thiol-thioester exchange of alkylthioester **14** to phenylthioester **27.**  ***Right***: ESI-MS of phenylthioester **27,** (Calc. (M+2H)^2+^ 773.8, Found 773.4)

**Figure S3.** HPLC reaction monitoring of thiolactamisation of phenylthioester **27.**

***Peptide 32:***

The linear sequence was assembled on Fmoc-Thr(tBu)-HMPP-AM resin (0.05 mmol) and elongated as descried for peptides 2-5. At the completion of the sequence a final Fmoc deprotection was undertaken, the resin was washed with DMF and then methanol and dried. This afforded 185 mg of resin. Bromoacetic acid (56 mg, 0.4 mmol) was dissolved in DCM (1 mL) and DIC (31 µL, 0.2 mmol) was added and the mixture stood for 15 mins with a precipitate observed. The reaction was filtered through cotton wool, DCM was removed under a stream of nitrogen, the residue dissolved in DMF (2 mL) and was added to resin. After 40 mins of agitation a Kaiser test was negative. The resin was washed with DMF and methanol and dried. Cleavage using 94% TFA, 1% TIS, 2.5% DODT and 2.5% water (5 mL, v/v/v/v) for 2.5 h was followed by precipitation with cold diethyl ether and recovery by centrifugation to afford 2-bromoacetylated peptide **31** (55 mg) as a white powder [MS (ESI+) *m/z* 1517.5 (calcd. for [M+H]^+^, 1515.3), 759.2 (calcd. for [M+2H]^2+^, 759.3]. **31** (25 mg, 16.4 x 10^-3^ mmol) was dissolved in a buffer of 0.2 M phosphate and 6 M Gn.HCl (pH = 6.9) at a peptide concentration of 1 mM. A sample taken directly after the dissolution of the peptide showed no starting peptide and an earlier eluting peak. The reaction mixture was acidified by the addition of 0.1% aq. TFA and purified by RP-HPLC (Xterra, C18, 10 µ, 19 x 300 mm) using a gradient of 5%B to 65% B over 60 mins at 10 mL/min to afford **32** (14.16 mg, 59% yield).

***Peptide 34:***

The linear sequence was assembled on Fmoc-Thr(tBu)-HMPP-AM resin (0.1 mmol) and elongated as descried for peptides **2-5** except that Fmoc-Pen(Trt) was used for Val-1 The final Fmoc group was removed by treatment with 20% piperidine in DMF and washed with DMF.. At the completion of the sequence a final Fmoc deprotection was undertaken, the resin was washed with DMF and then methanol and dried. This afforded 348 mg of resin which was cleaved using 94% TFA, 1% TIS, 2.5% DODT and 2.5% water (10 mL, v/v/v/v) for 2.5 h was followed by precipitation with cold diethyl ether and recovery by centrifugation to afford **33** 140 mg [MS (ESI+) *m/z* 714.2 (calcd. for [M+2H]2^+^, 714.8), 476.5 (calcd. for [M+3H]^3+^, 476.9]. **33** (20 mg, 14.01 x 10^-3^ mmol) was added to a suspension of iodine (65 mg, 464 x 10^-3^ mmol) in HOAc/H_2_O (4:1, 4 mL) and the mixture agitated for 30 mins after which time the reaction was complete as adjudged by HPLC/MS. The reaction was diluted with water to 40 mL and solid ascorbic acid was added until the solution was colourless. Purification by RP-HPLC (Xterra, C18, 10 µ, 19 x 300 mm) using a gradient of 5%B to 65% B over 60 mins at 10 mL/min to afforded **34** (7.55 mg, 38% yield).

***Peptide 37:***

The linear sequence was assembled on Fmoc-Cys(Trt) 2-chlorotrityl polystyrene resin (0.1 mmol, 0.6 mmol/g, 166 mg) as described for peptides **2-5**. At the completion of the sequence a final Fmoc deprotection was undertaken, the resin was washed with DMF and then methanol and dried. Cleavage using 94% TFA, 1% TIS, 2.5% DODT and 2.5% water (10 mL, v/v/v/v) for 2.5 h was followed by precipitation with cold diethyl ether and recovery by centrifugation afforded 170 mg of crude peptide (**35)**. **35** (170 mg, 0.1 mmol) was dissolved in 0.1 M Tris buffer (170 ml) at pH = 8.2 and stood open to air for 2 days and the oxidation was judged complete by HPLC analysis. Purification by RP-HPLC (Xterra, C18, 10 µ, 19 x 300 mm) using a gradient of 5%B to 65% B over 60 mins at 10 mL/min to afforded **36** (38 mg, 22% yield). [MS (ESI+) *m/z* 1656.1, (calcd. for [M+H]^+^, 1656.9), 828.5 (calcd. for [M+2H]^2+^, 828.9), 522.8 (calcd. for [M+3H]^3+^, 522.9].

**36**  (5 mg, 3.0 x 10^-3^ µmol) was dissolved in TFA ( 0.680 mL), anisole (40 µL) was added and the solution cooled to 0 ºC. Trifluoromethanesulfonic acid (80 µL) was added and the solution stood for 4 mins and the peptide was isolated by precipitating from ether and centrifugation. Purification by RP-HPLC (Gemini, C18, 5 µ, 10 x 250 mm) using a gradient of 5%B to 40% B over 35 mins at 5mL/min to afforded **37** (3.68 mg, 22% yield).

**36**

**#**

**T=48 h**

**T=0 h**

**35**

**Figure S4.** HPLC profile of the oxidation of **35.**  The peak labelled (*) is the expected disulphide linked peptide. The peak labelled (**#**) is Fmoc protected **35,** which presumably is removed during the reaction and also undergoes oxidation.

***Peptide 39:***

Aminomethyl polystyrene (AM-PS) resin (0.1 g, 0.1 mmol scale) was swelled in CH_2_Cl_2_ for10 mins. HMPB linker (162 mg, 0.3 mmol) and 6-Cl-HOBt (50.8 mg, 0.3 mmol) were dissolved in DMF (2 mL) and DIC (47 µL, 0.3 mmol) was added to the mixture. The resulting solution was added to the resin and shaken for 3 h at r.t, the resin was drained and washed with DMF and CH_2_Cl_2_ (2 x 3 mL) affording HMPB-AM resin. A subsequent ninhydrin test was negative. Fmoc-allylglycine-OH (101 mg, 0.3 mmol) and HCTU (206.8 mg, 0.3 mmol) was dissolved in DMF (2 mL) and *i*Pr_2_EtN (105 µL, 0.6 mmol) was added to the mixture. The resulting solution was added to the resin and stirred for 2 h at r.t., washed with DMF and the Fmoc group was removed by treatment of the resin with 20% v/v piperidine in DMF for 5 and then for 15 min and washed with DMF.

Fmoc-Thr(*t*Bu)-OH (199 mg, 0.5 mmol) in DMF/ CH_2_Cl_2_ (1: 19, 1.25 mL) was added to a mixture of HCTU (206.8 mg, 0.5 mmol) in DMF (1.3 mL) and the resulting mixture was added to the resin and shaken for 45 min at r.t., washed with DMF and CH_2_Cl_2_. The resin was transferred to a Tribute automated peptide synthesiser reaction vessel and the peptide chain was elongated as described for peptides 2-5, except that the N-terminal Fmoc-allylglycine-OH residue was coupled manually as follows: Fmoc-allylGly (101 mg, 0.3 mmol) and HCTU (206.8 mg, 0.3 mmol) was dissolved in DMF (2 mL) and *i*Pr_2_EtN (105 µL, 0.6 mmol) was added to the resin and shaken for 2 h at r.t. The resin was drained washed with DMF and the Fmoc group was deprotected by treatment of the resin with 5% piperazine containing 0.1 M 6-Cl-HOBt in DMF (3 mL) for 5 min at r.t. and then for 15 min at r.t. The resin was drained, washed with DMF and treated with a solution of Boc anhydride (115 µL, 1.0 mmol) in DMF (3 mL) for 2 h at r.t. A subsequent ninhydrin test was negative, thus affording Boc-protected peptidyl-resin **38**. **38** was placed in a microwave reactor, and a deoxygenated mixture of DMF/CH_2_Cl_2_ (4: 1, 5.0 mL) was added and the mixture further deoxygenated (Ar, 30 min). Hoveyda-Grubbs’ II catalyst (12.73 mg, 15 x 10-3 mmol) was then added and the mixture was microwaved for 1 h at 100 °C (120 W) under sealed conditions. A fresh portion of Hoveyda-Grubbs’ II catalyst (12.73 mg, 15 x 10-3 mmol) was added to the deoxygenated resin mixture, which was microwaved for 1 h at 100 °C (120 W) under sealed conditions. The resin was drained, washed with DMF, DMSO (15 mL) added and stirred overnight. Peptidyl-resin was drained, washed with DMF and CH_2_Cl_2_ and dried under a nitrogen flow. Cleavage using 94% TFA, 1% TIS, 2.5% DODT and 2.5% water (10 mL, v/v/v/v) for 2.5 h was followed by precipitation with cold diethyl ether and recovery by centrifugation and finally purified by semi-preparative RP-HPLC on a Phenomenex Gemini C18 column (5 µ, 110 Å, 10.0 x 250 mm) running a gradient of 5-32% (1% MeCN per min). This afforded **39** as a white amorphous solid (2.26 mg, 1.4%).


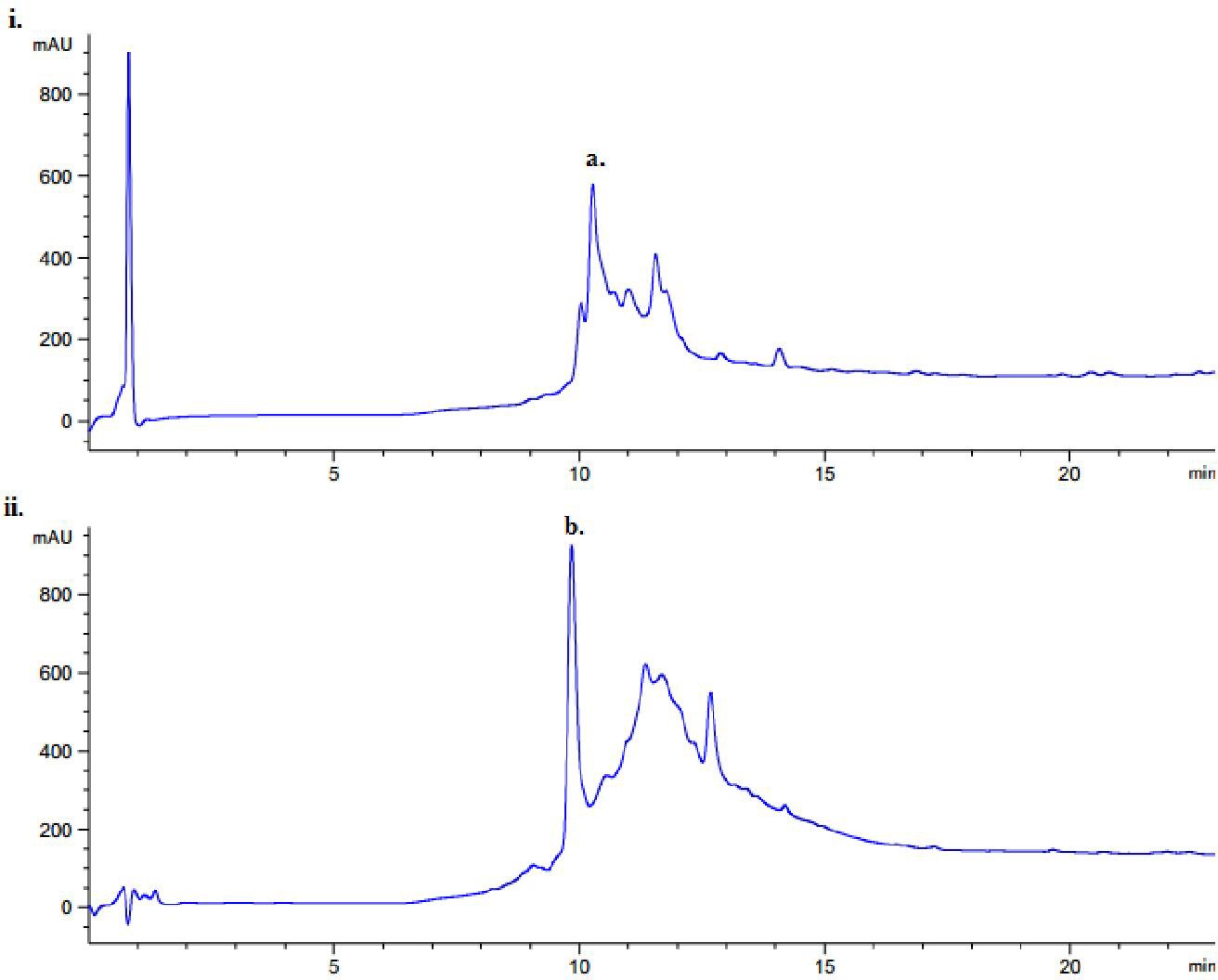


*

**Figure S5.** LC-MS profile following metathesis of resin **38** and peptide cleavage to afford **39.**  The peak labelled (*) is the expected metathesis product.

***Peptide 40:***

HMPB-AM resin was prepared as described for peptide **39** and Fmoc-allylglycine-was coupled as descried for peptide **39**. The Fmoc group of allylglycine was deprotected by treatment of the resin with 20% v/v piperidine in DMF for 5 min and then for 15 min with fresh reagents. Fmoc-Thr(*t*Bu)-OH (199 mg, 0.5 mmol) in DMF/ CH_2_Cl_2_ (1: 19, 1.25 mL) was added to a mixture of HCTU (206.8 mg, 0.5 mmol) in DMF (1.3 mL) and the resulting mixture was added to the resin and shaken for 45 min at r.t. The resin was filtered and washed with DMF and CH_2_Cl_2_ A subsequent ninhydrin test was negative. The Fmoc group of threonine was deprotected by treatment of the resin with 20% v/v piperidine in DMF for 5 min drained and then a fresh portion of 20% v/v piperidine in DMF was added to the resin for 15 min .. The resin was drained and washed with DMF (3 x 2 mL). Fmoc-allylglycine-OH (101 mg, 0.3 mmol) and HCTU (206.8 mg, 0.3 mmol) was dissolved in DMF (2 mL) and *i*Pr_2_EtN (105 µL, 0.6 mmol) was added to the resin. and stirred for 2 h at r.t. The resin was drained and washed with DMF. The resin was transferred to a Tribute automated peptide synthesiser reaction vessel and the peptide chain was elongated as described in for peptides **2-5**. The resin was treated with a solution of Boc anhydride (115 µL, 1.0 mmol) in DMF (3 mL) for 2 h at r.t. and subsequent ninhydrin test was negative, thus affordind Boc- protected peptidyl-resin **41**. The resulting Boc-protected peptidyl-resin **41** was placed in a microwave reactor, a deoxygenated mixture of DMF/CH_2_Cl_2_ (4: 1 *v/v*, 5.0 mL) was added and the mixture further deoxygenated (Ar, 30 min). Hoveyda-Grubbs’ II catalyst (12.73 mg, 15 x 10-3 mmol) was then added and the mixture was microwaved for 2 h at 100 °C (120 W) under sealed conditions. The peptidyl-resin was drained, washed with DMF (3 x 5 mL), DMSO (15 mL) added and stirred overnight. Peptidyl-resin was drained, washed with DMF and CH_2_Cl_2_ (5 x 3 mL), and dried under a nitrogen flow. The peptide was cleaved from the resin using TFA: H_2_O: DODT: *i*Pr_2_SiH (94%: 2.5%: 2.5%: 1% v/v, 10 mL) for 2 h at r.t., was followed by precipitation with cold diethyl ether and recovery by centrifugation and finally purified on a Phenomenex Gemini C18 column (5 µ, 110 Å, 10.0 x 250 mm) running a gradient of 5-32% (1% MeCN per min) This afforded **40** as a white amorphous solid (3.75 mg, 2.6%)


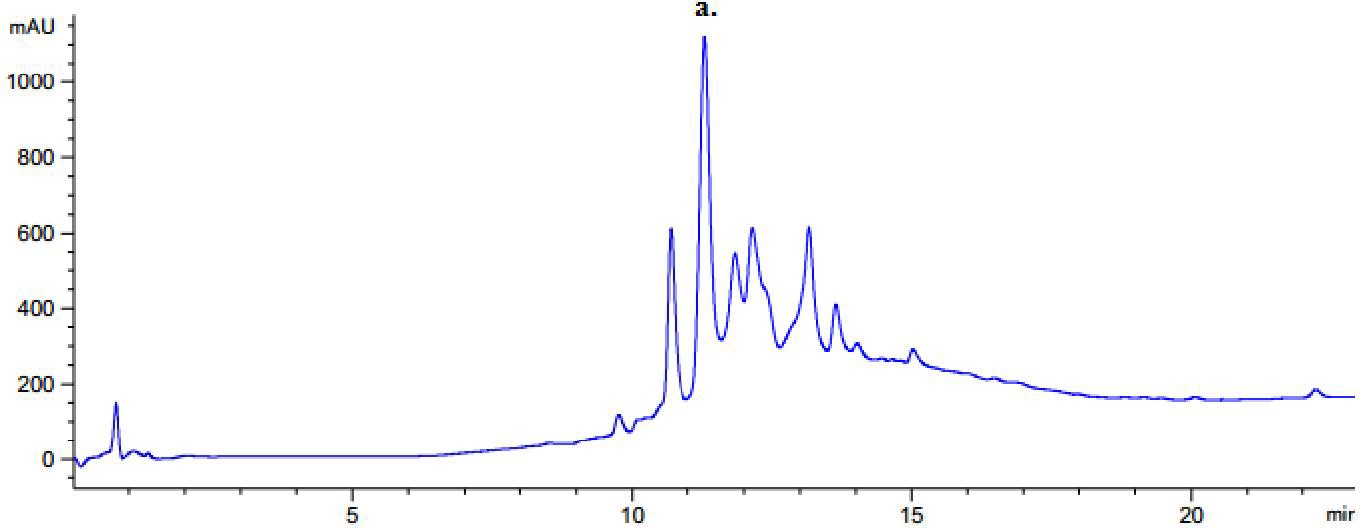


*

**Figure S6.** LC-MS profile following metathesis of resin **41** and peptide cleavage to afford **40.**  The peak labelled (*)is the expected metathesis product.

***Peptide 42:***

Fmoc-Thr(*t*Bu)-HMPP-AM resin was prepared as described for peptides **2-5**. The Fmoc group of threonine was deprotected by treatment of the resin with 20% v/v piperidine in DMF for 5 min at drained and then a fresh portion of 20% v/v piperidine in DMF was added to the resin for 15 min at r.t. The resin was drained and washed with DMF (3 x 2 mL). Fmoc-allylglycine-OH (101 mg, 0.3 mmol) and HCTU (206.8 mg, 0.3 mmol) was dissolved in DMF (2 mL) and *i*Pr_2_EtN (105 µL, 0.6 mmol) was added to the mixture. The resulting solution was added to the resin and stirred for 2 h The resin was drained and washed with DMF (3 x 2 mL). The Fmoc group of allylglycine was deprotected by treatment of the resin with 20% v/v piperidine in DMF for 5 min at r.t. drained and then a fresh portion of 20% v/v piperidine in DMF was added to the resin for 15 min. The resin was drained and washed with DMF. The resin was transferred to a Tribute automated peptide synthesiser reaction vessel and the peptide chain was elongated as described for peptides **2-5**. The resin was drained and washed with DMF (3 x 2 mL). Fmoc-allylglycine-OH (101 mg, 0.3 mmol) and HCTU (206.8 mg, 0.3 mmol) was dissolved in DMF (2 mL) and *i*Pr_2_EtN (105 µL, 0.6 mmol) was added to the mixture. The resulting solution was added to the resin and stirred for 2 h, drained and washed with DMF. The Fmoc group of allylglycine was deprotected by treatment of the resin with 5% piperazine containing 0.1 M 6-Cl-HOBt in DMF (3 mL) for 5 min and then for 15 min at r.t. The resin was drained and washed with DMF and the resin was treated with a solution of Boc anhydride (115 µL, 1.0 mmol) in DMF (3 mL) for 2 h; a subsequent ninhydrin test was negative, thus affording Boc-protected peptidyl-resin **43.** **43** was placed in a microwave reactor, a deoxygenated mixture of DMF/CH_2_Cl_2_ (4: 1, 5.0 mL) was added and the mixture further deoxygenated (Ar, 30 min). Hoveyda-Grubbs’ II catalyst (12.73 mg, 15 x 10-3 mmol) was then added and the mixture was microwaved for 1 h at 100 °C (120 W) under sealed conditions. A fresh portion of Hoveyda-Grubbs’ II catalyst (12.73 mg, 15 x 10-3 mmol) was added to the deoxygenated resin mixture, which was microwaved for 1 h at 100 °C (120 W) under sealed conditions. The peptidyl-resin was drained, washed with DMF, DMSO (15 mL) added and stirred overnight, drained, washed with DMF and CH_2_Cl_2_ and dried under a nitrogen flow. The peptide was cleaved from the resin using TFA: H_2_O: DODT: *i*Pr_2_SiH (94%: 2.5%: 2.5%: 1% v/v, 10.0 mL) for 2 h, followed by precipitation with cold diethyl ether and recovery by centrifugation and purified on a Phenomenex Gemini C18 column (5 µ, 110 Å, 10.0 x 250 mm) running a gradient of 5-32% (1% MeCN per min). This afforded **42** as a white amorphous solid (3.47 mg, 2.4% yield).


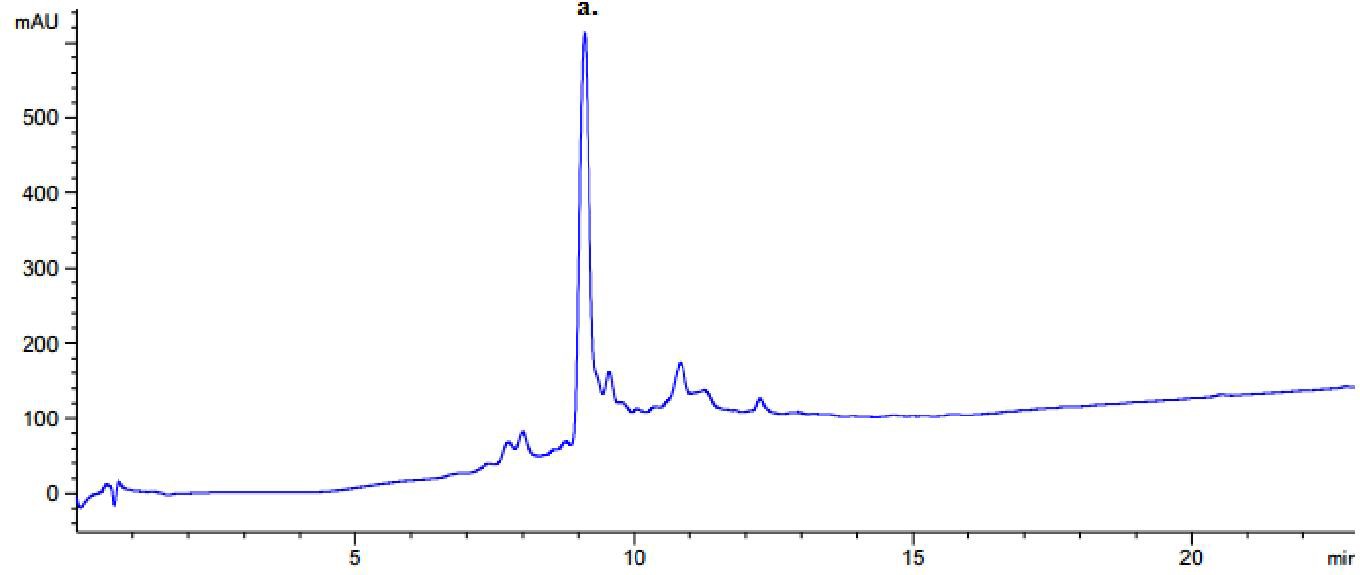


*

**Figure S7.** LC-MS profile following metathesis of resin **43** and peptide cleavage to afford **42.**  The peak labelled (*) is the expected metathesis product.

# Biological assays

### Cell culture

Human cerebral microvascular endothelial cells (hCMVEC) were purchased from Applied Biological Materials (ABM) Inc, Canada (cat # T0259) (O'Carroll et al., 2015). Cells were cultured in 1 μg/cm^2^ collagen I (Gibco) coated T75 flasks from Corning (NY, USA). The growth media was M199 media (Gibco), supplemented with 10% FBS, 1 μg/mL hydrocortisone (Sigma), 3 μg/mL human FGF (Peprotech), 10 μg/mL human EGF (Peprotech), 10 μg/mL heparin (Sigma), 2 mM Glutamax (Gibco), and 80 μM butyryl cAMP (Sigma).

### In vitro bioluminescence ATP assay

For experiments hCMVECs were plated in M199 media at a density of 2.5 x 10^4^ cells per well in a collagen-coated (1 µg/cm^2^) 12-well plate the day prior to the experiment. Hypoxic, acidic, ion-shifted Ringer (HAIR) injury solution that mimics ionic concentrations and acid-base shifts of the interstitial space in hypoxic-ischemic brains (Bondarenko & Chesler, 2001) was used to trigger hemichannel opening as described previously (Kim et al., 2017). HAIR solution (38 mM NaCl, 13 mM NaHCO_3_, 3 mM Na-gluconate, 65 mM K-gluconate, 38 mM NMDG-Cl, 1 mM NaH_2_PO_4_, and 1.5 mM MgCl_2_) was bubbled in 95% N_2_, 5% CO_2_ gas (20 L/min) for 5 minutes and pH adjusted to 6.6 before use. To induce ischemic, cells were incubated for 2 hours in 500 µL HAIR solution +/- carbenoxolone (100 µM), Peptide5 or modified analogues (100 µM). For the negative control, hCMVEC cells were incubated for 2 hours in 500 µL standard Ringer solution 124 mM NaCl, 3 mM KCl, 26 mM NaHCO_3_, 26 mM NaHCO_3_, 1 mM NaH_2_PO_4_, 1.3 mM CaCl_2_, 1.5 MgCl_2_, and 10 mM glucose) adjusted to pH 7.4 before use. All incubations were carried out at 37 °C in 95% O_2_ and 5% CO_2_. At the end of each experiment, samples were removed and immediately placed on ice. The concentration of ATP in the samples was determined using a luciferin/luciferase bioluminescence reaction (ATP Determination Kit, Molecular Probes) and detected using a luminescence plate reader (FLUOStar OPTIMA FL, BMG Labtech). Standard curves were generated for each experiment from an ATP standard (0 to 500 nM) and used to convert bioluminescence units to ATP concentration. Treatment groups had a sample size of 2 wells per experiment, and the concentration of ATP in each sample was measured in triplicate over ten repeated readings, every 2 minutes for 20 minutes. Only readings in the plateau phase of the reaction were included in averaging for the well value. Each peptide was tested in at least an n of 2 separate experiments.

**Scrape Loading assay**

For experiments hCMVECs cells were plated M199 media at a density of 4 x 10^5^ cells per well in a collagen coated (1 µg/cm^2^) 12-well plate the day prior to the experiment to ensure a confluent monolayer of hCMVECs. On the day of the experiment cells were pre-incubated in carbenoxolone, (100 µM) Peptide5 (5–100 µM) or modified analogues (100 µM) or M199 as a negative control. Cells were washed three times with phosphate buffered saline (PBS) without Ca^2+^ or Mg^2+^ and incubated in 0.05% Lucifer Yellow (LY) (Sigma), a fluorescent dye that is transferred through coupled gap junction channels (el-Fouly, Trosko, & Chang, 1987), and scrape-wounded with a size 10 carbon steel surgical blade (Swann-Morton, England). Following a 5 minute incubation at 37 °C in 95% O_2_ and 5% CO_2_ without light, the 0.05% LY solution was removed. Cells were rinsed four times with HBSS containing Ca^2+^ ^+^, and then fixed in 4% paraformaldehyde (PFA) in PBS at pH 7.4 for 10 minutes at room temperature. Cells were then washed 3 times in PBS to remove PFA before fluorescent imaging. Fluorescent images were visualized using a Nikon TE2000E inverted fluorescent microscope (10x magnification, 0.3 numerical aperture), and captured using a Digital Sight CCD camera and Eclipse Net software (Nikon). Three images within each well from three independent experiments were taken for analysis, and the total number of cells showing dye uptake from those that had been loaded were counted manually by masked observers. Each peptide was tested in at least an n of 2 separate experiments.

# Human serum stability assay

**a) Serum collection**

Whole blood was collected from a healthy volunteer and stored in a covered test tube and was allowed to clot by leaving it undisturbed at r.t. for *ca.* 1 h. The clot was removed by centrifugation at 1,000-2,000 x g for 10 min and the resulting supernatant was designated as the serum. The serum was collected and stored in 0.5 mL aliquots, whereby the samples were stored at - 20 °C.

**b) Experimental protocol**

200 µM concentration of peptide was incubated in 25% aq. human serum (1.7 mL) and left to shake at 37 °C. Aliquots of human serum-peptide mixture (270 µL) were taken at given times (0 h, 1 h, 4 h, 8 h, 24 h, and 48 h) and threefold excess of acetonitrile (810 µL) was added to the aliquot and left for 1 h at 0 °C. The samples underwent centrifugation (14,500 rpm) for 30 min at r.t. and the supernatant was subsequently collected. Upon evaporation of the solvents, the resulting dry, pale-yellow residue was dissolved in water containing 0.1 % TFA.

**c) Analysis**

The dissolved solution was analysed *via* analytical RP HPLC and the quantity of the peptide was expressed as a percentage relative to the peptide peak area obtained at time zero (i.e. 100% peptide amount at t = 0 h). A scatterplot graph representing percentage of peptide versus time was generated. Each graph was compared to peptide5 and other analogues to determine their level of stability within human serum.

# Characterization Data

***Peptide 2:***

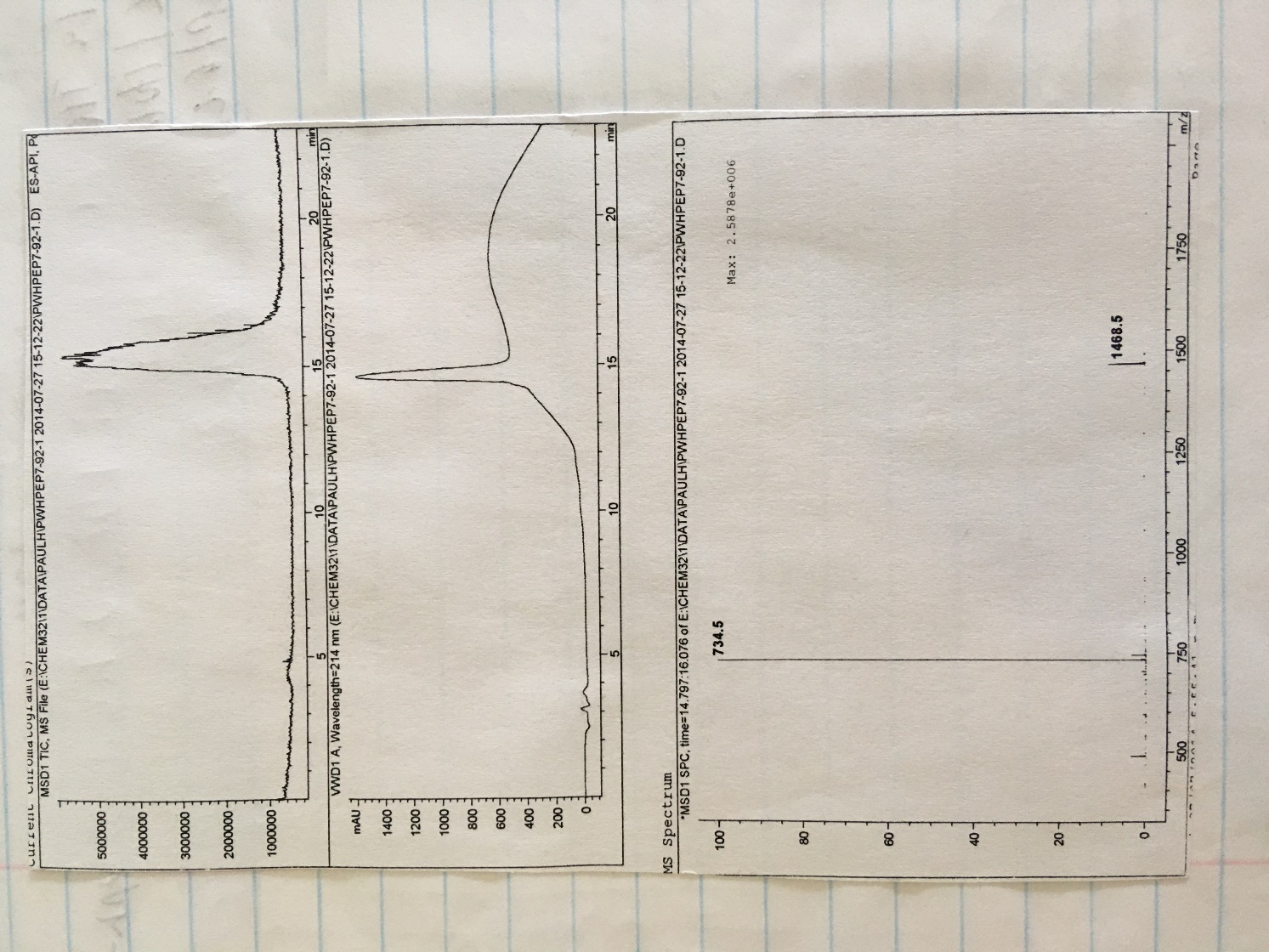


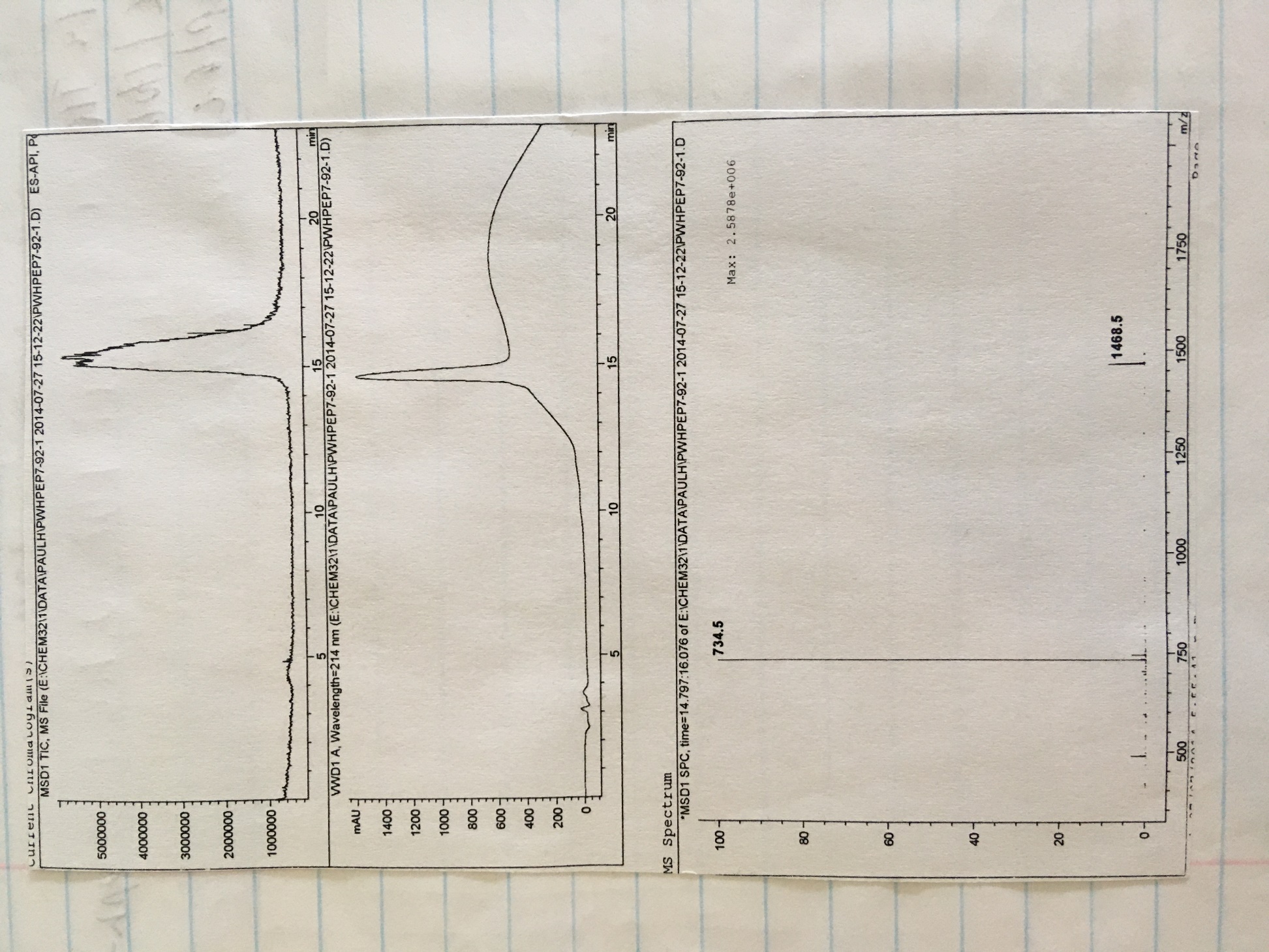


**Figure S8.** HPLC-MS spectrum of peptide **2**, *ca.* 97% purity as judged by peak area of RP-HPLC at 214 nm); Agilent C3-300SB (3.5 μm, 300 Å, 3 mm × 150 mm), linear gradient of 5% B to 65% B over 21 min, *ca.* 3% B per minute at 0.3 mL min^-1^; **MS** (ESI+) *m/z* 1468.5 (calcd. for [M+H]^+^, 1468.7), 734.5 (calcd. for [M+2H]^2+^,734.8).

***Peptide 3:***

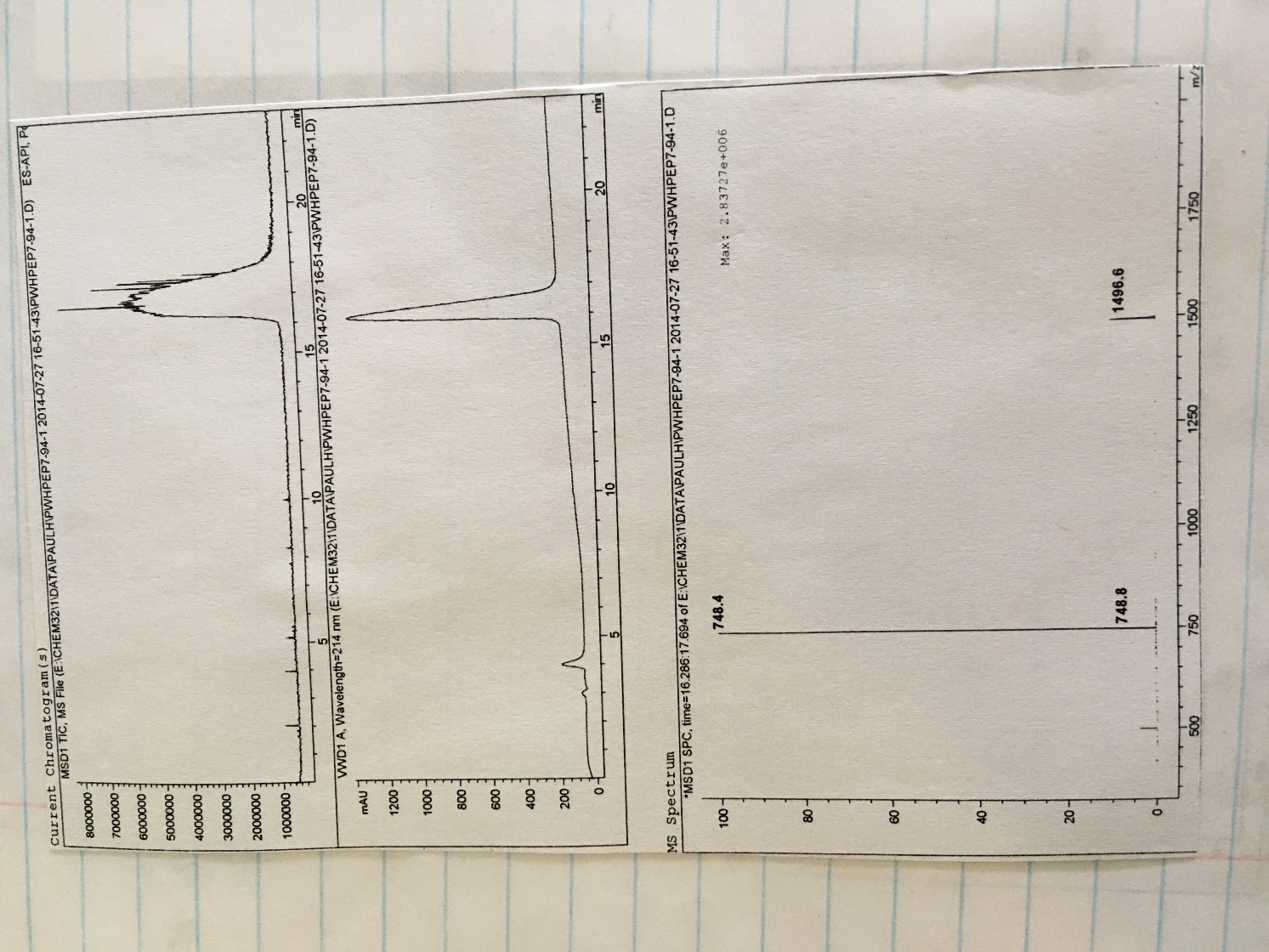


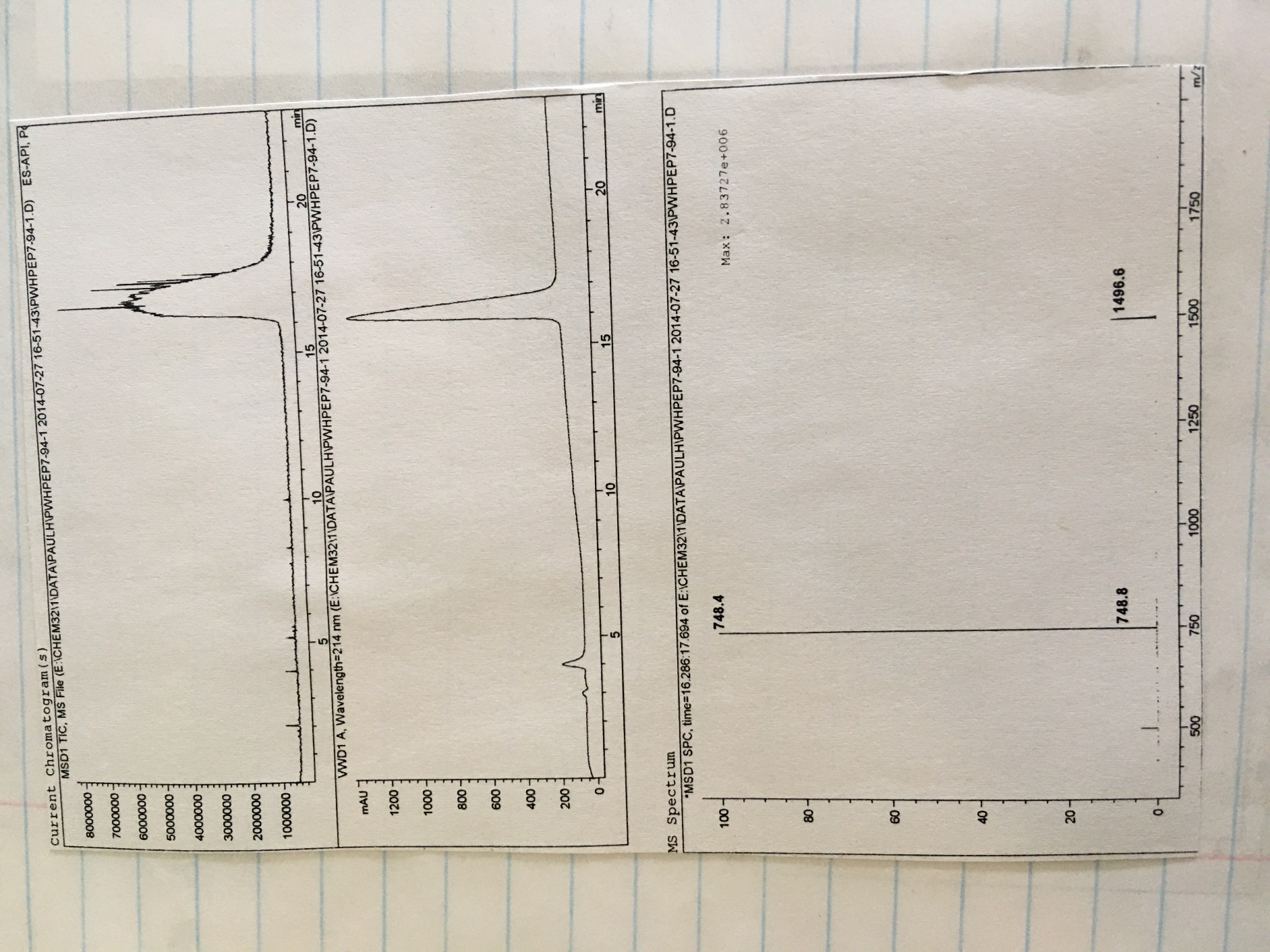


**Figure S9.** HPLC-MS spectrum of peptide **3**, *ca.* 98% purity as judged by peak area of RP-HPLC at 214 nm); Agilent C3-300SB (3.5 μm, 300 Å, 3 mm × 150 mm), linear gradient of 5% B to 65% B over 21 min, *ca.* 3% B per minute at 0.3 mL min^-1^; **MS** (ESI+) *m/z* 1496.8 (calcd. for [M+H]^+^, 1496.7), 748.4 (calcd. for [M+2H]^2+^,748.8).

***Peptide 4:***

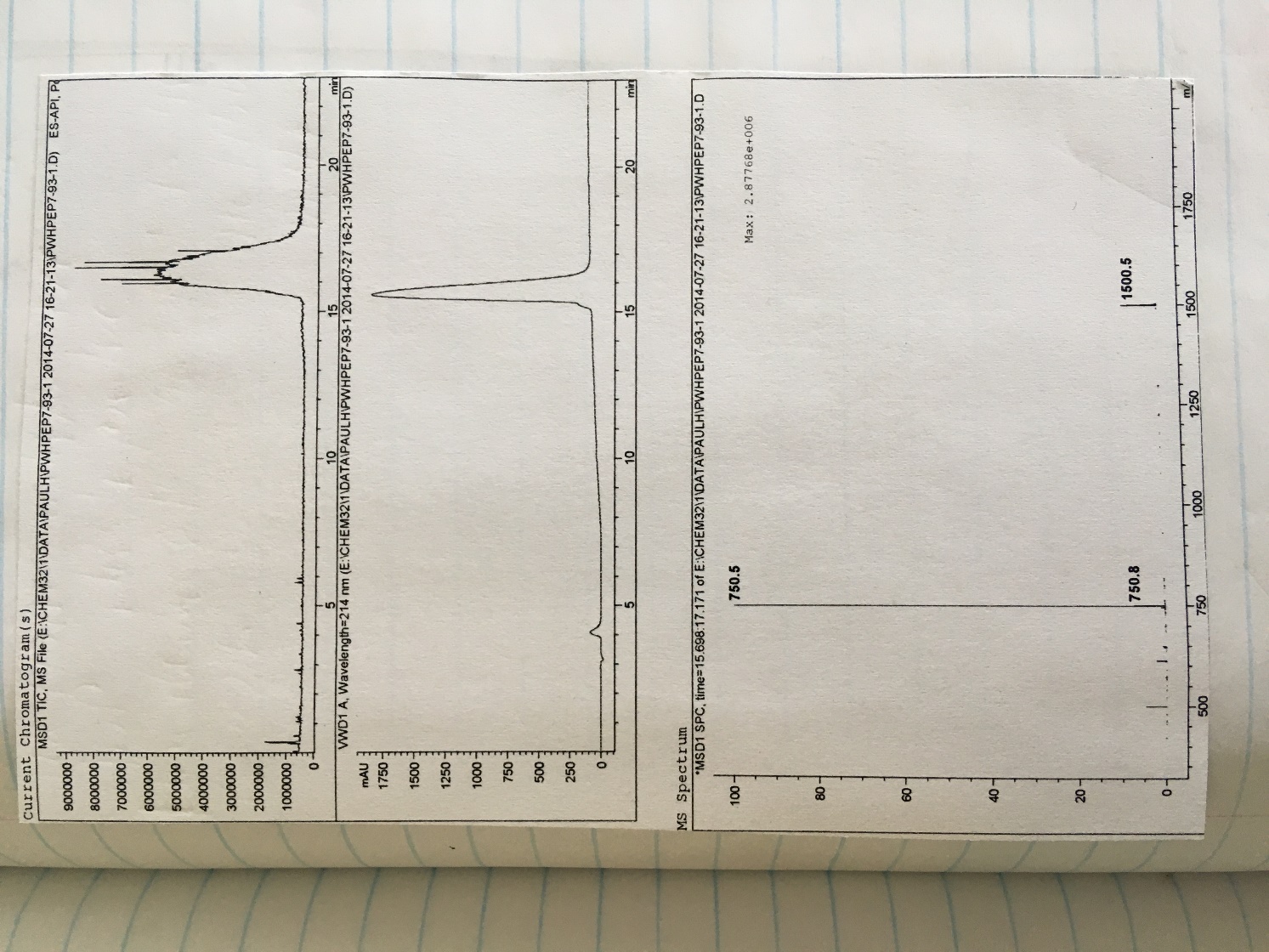


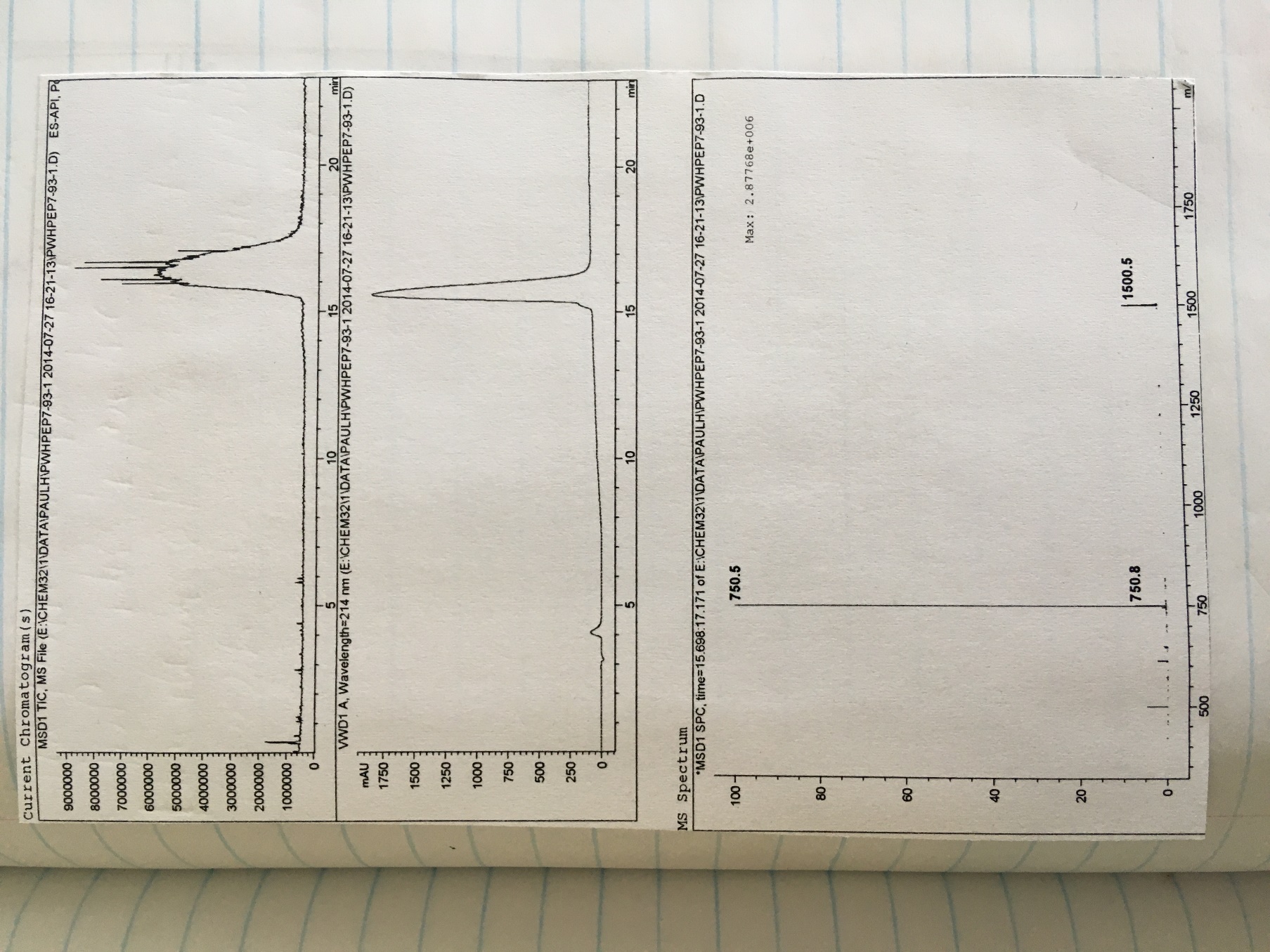


**Figure S10.** HPLC-MS spectrum of peptide **4**, *ca.* 96% purity as judged by peak area of RP-HPLC at 214 nm); Agilent C3-300SB (3.5 μm, 300 Å, 3 mm × 150 mm), linear gradient of 5% B to 65% B over 21 min, *ca.* 3% B per minute at 0.3 mL min^-1^; **MS** (ESI+) *m/z* 1500.5 (calcd. for [M+H]^+^, 1500.7), 750.5 (calcd. for [M+2H]^2+^,750.9).

***Peptide 5:***

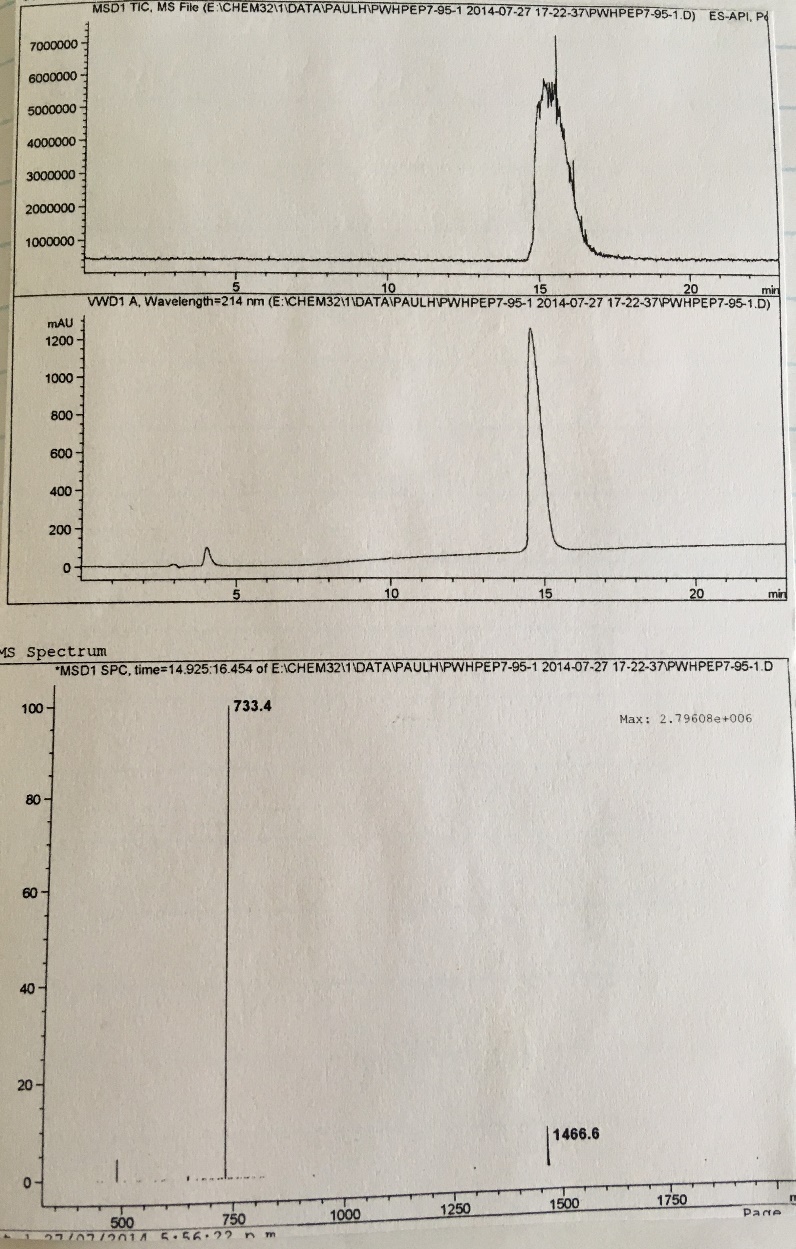


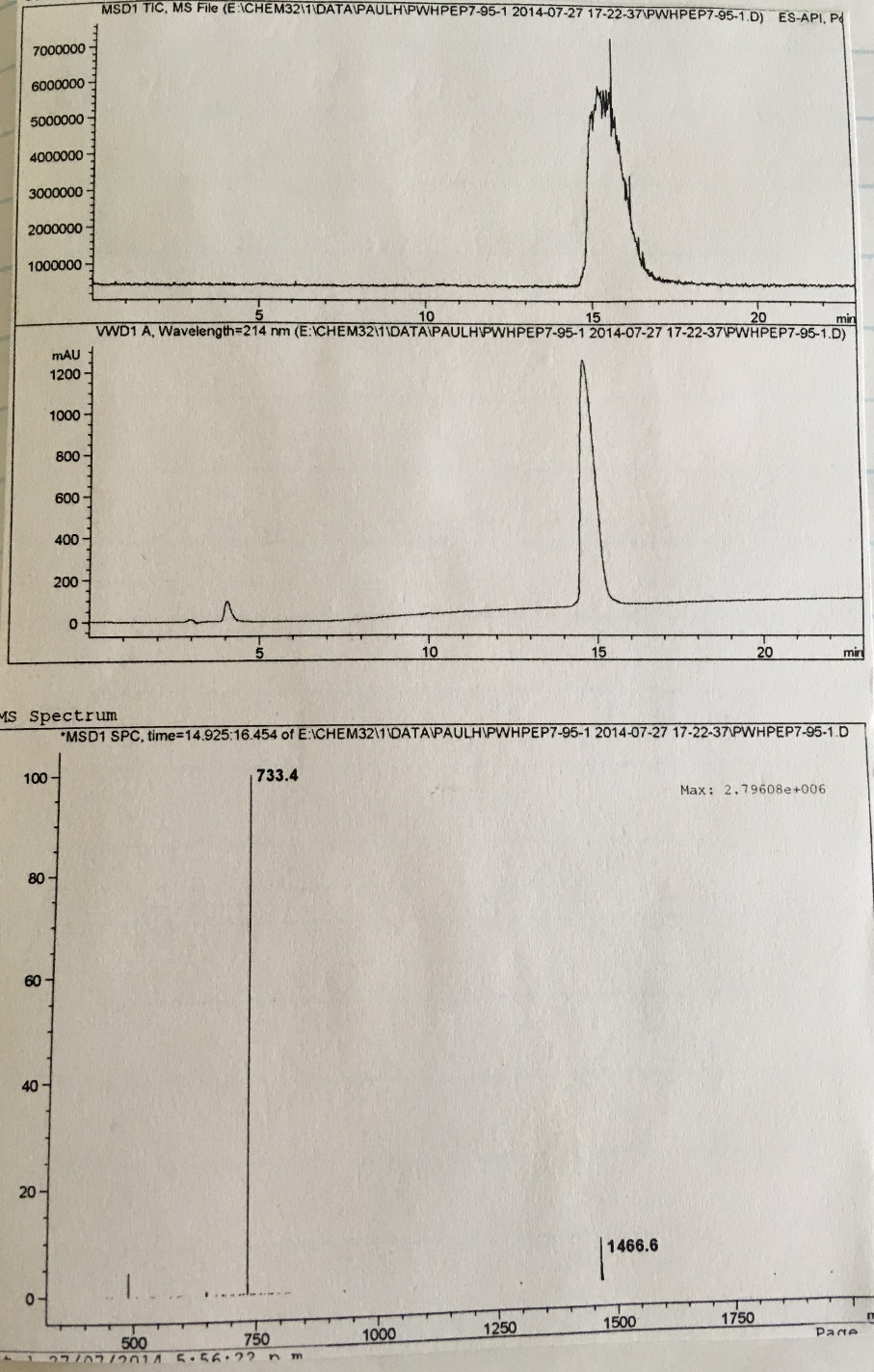


**Figure S11.** HPLC-MS spectrum of peptide **5**, *ca.* 98% purity as judged by peak area of RP-HPLC at 214 nm); Agilent C3-300SB (3.5 μm, 300 Å, 3 mm × 150 mm), linear gradient of 5% B to 65% B over 21 min, *ca.* 3% B per minute at 0.3 mL min^-1^; **MS** (ESI+) *m/z* 1466.6 (calcd. for [M+H]^+^, 1466.7), 733.4 (calcd. for [M+2H]^2+^,733.9).

***Peptide 6:***

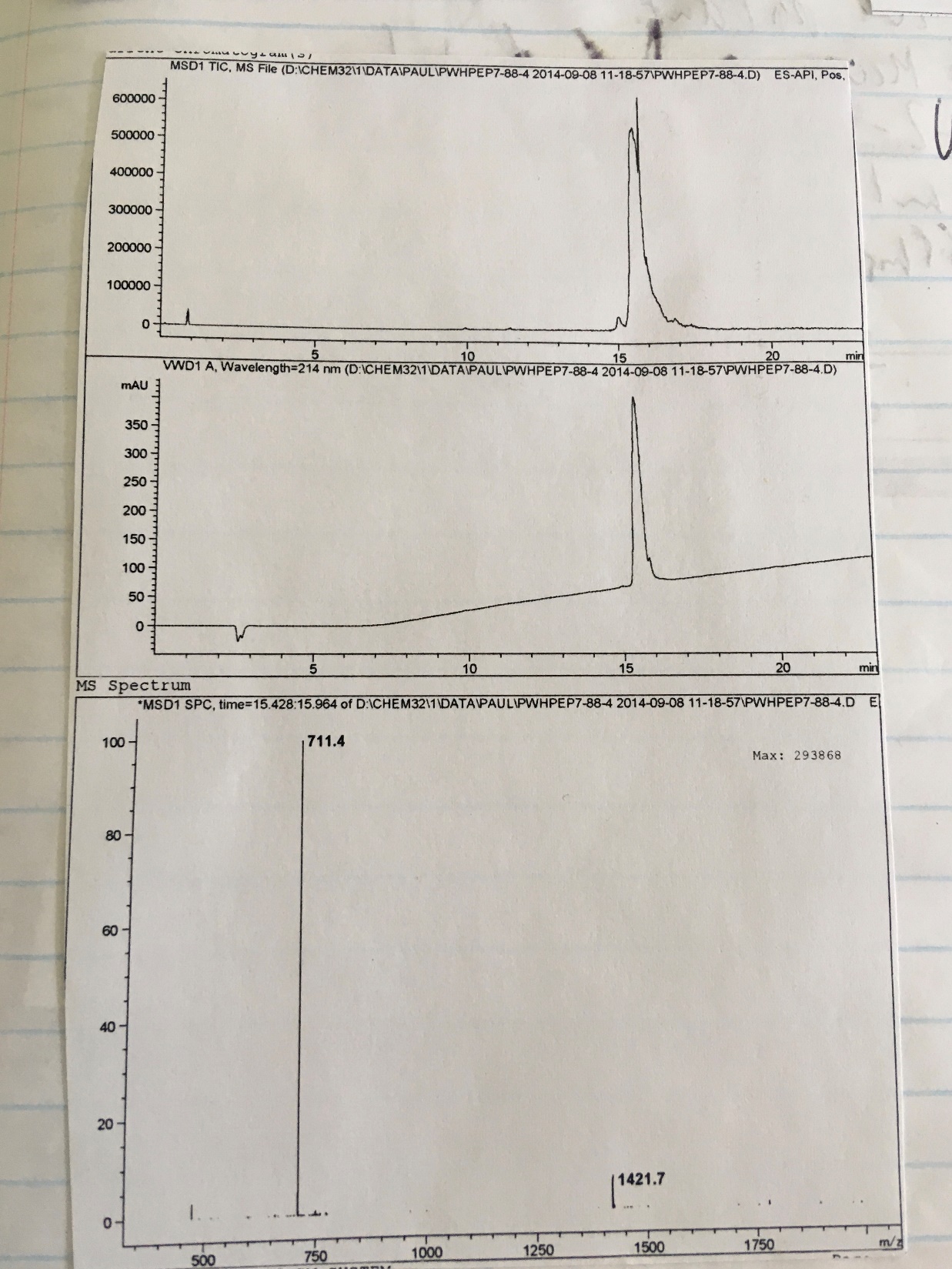


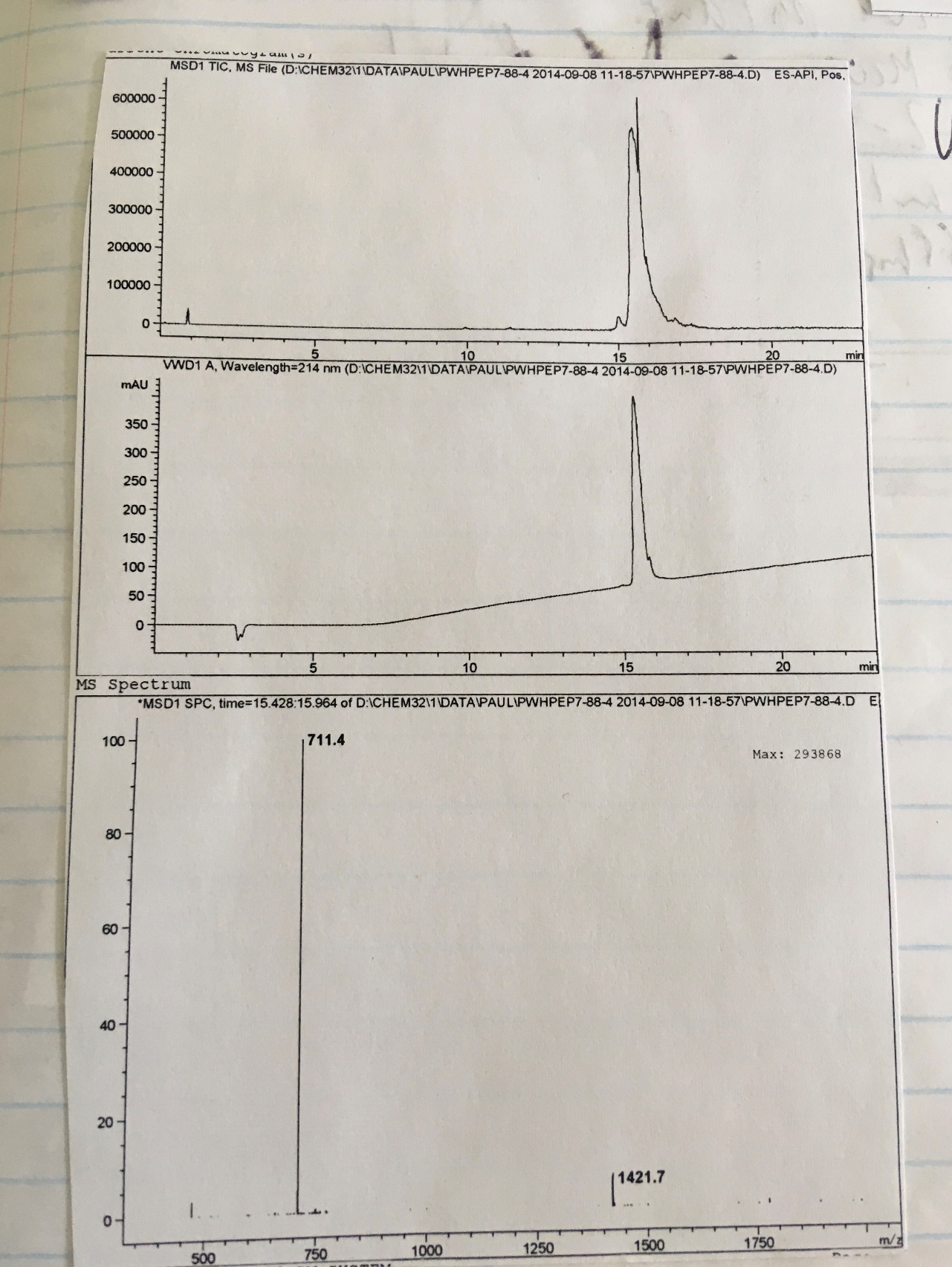


**Figure S12.** HPLC-MS spectrum of peptide **6**, *ca.* 95% purity as judged by peak area of RP-HPLC at 214 nm); Agilent C3-300SB (3.5 μm, 300 Å, 3 mm × 150 mm), linear gradient of 5% B to 65% B over 21 min, *ca.* 3% B per minute at 0.3 mL min^-1^; **MS** (ESI+) *m/z* 1421.7 (calcd. for [M+H]^+^, 1421.7), 711.4 (calcd. for [M+2H]^2+^,711.9).

***Peptide 7:***

**Figure S13.** HPLC-MS spectrum of peptide **7**, *ca.* 99% purity as judged by peak area of RP-HPLC at 214 nm); Agilent C3-300SB (3.5 μm, 300 Å, 3 mm × 150 mm), linear gradient of 5% B to 95% B over 30 min, *ca.* 3% B per minute at 0.3 mL min^-1^; **MS** (ESI+) *m/z* 705.4 (calcd. for [M+2H]^2+^ 705.7) 470.6 (calcd. for [M+3H]^3+^, 470.9).

***Peptide 8:***

**Figure S14.** HPLC spectrum of peptide **8**, *ca.* 96% purity as judged by peak area of RP-HPLC at 214 nm); ); Agilent C3-300SB (3.5 μm, 300 Å, 3 mm × 150 mm), linear gradient of 5% B to 95% B over 30 min, *ca.* 3% B per minute at 0.3 mL min^-1^; **MS** (ESI+) *m/z* 705.4 (calcd. for [M+2H]^2+^ 705.7) 470.7 (calcd. for [M+3H]^3+^, 470.9).

***Peptide 9:***

**Figure S15.** HPLC spectrum of peptide **9** *ca.* 99% purity as judged by peak area of RP-HPLC at 214 nm); ); Agilent C3-300SB (3.5 μm, 300 Å, 3 mm × 150 mm), linear gradient of 5% B to 95% B over 30 min, *ca.* 3% B per minute at 0.3 mL min^-1^; **MS** (ESI+) *m/z* 698.4 (calcd. for [M+2H]^2+^ 698.8) 466.0 (calcd. for [M+3H]^3+^, 466.2).

***Peptide 10:***

**Figure S16.** HPLC spectrum of peptide **10** *ca.* 97% purity as judged by peak area of RP-HPLC at 214 nm); ); Agilent C3-300SB (3.5 μm, 300 Å, 3 mm × 150 mm), linear gradient of 5% B to 95% B over 30 min, *ca.* 3% B per minute at 0.3 mL min^-1^; **MS** (ESI+) *m/z* 698.4 (calcd. for [M+2H]^2+^ 698.8) 466.0 (calcd. for [M+3H]^3+^, 466.2).

***Peptide 11:***

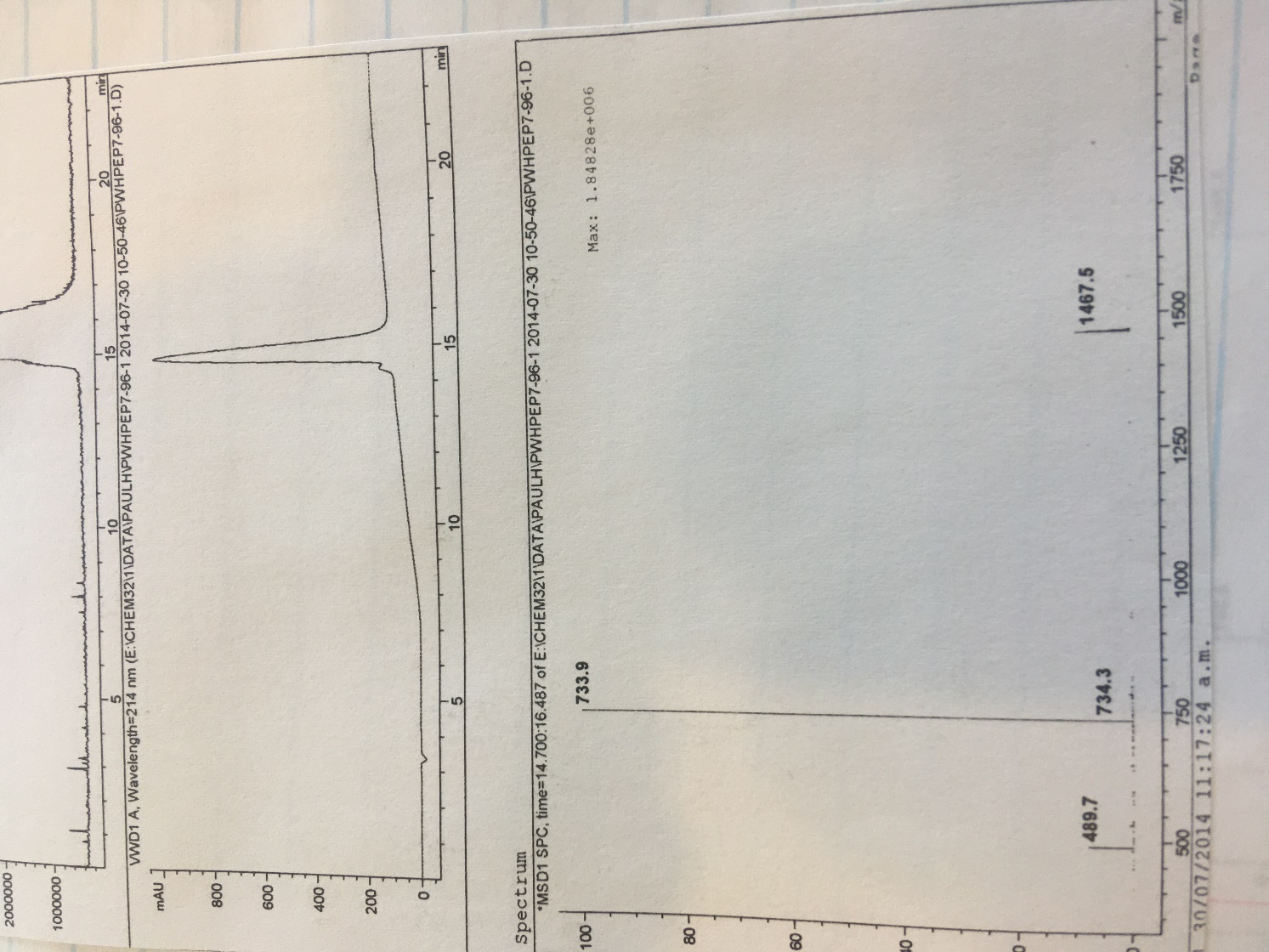


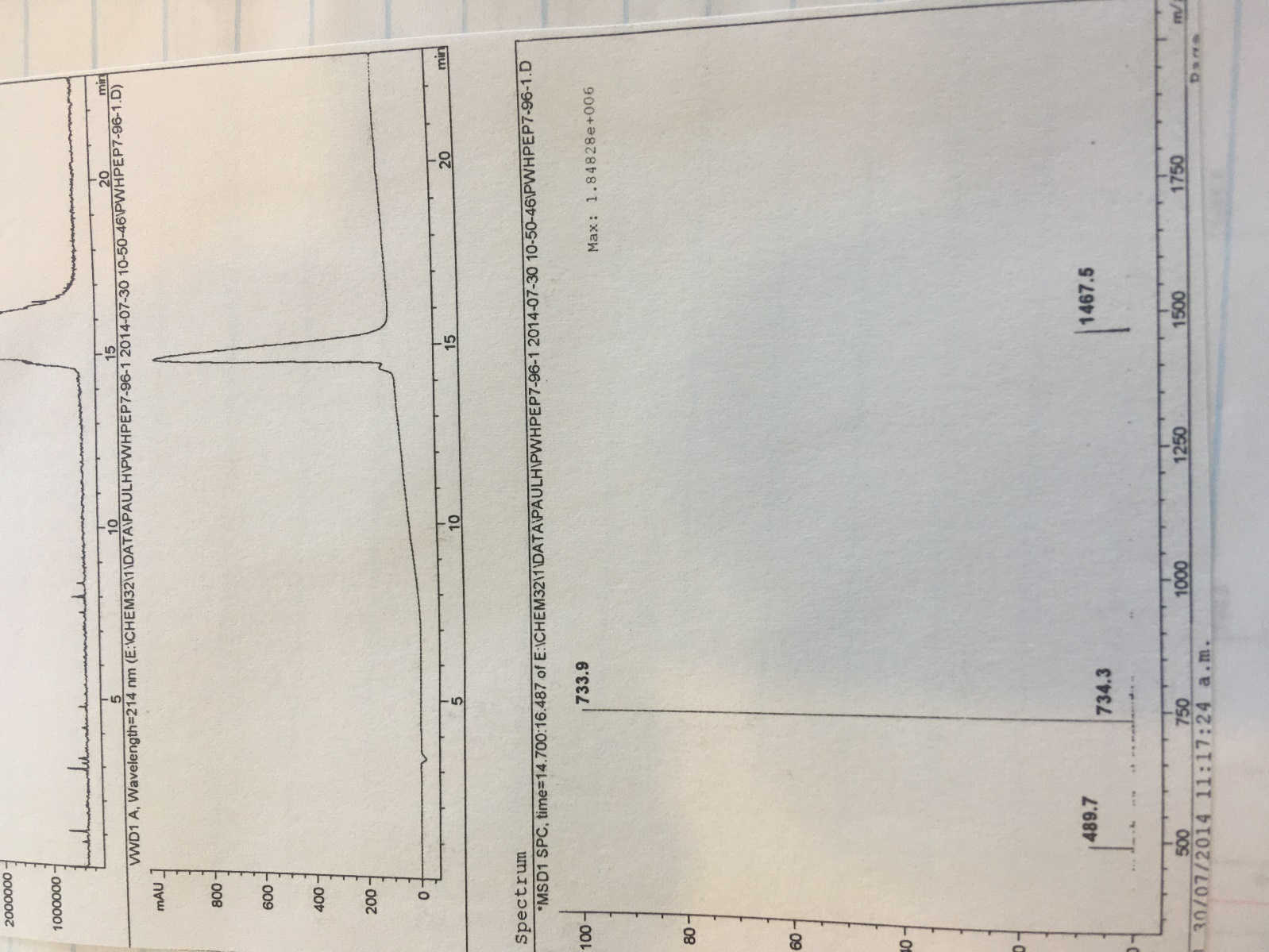


**Figure S17.** HPLC-MS spectrum of peptide **11**, *ca.* 96% purity as judged by peak area of RP-HPLC at 214 nm); Agilent C3-300SB (3.5 μm, 300 Å, 3 mm × 150 mm), linear gradient of 5% B to 65% B over 21 min, *ca.* 3% B per minute at 0.3 mL min^-1^; **MS** (ESI+) *m/z* 1467.5 (calcd. for [M+H]^+^, 1467.7), 733.9 (calcd. for [M+2H]^2+^,734.4), 489.7 (calcd. for [M+3H]^3+^,489.9).

***Peptide 12:***

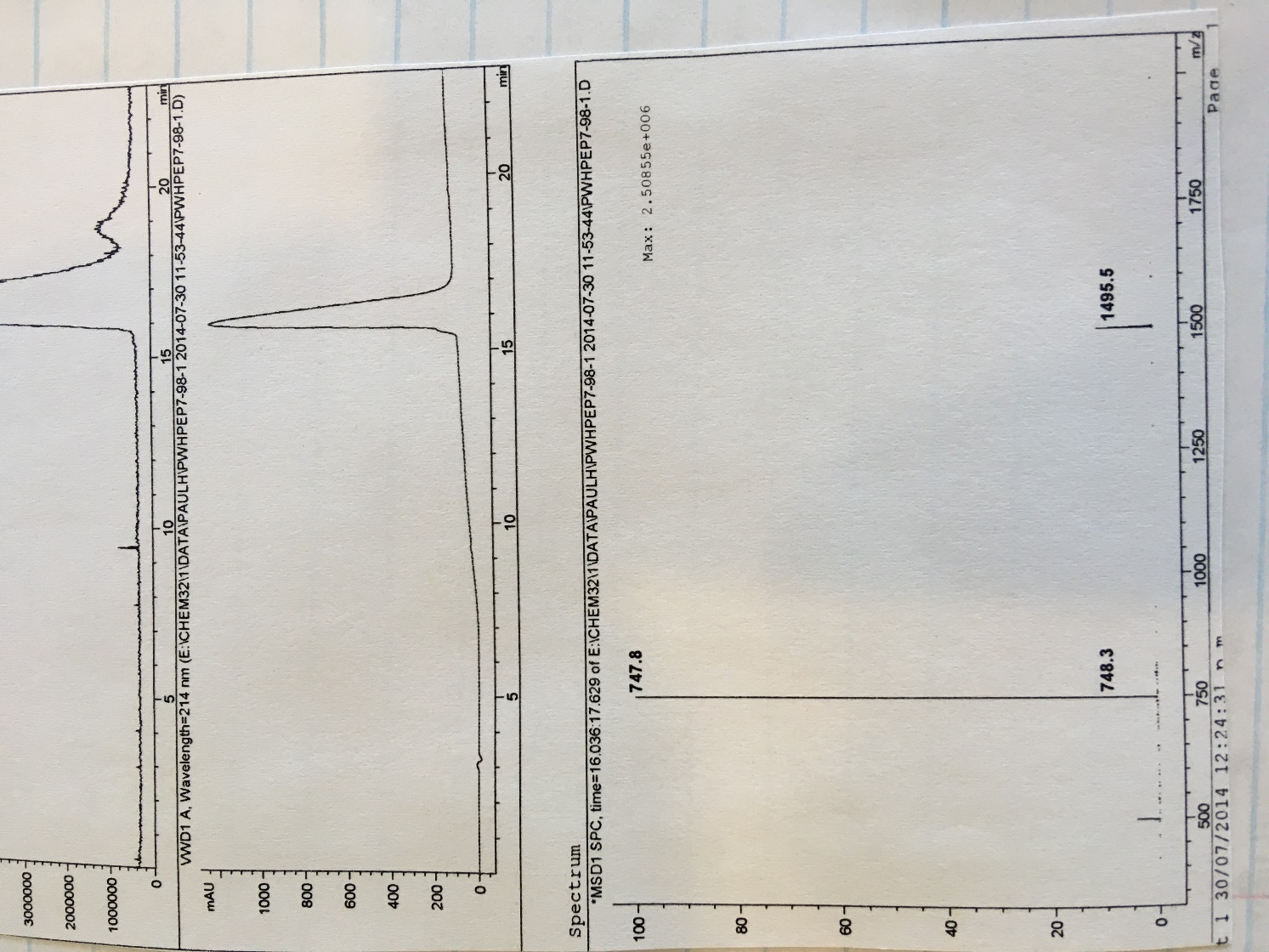


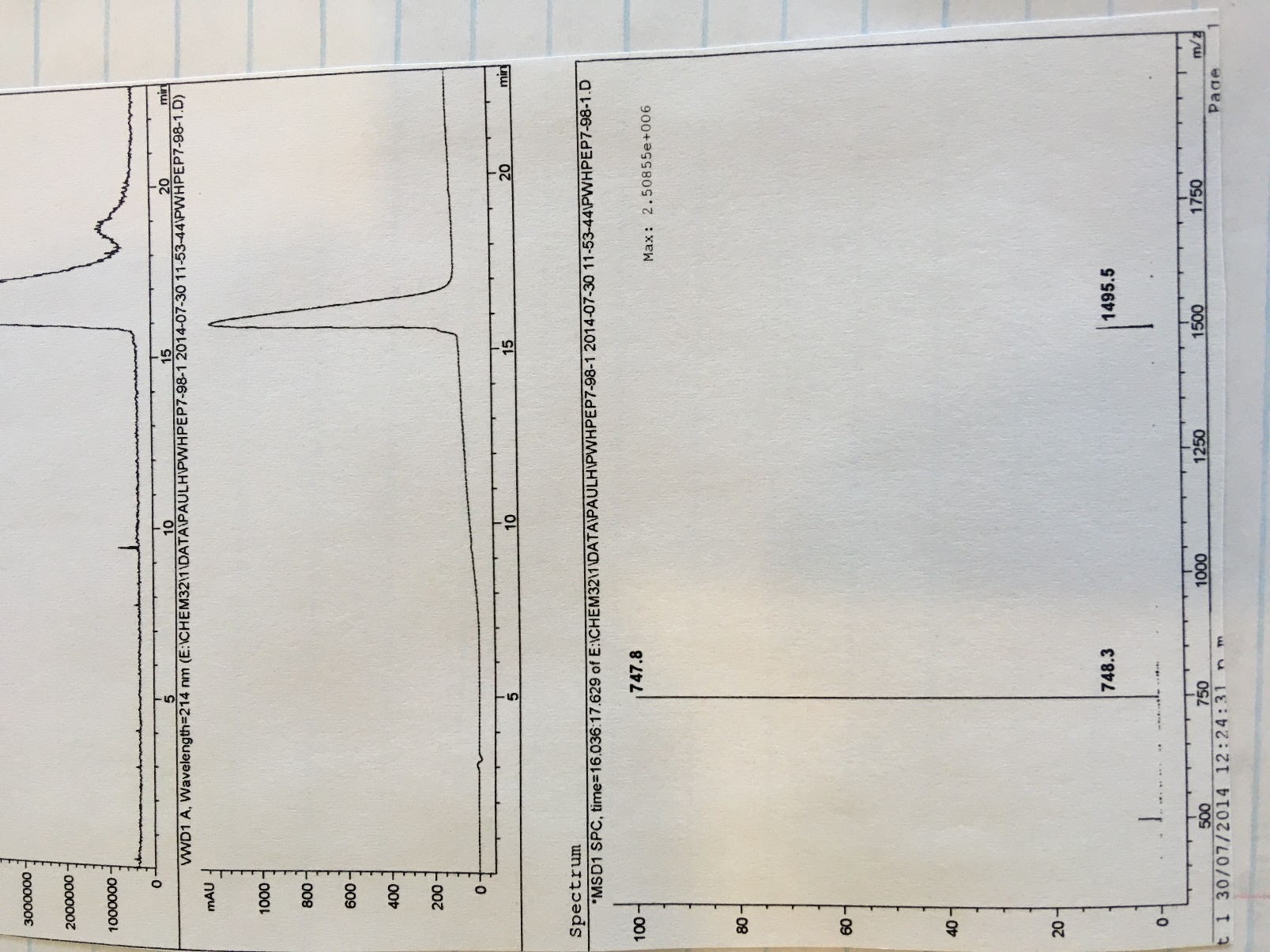


**Figure S18.** HPLC-MS spectrum of peptide **12**, *ca.* 98% purity as judged by peak area of RP-HPLC at 214 nm); Agilent C3-300SB (3.5 μm, 300 Å, 3 mm × 150 mm), linear gradient of 5% B to 65% B over 21 min, *ca.* 3% B per minute at 0.3 mL min^-1^; **MS** (ESI+) *m/z* 1495.5 (calcd. for [M+H]^+^, 1495.7), 747.8 (calcd. for [M+2H]^2+^,748.3).

***Peptide 13:***

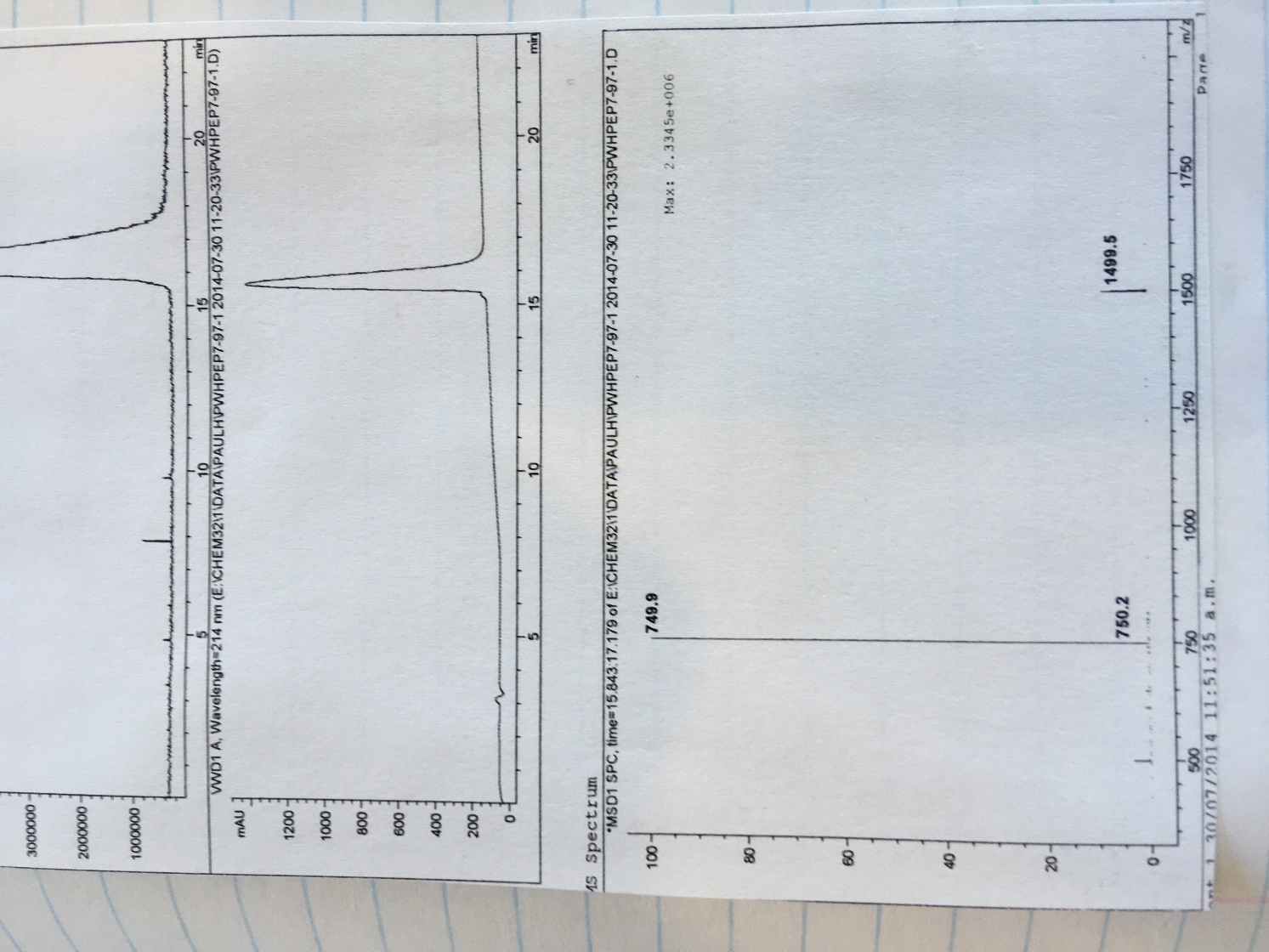


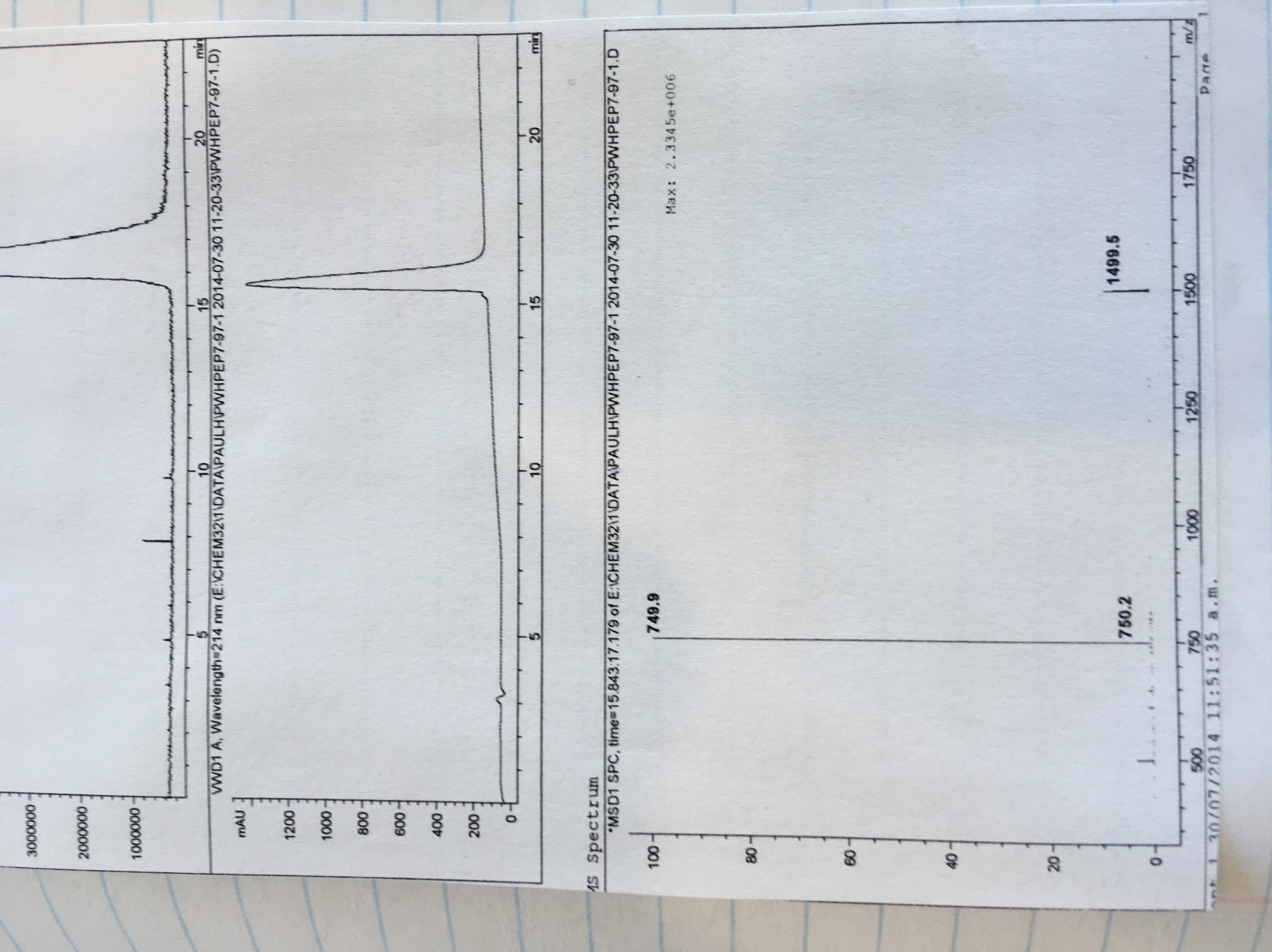


**Figure S19.** HPLC-MS spectrum of peptide **13**, *ca.* 99% purity as judged by peak area of RP-HPLC at 214 nm); Agilent C3-300SB (3.5 μm, 300 Å, 3 mm × 150 mm), linear gradient of 5% B to 65% B over 21 min, *ca.* 3% B per minute at 0.3 mL min^-1^; **MS** (ESI+) *m/z* 1499.5 (calcd. for [M+H]^+^, 1498.7), 749.9 (calcd. for [M+2H]^2+^,750.4).

***Peptide 14:***

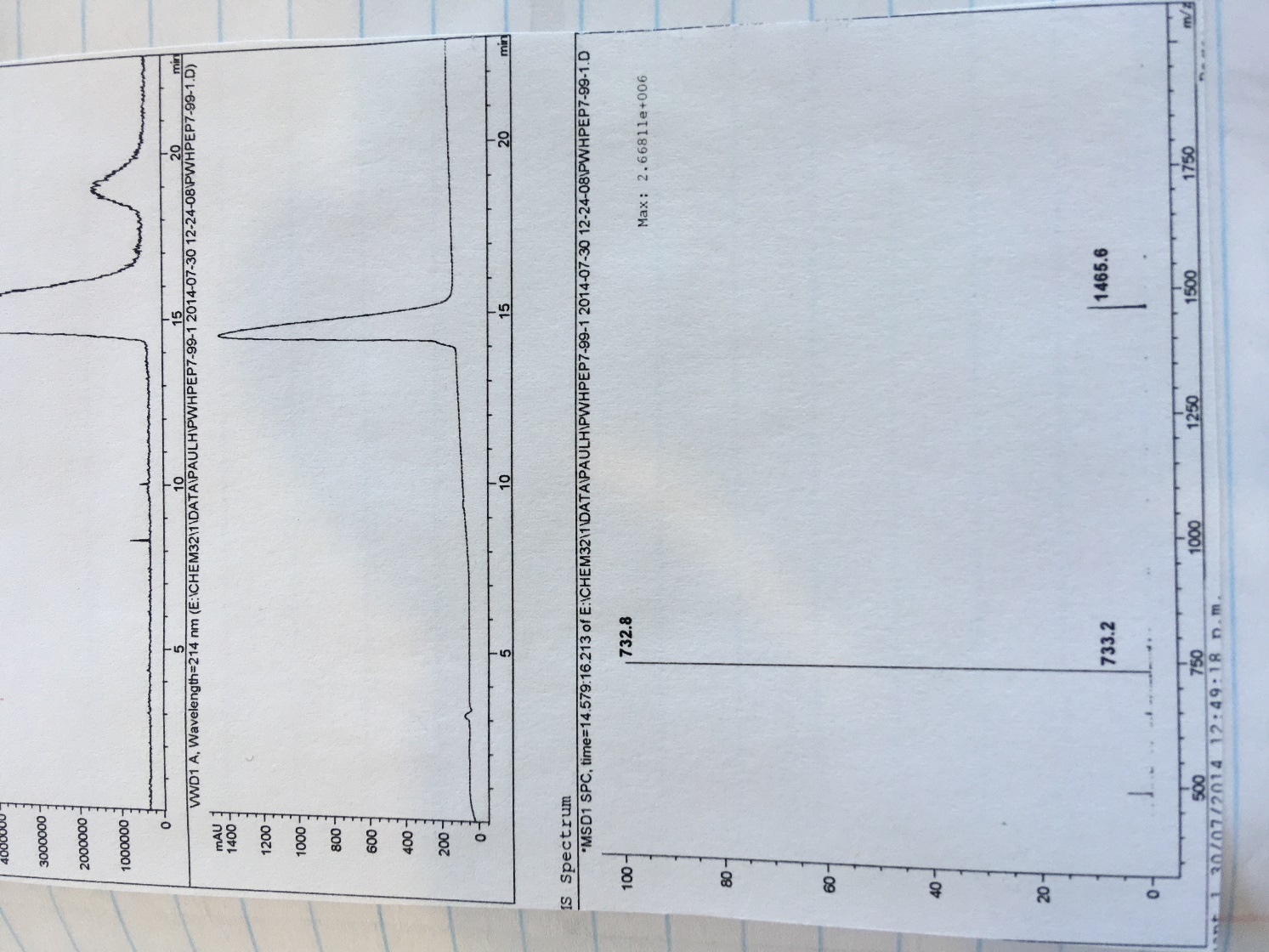


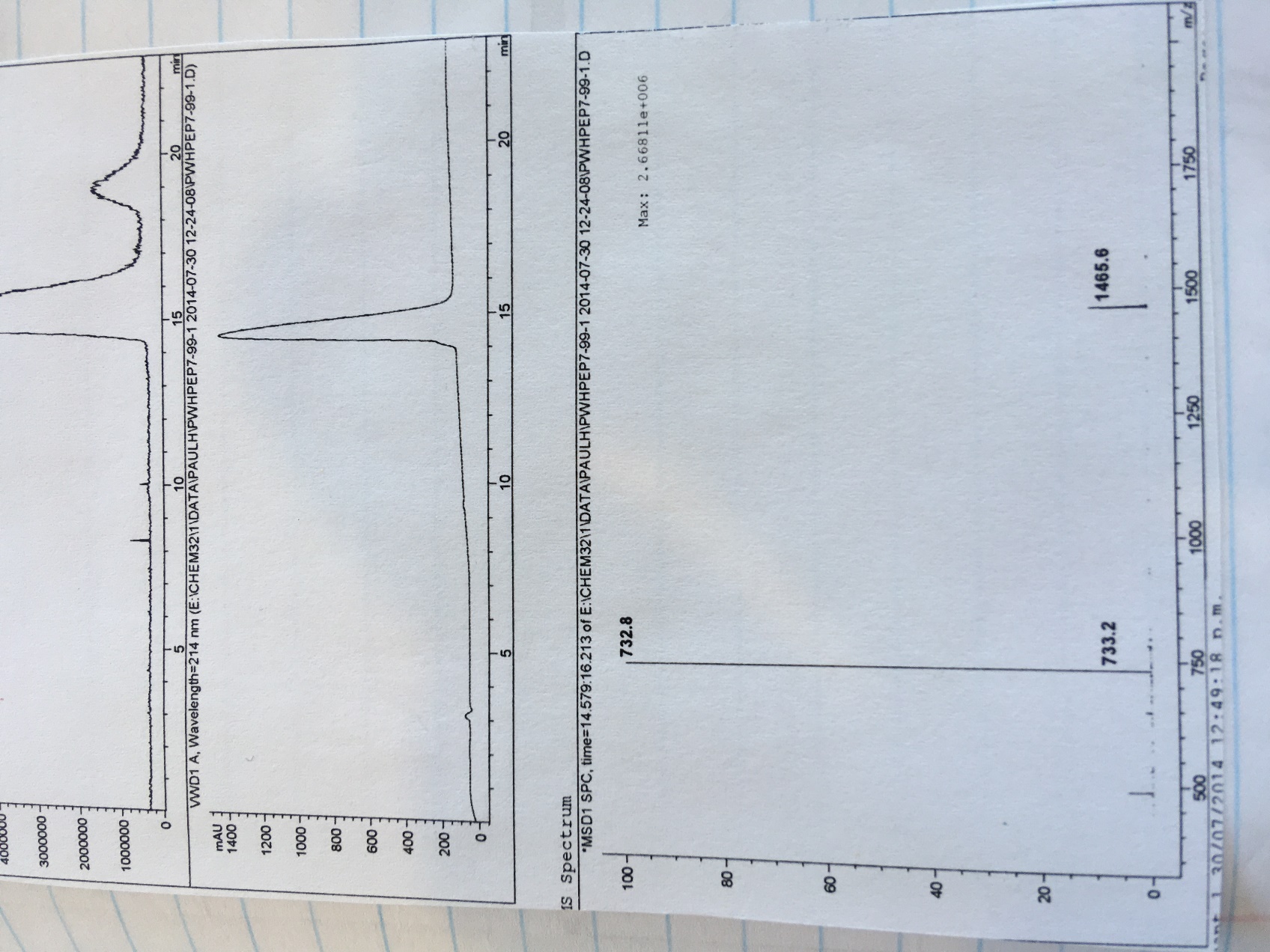


**Figure S20.** HPLC-MS spectrum of peptide **14**, *ca.* 98% purity as judged by peak area of RP-HPLC at 214 nm); Agilent C3-300SB (3.5 μm, 300 Å, 3 mm × 150 mm), linear gradient of 5% B to 65% B over 21 min, *ca.* 3% B per minute at 0.3 mL min^-1^; **MS** (ESI+) *m/z* 1465.6 (calcd. for [M+H]^+^, 1465.7), 732.8 (calcd. for [M+2H]^2+^,733.3).

***Peptide 15:***

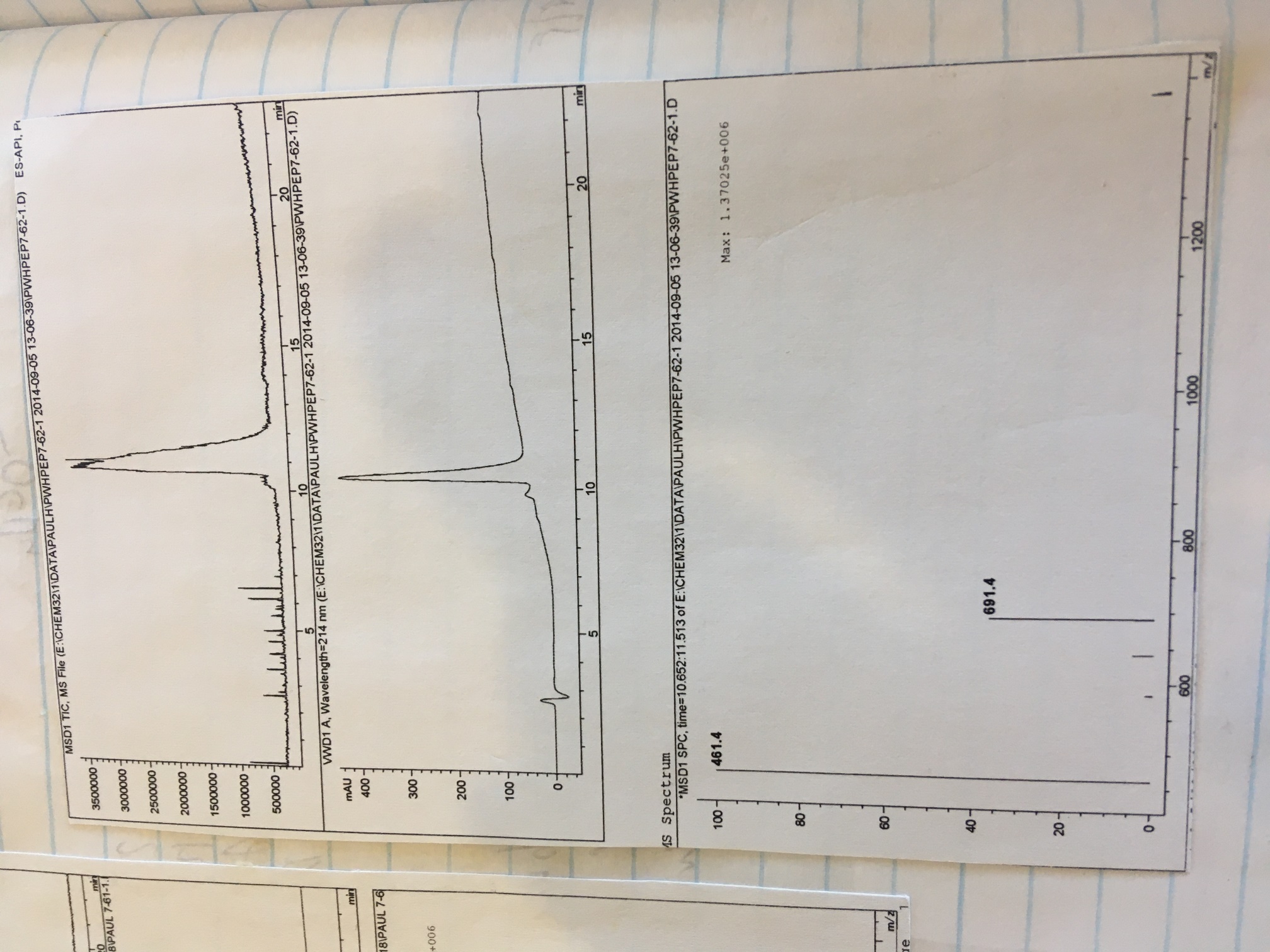


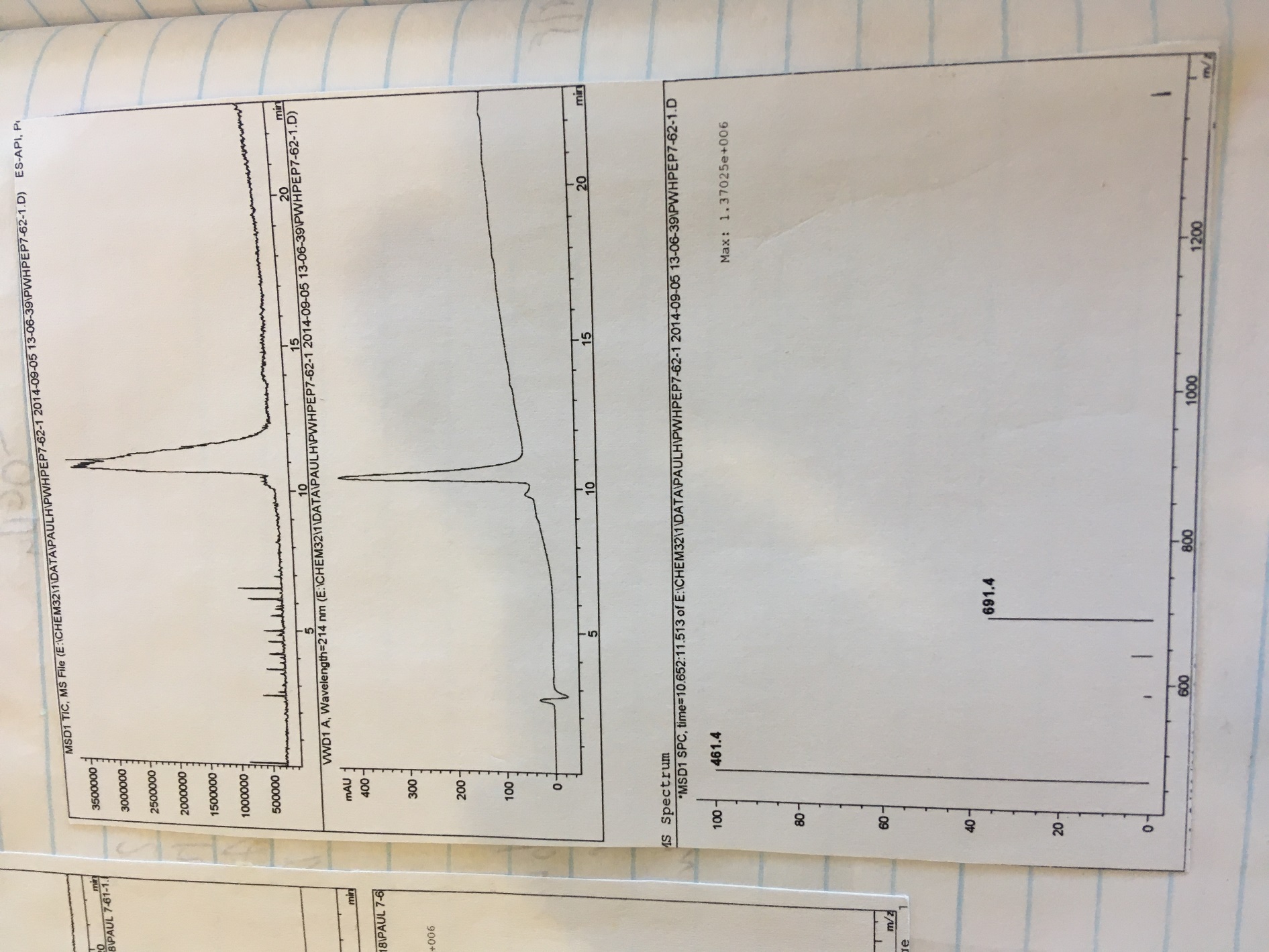


**Figure S21.** HPLC-MS spectrum of peptide **15**, *ca.* 97% purity as judged by peak area of RP-HPLC at 214 nm); Agilent C3-300SB (3.5 μm, 300 Å, 3 mm × 150 mm), linear gradient of 5% B to 65% B over 21 min, *ca.* 3% B per minute at 0.3 mL min^-1^; **MS** (ESI+) *m/z* 691.4 (calcd. For [M+2H]^2+^, 691.4), 461.4 (calcd. for [M+3H]^3+^,461.2).

***Peptide 16:***


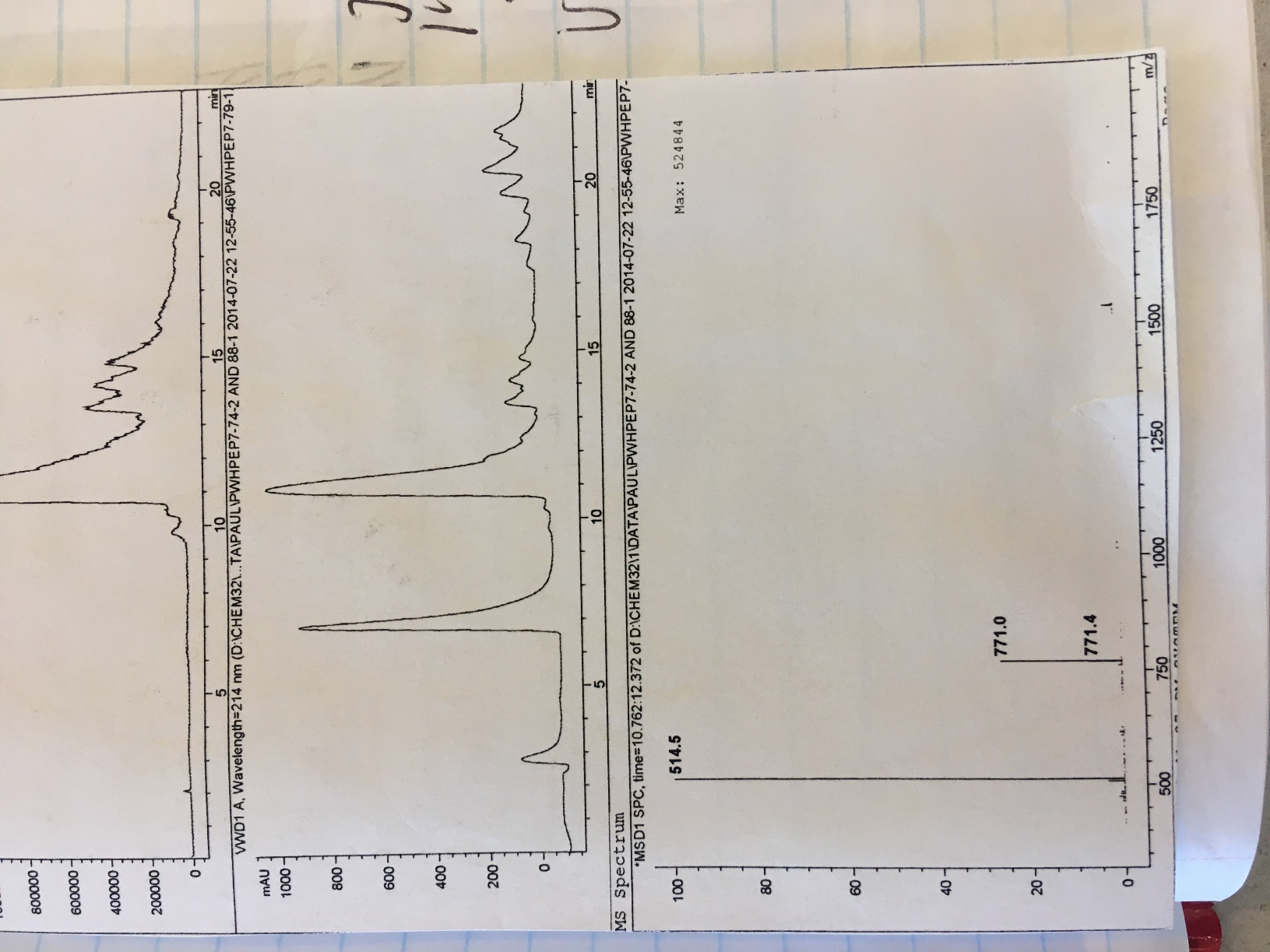


**Figure S22.** HPLC and ESI-MS spectrum of peptide **16**, *ca.* 97% purity as judged by peak area of RP-HPLC at 214 nm); Phenomenex (3.5 μm, 4.6 mm × 150 mm), linear gradient of 5% B to 65% B over 21 min, *ca.* 3% B per minute at 1 mL min^-1^; **MS** (ESI+) *m/z* 771.0 (calcd. For [M+2H]^2+^, 770.9), 514.5 (calcd. for [M+3H]^3+^, 514.3).

***Peptide 21***:


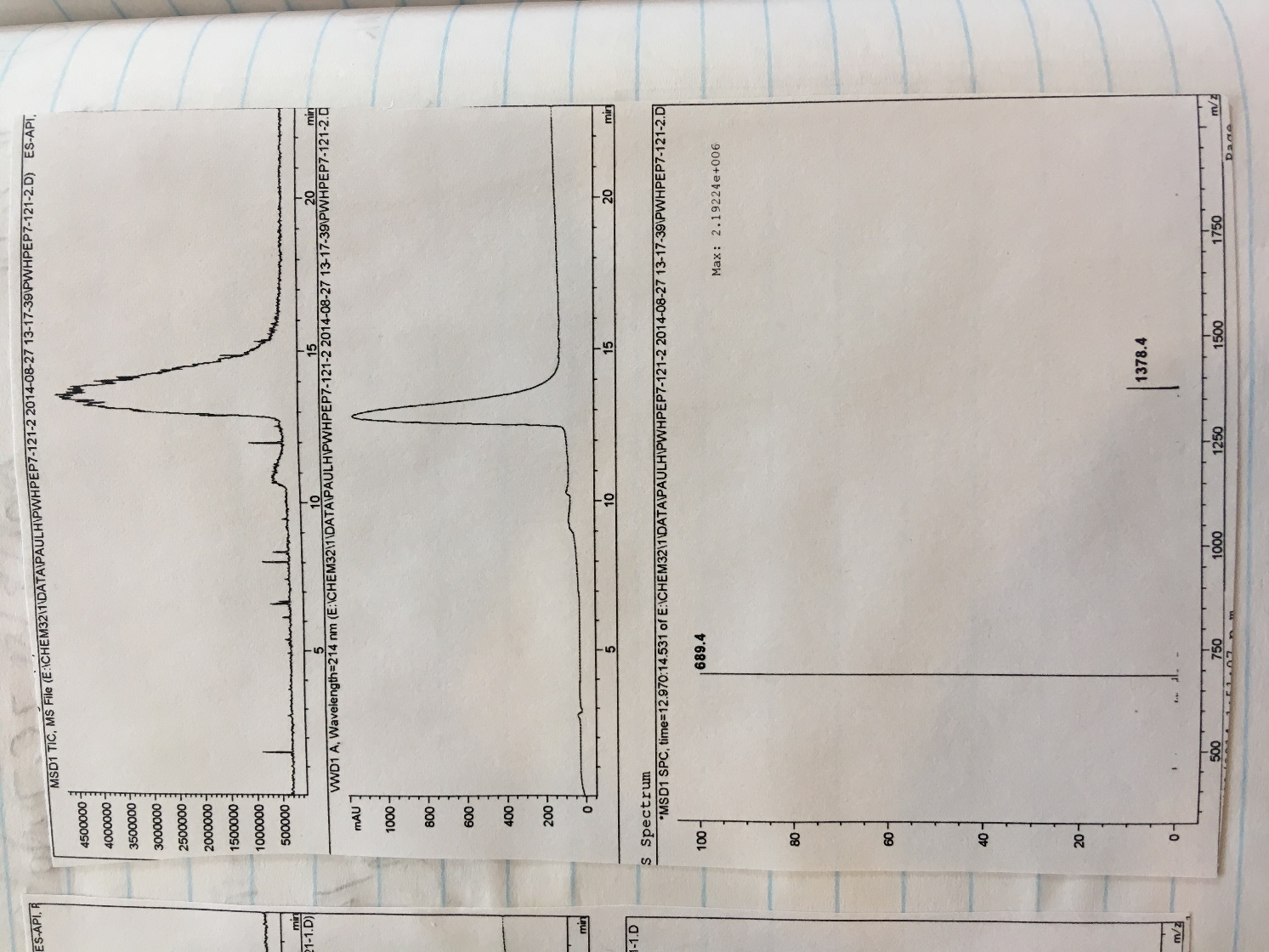


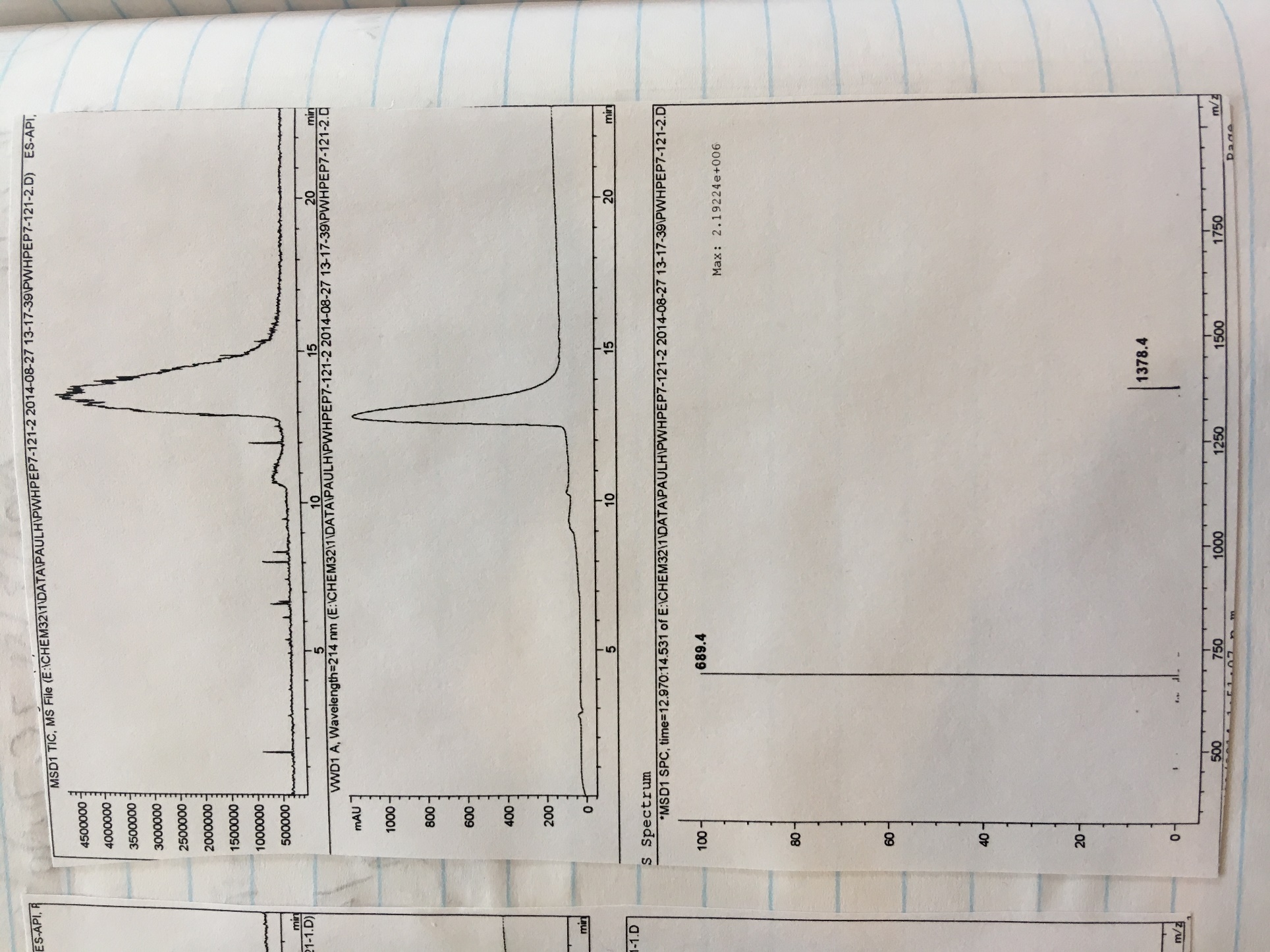


**Figure S23.** HPLC-MS spectrum of peptide **21**, *ca.* 99% purity as judged by peak area of RP-HPLC at 214 nm); Agilent C3-300SB (3.5 μm, 300 Å, 3 mm × 150 mm), linear gradient of 5% B to 65% B over 21 min, *ca.* 3% B per minute at 0.3 mL min^-1^; **MS** (ESI+) *m/z* 1378.4 (calcd. For [M+H]^+^, 1378.6), 689.4(calcd. For [M+2H]^2+^, 689.8)

***Peptide 22***:

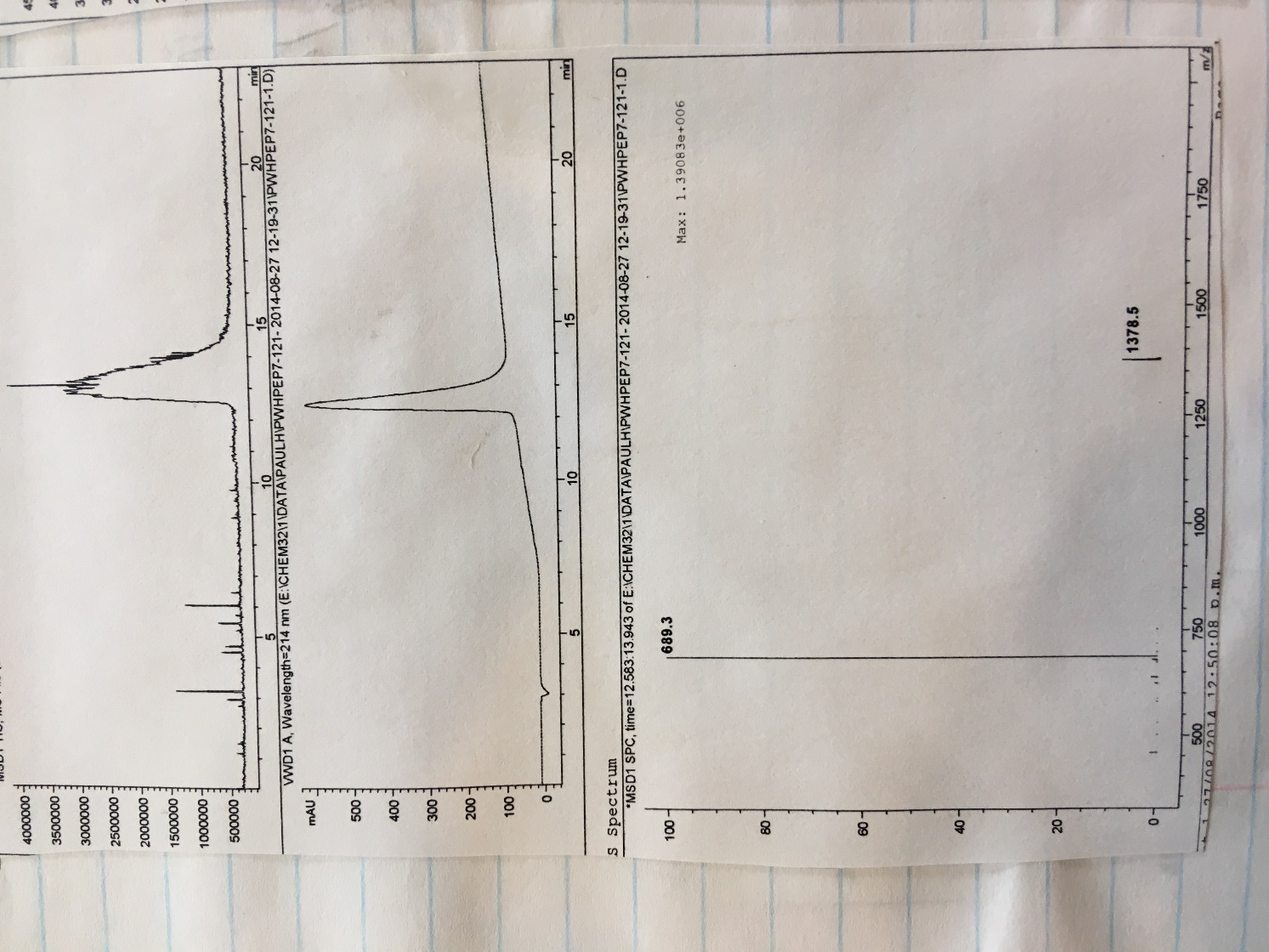


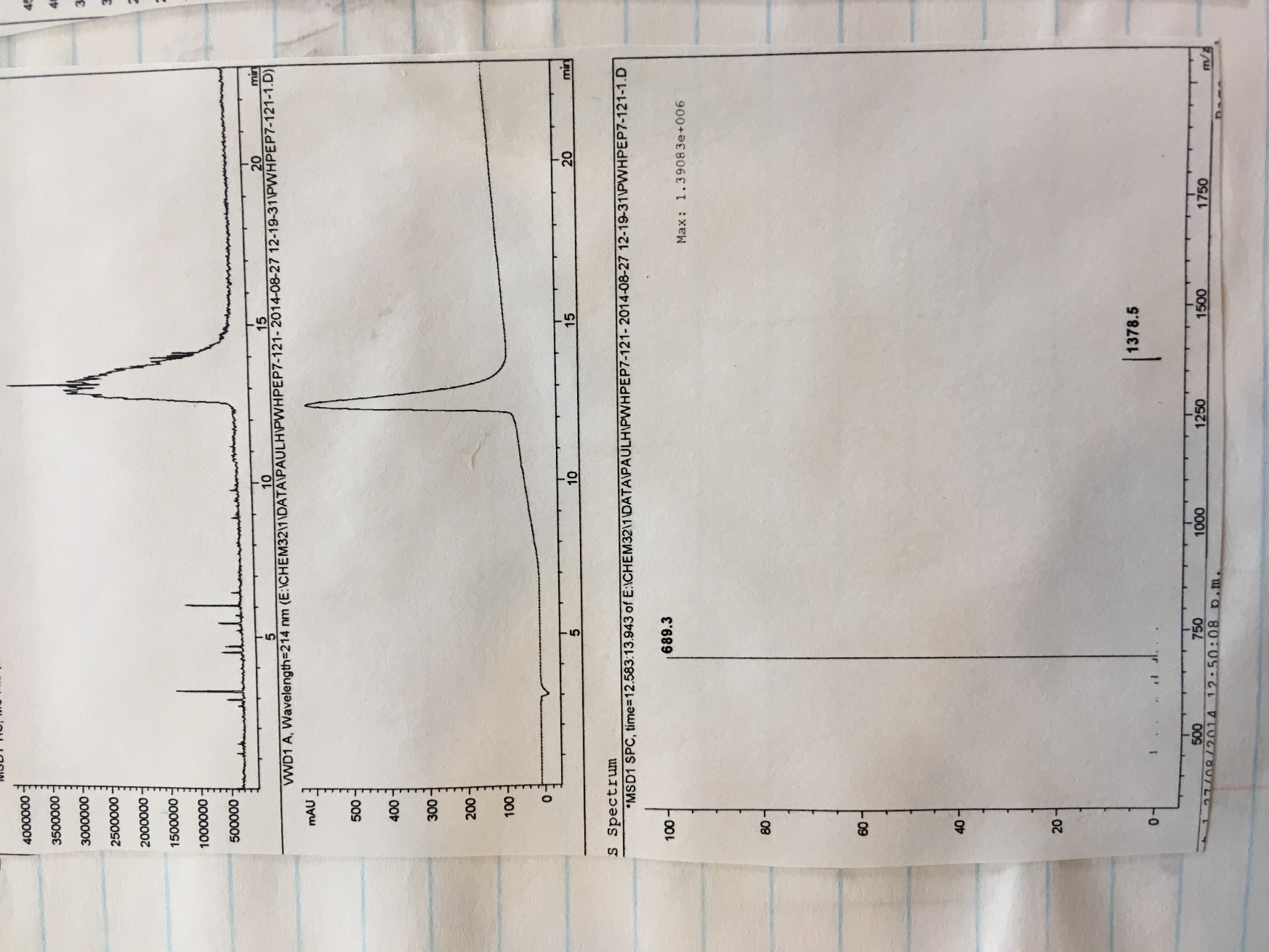


**Figure S24.** HPLC-MS spectrum of peptide **22**, *ca.* 99% purity as judged by peak area of RP-HPLC at 214 nm); Agilent C3-300SB (3.5 μm, 300 Å, 3 mm × 150 mm), linear gradient of 5% B to 65% B over 21 min, *ca.* 3% B per minute at 0.3 mL min^-1^; **MS** (ESI+) *m/z* 1378.5 (calcd. For [M+H]^+^, 1378.6), 689.3 (calcd. For [M+2H]^2+^, 689.8)

***Peptide 25:***

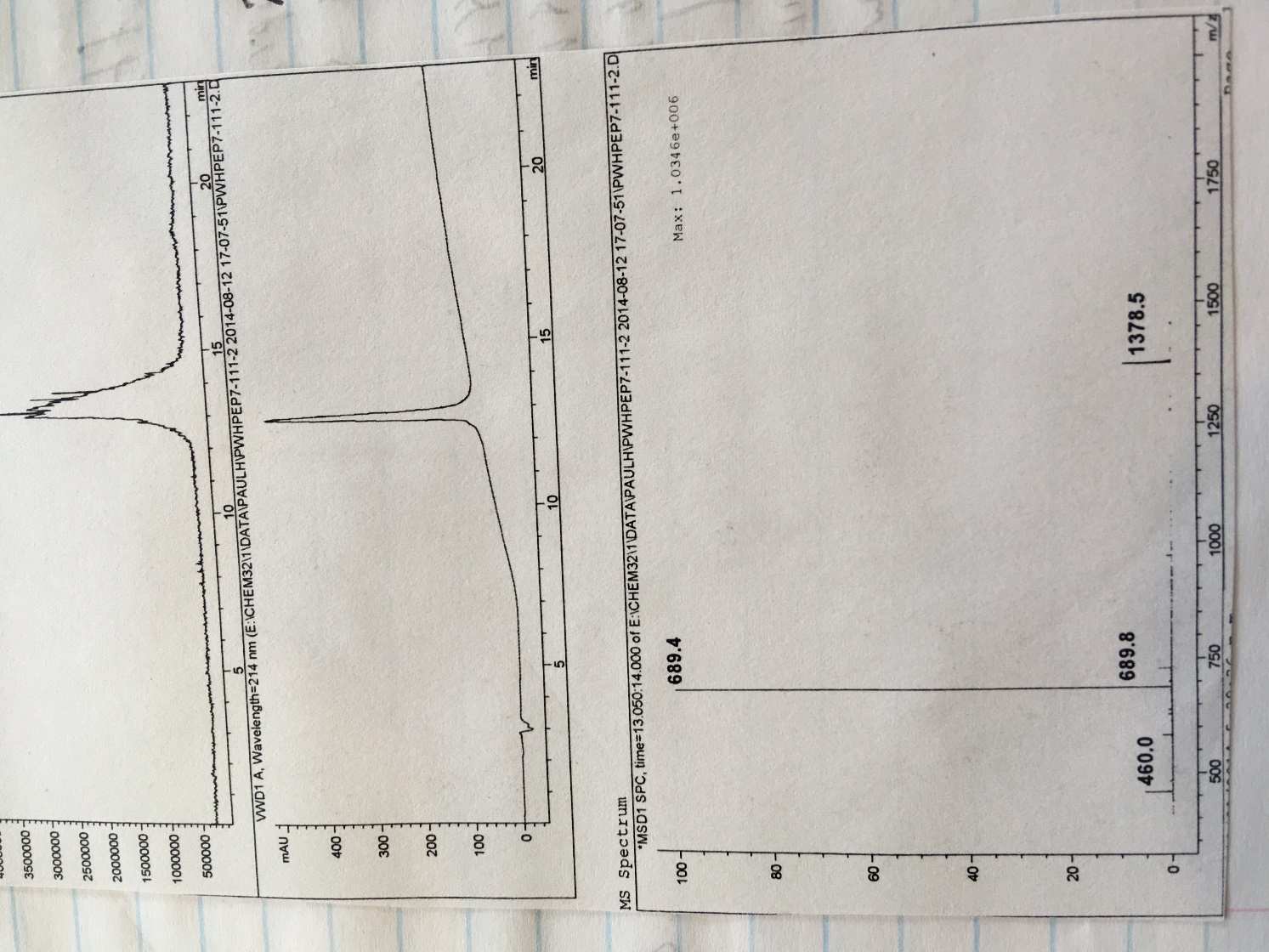


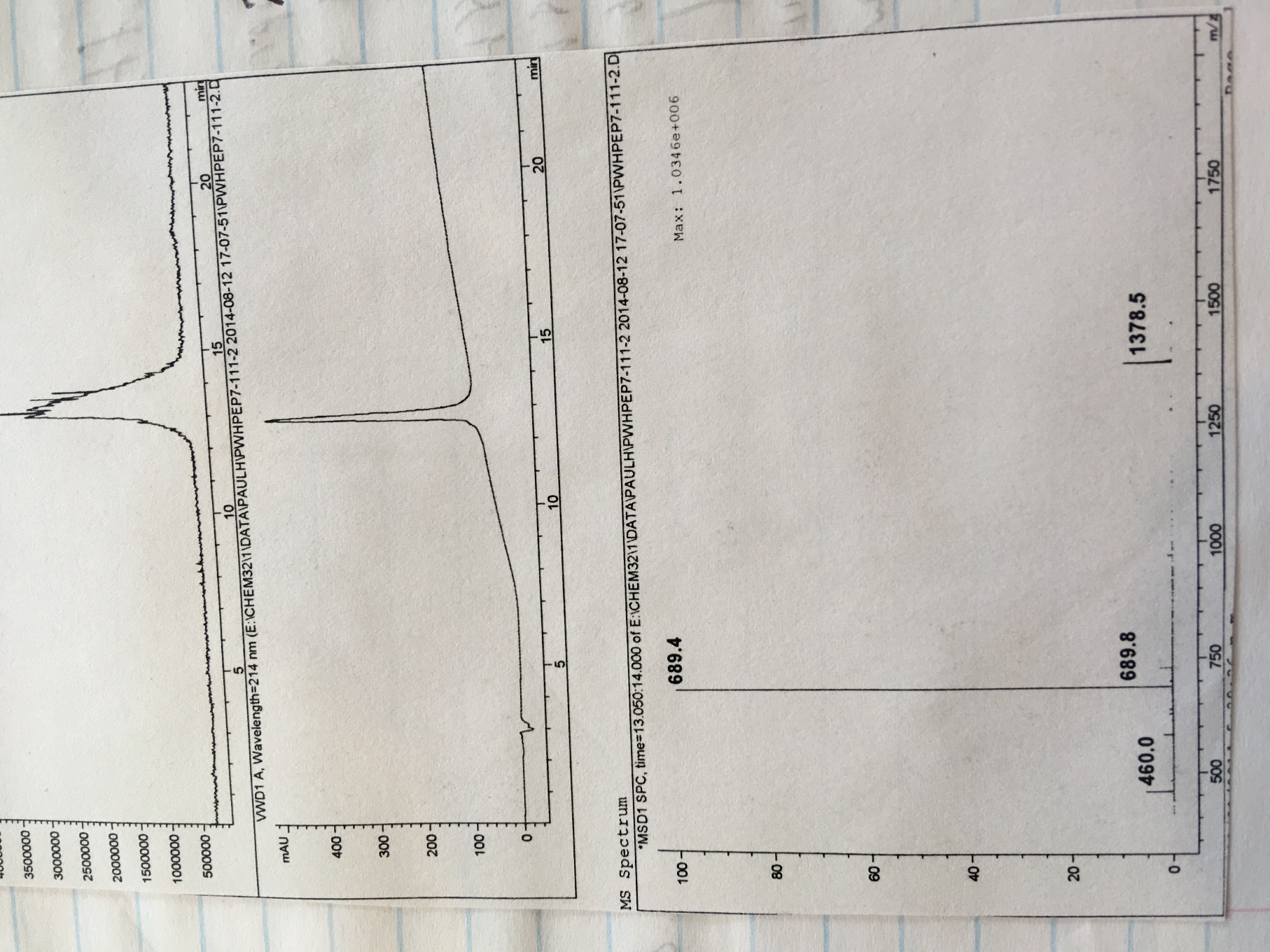


**Figure 25.** HPLC-MS spectrum of peptide **25**, *ca.* 99% purity as judged by peak area of RP-HPLC at 214 nm); Agilent C3-300SB (3.5 μm, 300 Å, 3 mm × 150 mm), linear gradient of 5% B to 65% B over 21 min, *ca.* 3% B per minute at 0.3 mL min^-1^; **MS** (ESI+) *m/z* 1378.5 (calcd. For [M+H]^+^, 1378.6), 689.4 (calcd. For [M+2H]^2+^, 689.8)

***Peptide 28:***

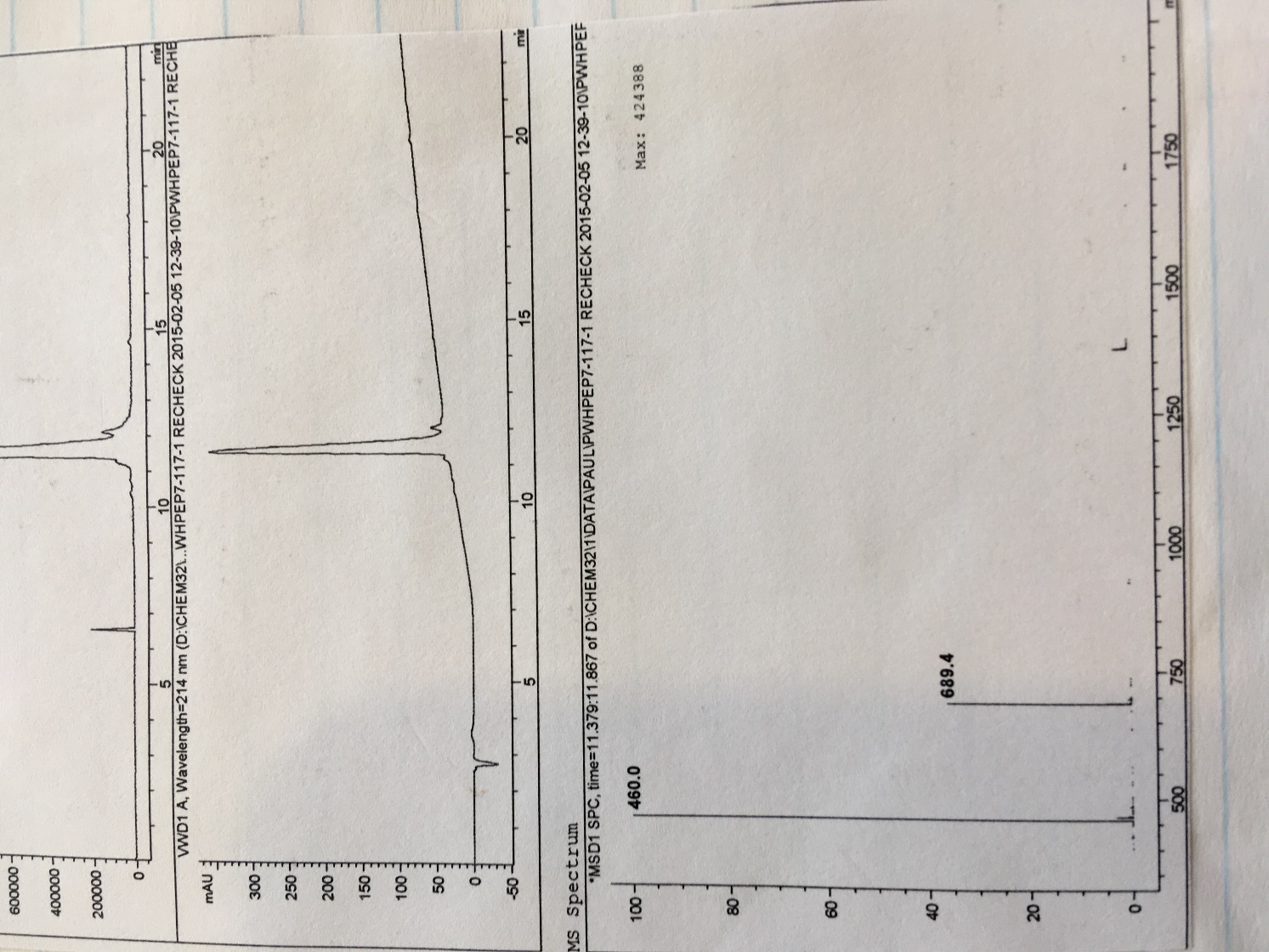


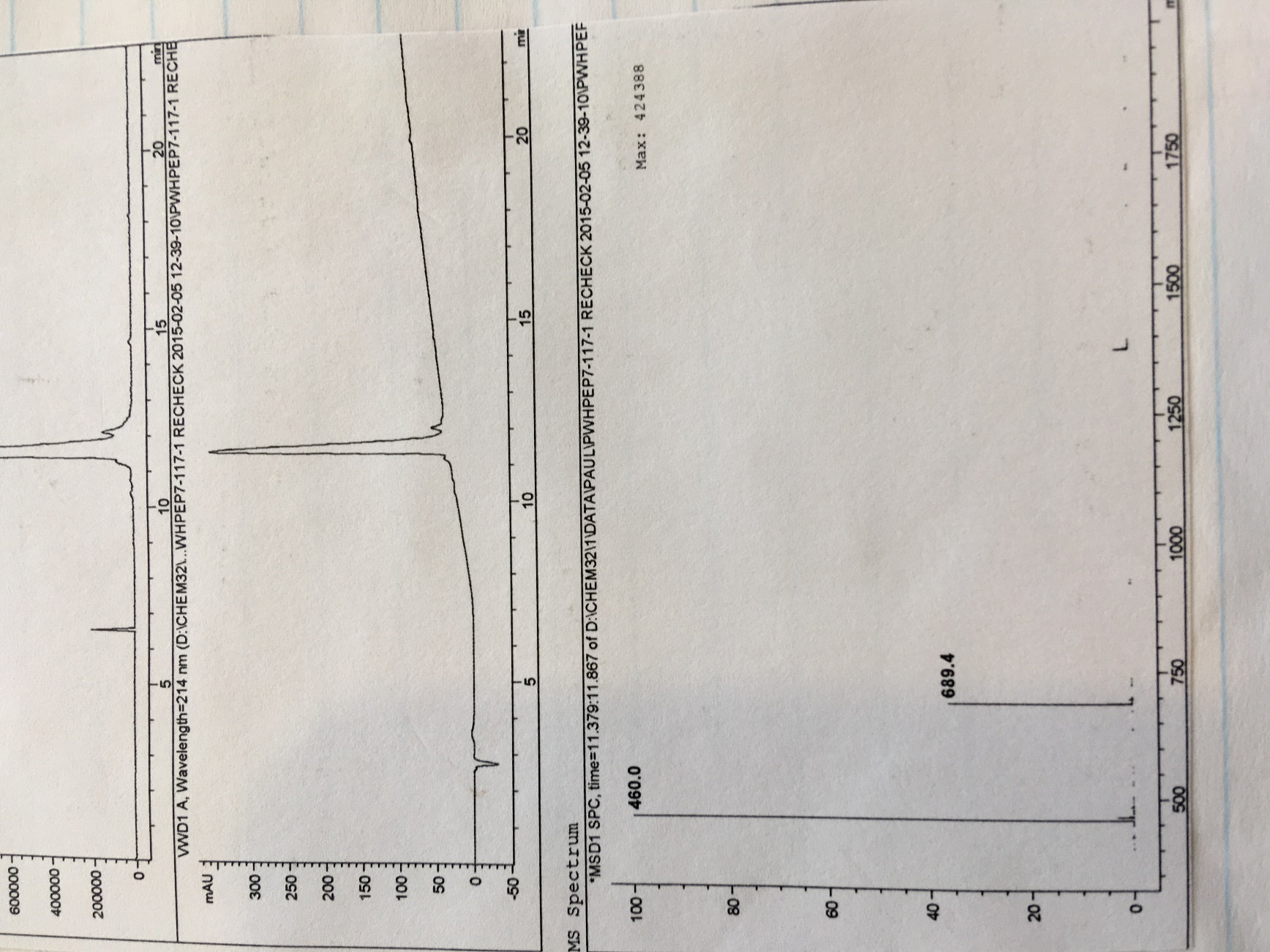


**Figure S26.** HPLC-MS spectrum of peptide **28**, *ca.* 96% purity as judged by peak area of RP-HPLC at 214 nm); Agilent C3-300SB (3.5 μm, 300 Å, 3 mm × 150 mm), linear gradient of 5% B to 65% B over 21 min, *ca.* 3% B per minute at 0.3 mL min^-1^; **MS** (ESI+) *m/z* 689.4 (calcd. For [M+2H]^2+^, 689.8), 460.6 (calcd. For [M+3H]^3+^, 460.2)

***Peptide 32:***

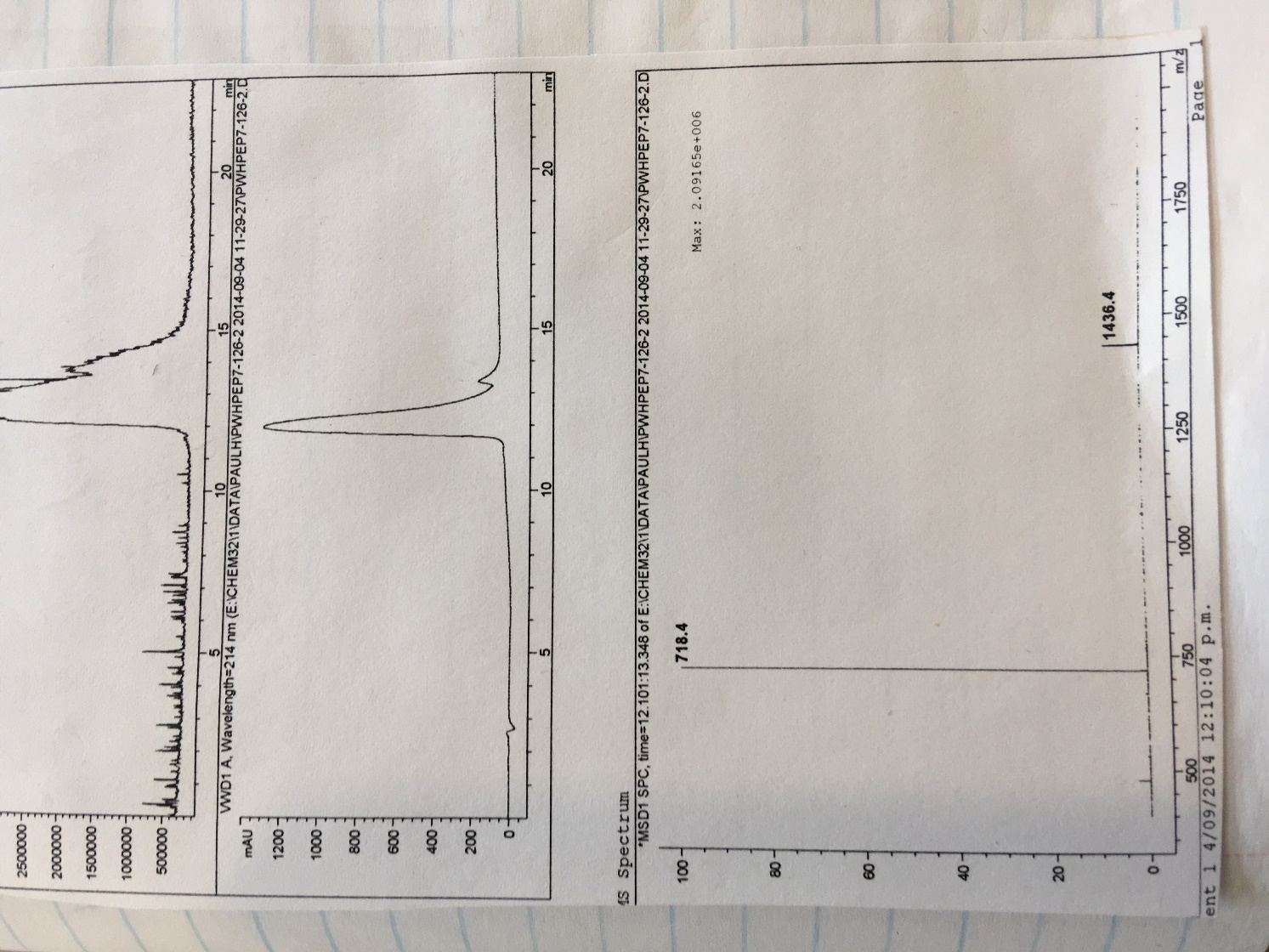


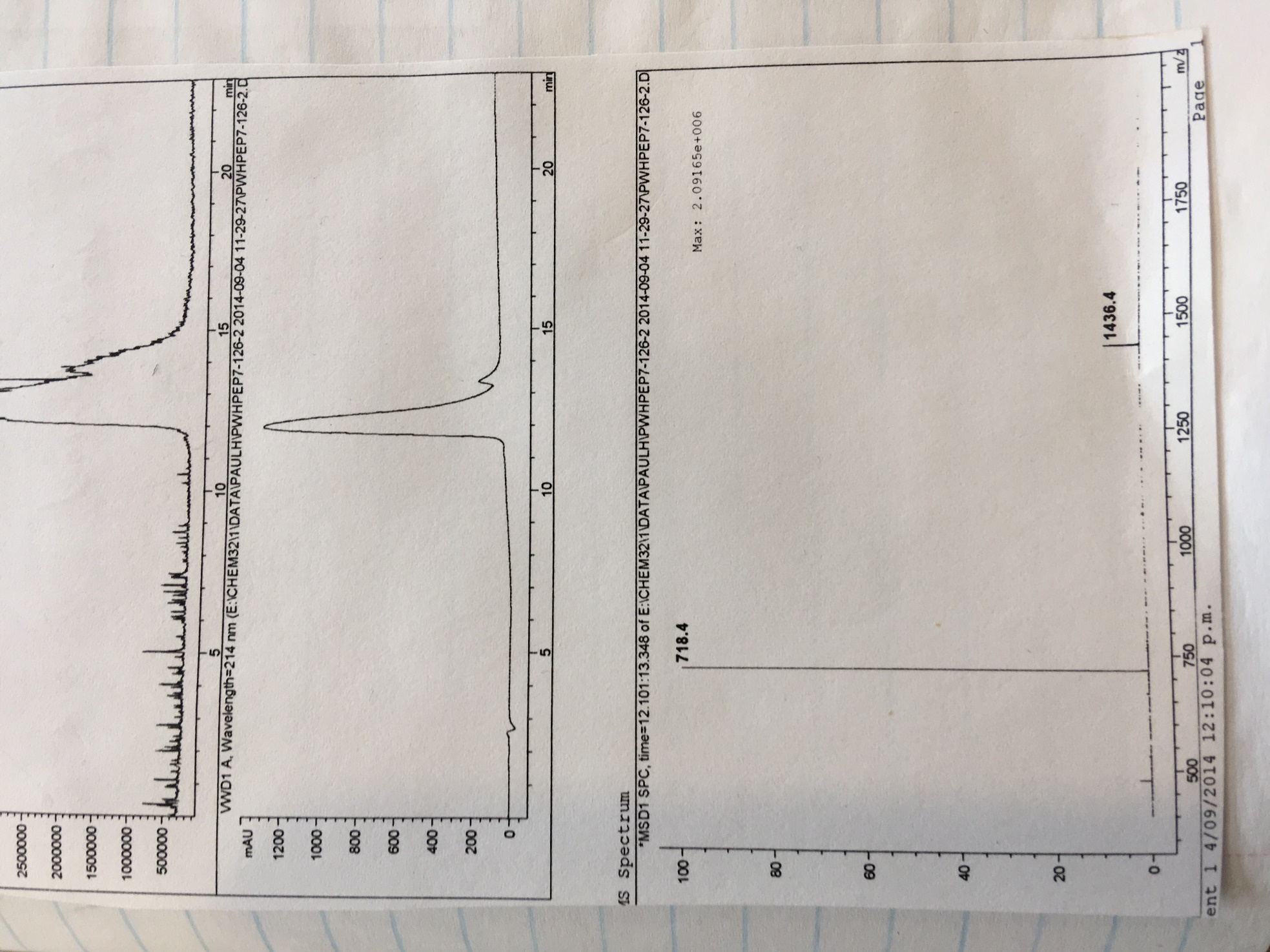


**Figure S27.** HPLC-MS spectrum of peptide **32**, *ca.* 96% purity as judged by peak area of RP-HPLC at 214 nm); Agilent C3-300SB (3.5 μm, 300 Å, 3 mm × 150 mm), linear gradient of 5% B to 65% B over 21 min, *ca.* 3% B per minute at 0.3 mL min^-1^; **MS** (ESI+) *m/z* 1436.4 (calcd. For [M+H]^+^, 1436.6), 718.4 (calcd. For [M+2H]^2+^, 718.2)

***Peptide 34:***

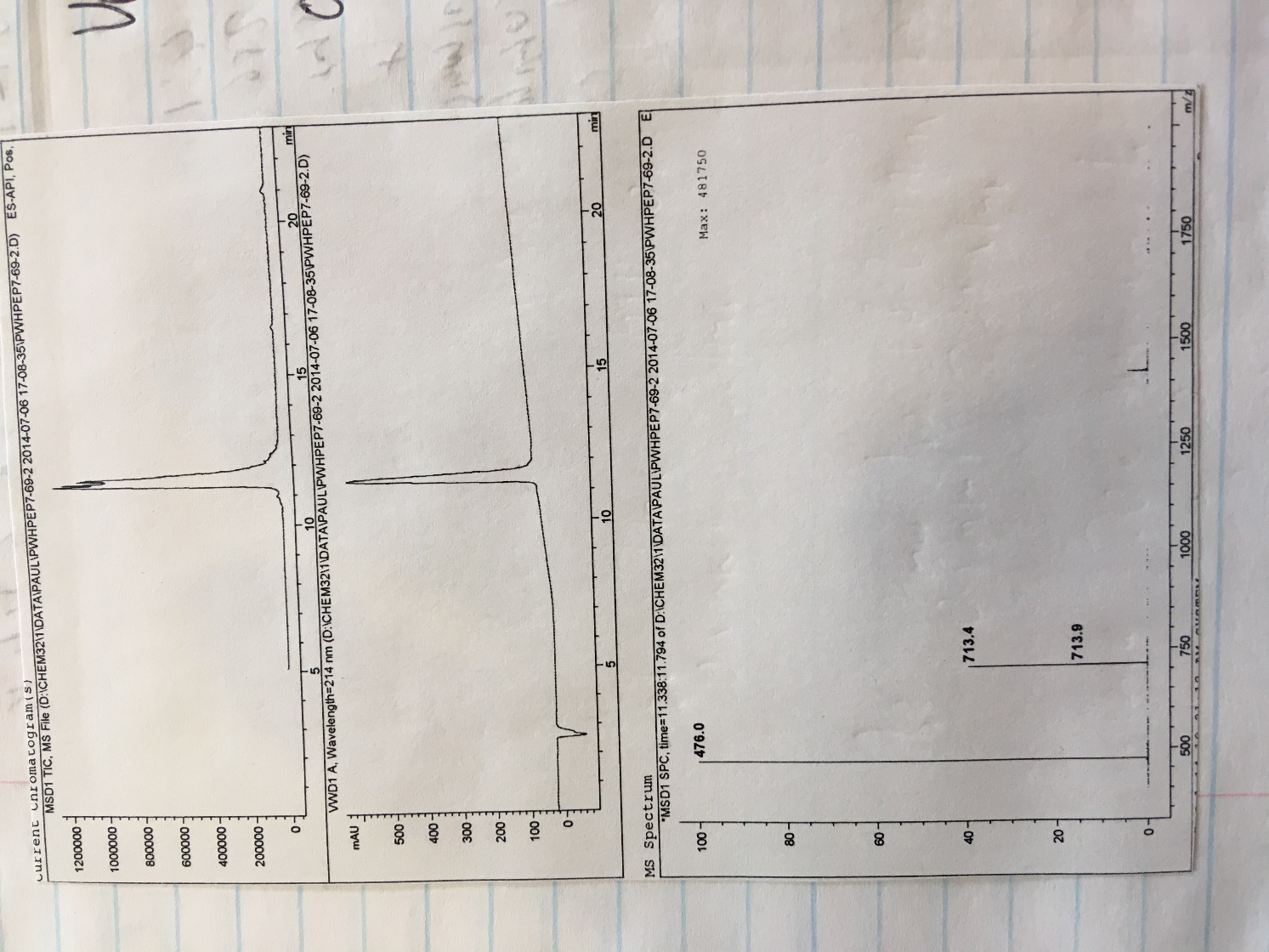


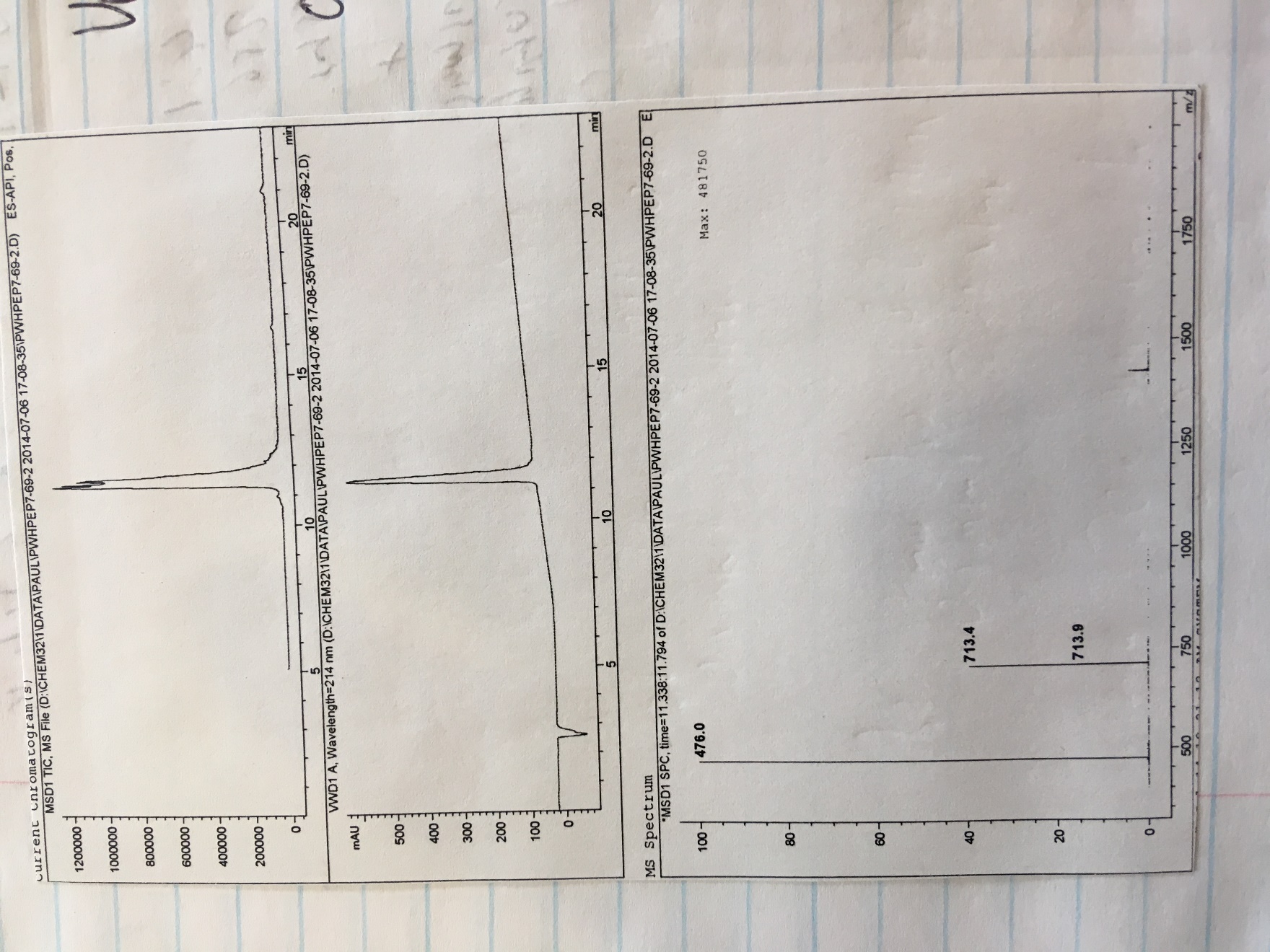


**Figure S28.** HPLC-MS spectrum of peptide **34**, *ca.* 99% purity as judged by peak area of RP-HPLC at 214 nm); Agilent C3-300SB (3.5 μm, 300 Å, 3 mm × 150 mm), linear gradient of 5% B to 65% B over 21 min, *ca.* 3% B per minute at 0.3 mL min^-1^; **MS** (ESI+) *m/z* 713.4 (calcd. For [M+2H]^2+^, 713,8), 476.0 (calcd. For [M+3H]^3+^, 476.2)

***Peptide 37:***

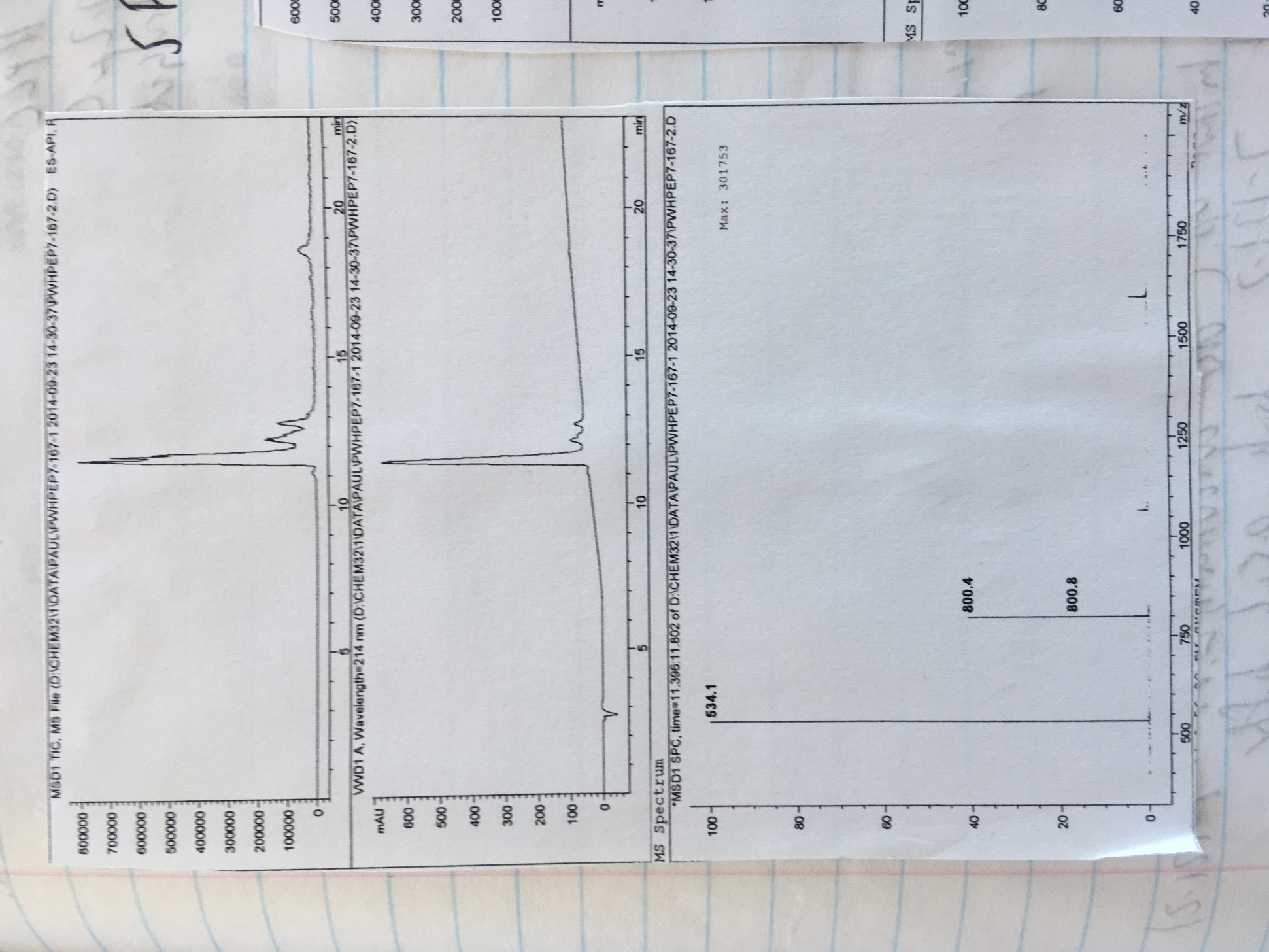


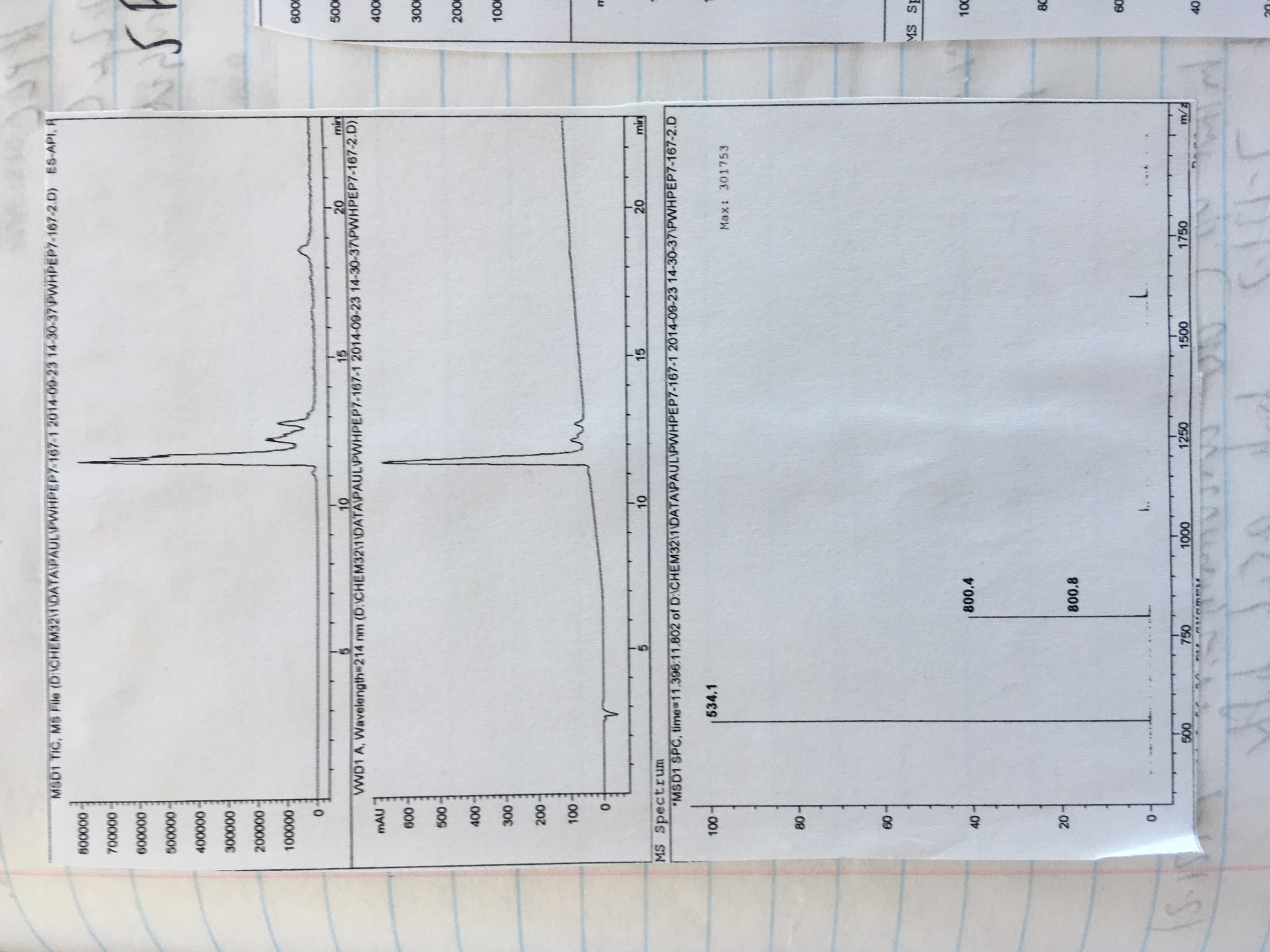


**Figure S29.** HPLC-MS spectrum of peptide **37**, *ca.* 95% purity as judged by peak area of RP-HPLC at 214 nm); Agilent C3-300SB (3.5 μm, 300 Å, 3 mm × 150 mm), linear gradient of 5% B to 65% B over 21 min, *ca.* 3% B per minute at 0.3 mL min^-1^; **MS** (ESI+) *m/z* 800.4 (calcd. For [M+2H]^2+^, 800.9), 534.1 (calcd. For [M+3H]^3+^, 534.3)

***Peptide 39:***


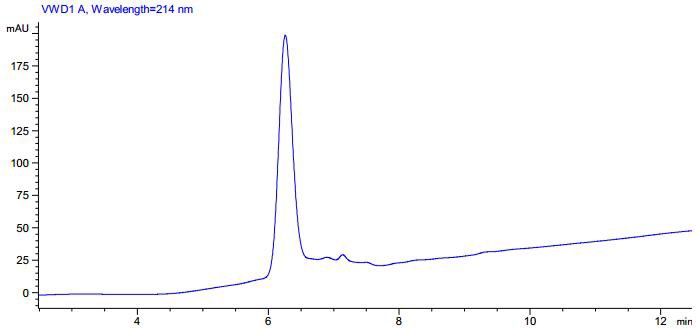


**Figure S30** HPLC of purified peptide **39**, *ca.* 95% purity as judged by peak area of RP-HPLC at 214 nm); Phenomenex Gemini C18 (5µ 110Å 2.0 x 50 mm) linear gradient of 1%B to 61% B over 21 mins. **MS** (ESI+) *m/z* 1560.0 (calcd. For [M+H]^+^, 1561.8)

***Peptide 37:***


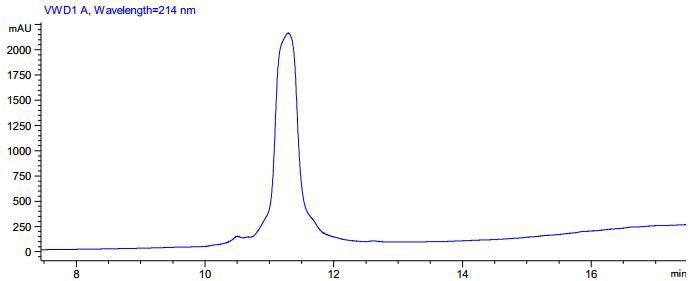


**Figure S31** HPLC of purified peptide **40**, *ca.* 95% purity as judged by peak area of RP-HPLC at 214 nm); Phenomenex Gemini C18 (5µ 110Å 2.0 x 50 mm) linear gradient of 1%B to 61% B over 21 mins. **MS** (ESI+) *m/z* 1433.5 (calcd. For [M+H]^+^, 1433.7

***Peptide 42:***


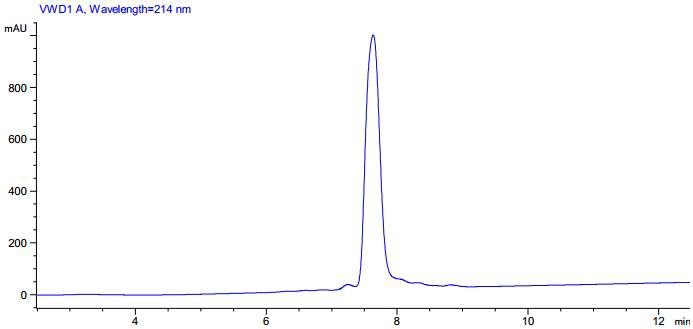


**Figure S32** HPLC of purified peptide **42**, *ca.* 95% purity as judged by peak area of RP-HPLC at 214 nm); Phenomenex Gemini C18 (5µ 110Å 2.0 x 50 mm) linear gradient of 1%B to 61% B over 21 mins. **MS** (ESI+) *m/z* 1436.6 (calcd. For [M+H]^+^, 1433.7)

**References**

Bondarenko, A., & Chesler, M. (2001). Calcium dependence of rapid astrocyte death induced by transient hypoxia, acidosis, and extracellular ion shifts. *Glia, 34*(2), 143-149. doi:10.1002/glia.1049

el-Fouly, M. H., Trosko, J. E., & Chang, C. C. (1987). Scrape-loading and dye transfer. A rapid and simple technique to study gap junctional intercellular communication. *Exp Cell Res, 168*(2), 422-430.

Kim, Y., Griffin, J. M., Harris, P. W., Chan, S. H., Nicholson, L. F., Brimble, M. A., . . . Green, C. R. (2017). Characterizing the mode of action of extracellular Connexin43 channel blocking mimetic peptides in an in vitro ischemia injury model. *Biochim Biophys Acta Gen Subj, 1861*(2), 68-78. doi:10.1016/j.bbagen.2016.11.001

O'Carroll, S. J., Kho, D. T., Wiltshire, R., Nelson, V., Rotimi, O., Johnson, R., . . . Graham, E. S. (2015). Pro-inflammatory TNFalpha and IL-1beta differentially regulate the inflammatory phenotype of brain microvascular endothelial cells. *J Neuroinflammation, 12*(1), 131. doi:10.1186/s12974-015-0346-0
